# The Development of a Smart Magnetic Resonance Imaging and Chemical Exchange Saturation Transfer Contrast Agent for the Imaging of Sulfatase Activity

**DOI:** 10.3390/ph16101439

**Published:** 2023-10-11

**Authors:** Ilse M. Welleman, Friederike Reeβing, Hendrikus H. Boersma, Rudi A. J. O. Dierckx, Ben L. Feringa, Wiktor Szymanski

**Affiliations:** 1 Department of Radiology, Medical Imaging Center, University Medical Center Groningen, University of Groningen, Hanzeplein 1, 9713 GZ Groningen, The Netherlands; i.m.welleman@umcg.nl (I.M.W.);; 2 Stratingh Institute for Chemistry, University of Groningen, Nijenborgh 4, 9747 AG Groningen, The Netherlands; 3 Department of Clinical Pharmacy and Pharmacology, Nuclear Medicine and Molecular Imaging, University Medical Center Groningen, University of Groningen, Hanzeplein 1, 9713 GZ Groningen, The Netherlands

**Keywords:** molecular imaging, sulfatase, MRI, CEST, responsive imaging agents

## Abstract

The molecular imaging of biomarkers plays an increasing role in medical diagnostics. In particular, the imaging of enzyme activity is a promising approach, as it enables the use of its inherent catalytic activity for the amplification of an imaging signal. The increased activity of a sulfatase enzyme has been observed in several types of cancers. We describe the development and in vitro evaluation of molecular imaging agents that allow for the detection of sulfatase activity using the whole-body, non-invasive MRI and CEST imaging methods. This approach relies on a responsive ligand that features a sulfate ester moiety, which upon sulfatase-catalyzed hydrolysis undergoes an elimination process that changes the functional group, coordinating with the metal ion. When Gd^3+^ is used as the metal, the complex can be used for MRI, showing a 25% decrease at 0.23T and a 42% decrease at 4.7T in magnetic relaxivity after enzymatic conversion, thus providing a “switch-off” contrast agent. Conversely, the use of Yb^3+^ as the metal leads to a “switch-on” effect in the CEST imaging of sulfatase activity. Altogether, the results presented here provide a molecular basis and a proof-of-principle for the magnetic imaging of the activity of a key cancer biomarker.

## 1. Introduction

Molecular imaging is a multidisciplinary field that aims to enable the non-invasive, real-time visualization of complex physiological processes on a molecular level in living subjects. Molecular imaging provides clinicians with a tool to image characteristics and biomarkers of a disease, making it a valuable alternative for invasive biopsies procedures [1]. There are different techniques available in the clinical application of molecular imaging, including single-photon emission computed tomography (SPECT), positron emission tomography (PET), optical imaging (OI), and molecular magnetic resonance imaging (mMRI) [2]. The choice of the technique depends on the target and application, as each imaging modality has advantages and disadvantages, depending on its spatial resolution, penetration depth, and sensitivity. 

The use of molecular imaging is of particular interest for detecting enzyme activity since the increased activity of certain enzymes (such as matrix metalloproteinases [3], caspase-3 [3], and tyrosine kinases [4]) is a biomarker for cancer. The imaging of enzymatic activity also enables increasing the sensitivity through the amplification of the signal via catalytic activation of the imaging probe by the enzyme, potentially allowing the use of low-sensitivity imaging methods, such as magnetic resonance imaging (MRI) or chemical exchange saturation transfer (CEST) imaging. Here, we report the development of a paramagnetic CEST (paraCEST)/MRI contrast agent for the imaging of sulfatase activity. 

Sulfatases are enzymes that belong to the esterase class and are known to catalyze the hydrolysis of sulfate esters in a variety of substrates, including glycosaminoglycans, glycolipids, and steroids [5,6]. For example, steroid sulfatase is responsible for the hydrolysis of aryl and alkyl steroid sulfates and plays a significant role in the formation of biologically active steroids, particularly in the synthesis of estradiol. Although the enzyme is widely distributed throughout the human body, its overexpression has been associated with several pathological conditions, including certain hormonal (breast, endometrial, prostate, and ovarian) cancers, making it an attractive target for the development of smart, activatable imaging agents for those malignancies [5,7,8].

In recent years, different molecular imaging modalities have been applied to the detection of sulfatase activity. Nuclear (PET/SPECT) imaging techniques have been used, offering unparalleled sensitivity but lacking specificity due to relying on the irreversible covalent binding of the tracer to an enzyme, which is not selective since it was also found to covalently bind to carbonic anhydrase, primarily found in blood [9,10,11]. Furthermore, optical and optoacoustic probes were recently presented [12] and applied for inhalation-based, imaging-guided surgical excision of lung cancer [13]. However, while providing good sensitivity and selectivity, optical imaging modalities are limited to intraoperative settings since the light used to image is widely scattered and absorbed in the human body, resulting in poor imaging depth and spatial resolution.

MRI, which is a non-invasive, whole-body imaging technique with outstanding spatial resolution [14,15], provides a potential solution to this challenge and could be a method of choice for image sulfatase. However, compared to nuclear and optical imaging methods, MRI has a very low sensitivity that severely limits its application in visualizing physiological processes and renders it a primarily anatomical imaging method [15,16,17]. Nevertheless, in the particular case of enzyme activity imaging, this limitation could be overcome with the use of enzyme-responsive MRI contrast agents (contrast agents (CAs), which are most often the complexes of paramagnetic metal ions, such as Gd^3+^ or Mn^2+^) that produce or change the imaging signal (i.e., magnetic relaxivity) due to an enzyme-induced change in their chemical structure [16]. 

According to the Solomon–Bloembergen–Morgan theory [18,19], there are three main structural parameters that define the relaxivity of an MRI CA, all of which can be utilized in designing a responsive molecule and include (1) the number of water molecules binding to the metal center (water coordination sides, q), (2) the residence time of the coordinated water molecule on to the lanthanide complex (represented by τ_m_), and (3) the tumbling of a molecule (represented by τ_R_). An example of an enzyme-responsive MRI contrast agent, in which an enzyme-induced change in relaxivity is believed to mainly stem from the change in coordination sites, has been reported by Moats et al. [20], who described a galactose-modified Gd^3+^ complex that shows a decrease in relaxivity upon hydrolysis by β-galactosidase. In another, more recent example of galactosidase activity imaging [21], an increase in relaxivity was achieved upon enzymatic hydrolysis. An example of the modulation of the rotational correlation time was shown by Nivorozhkin et al., where the contrast agent releases a protecting group upon activation by carboxypeptidase B, revealing a binding site for human serum albumin, which led to an increase in relaxivity [22]. However, it is usually quite challenging to develop “positive” contrast agents, i.e., those that increase their signal upon activation, because the factors that contribute to a rise in relaxivity are difficult to predict, as the contrast agent can bind to proteins or aggregate, resulting in a change in relaxivity, as outlined in seminal reviews by Wahsner et al. [19] and Hingorani et al. [16]. To the best of our knowledge, a sulfatase-responsive MRI contrast agent has not been reported in the literature.

**Figure 1:**
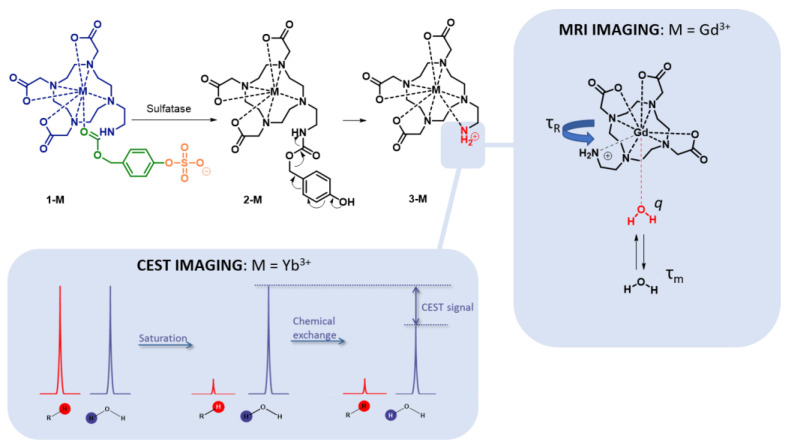
Schematic overview of the activation of the MRI and CEST contrast agents presented by the sulfatase enzyme. **1-M** is hydrolyzed by the sulfatase enzyme to compound **2-M**, which is unstable and will form compound **3-M** via 1,6-elimanation, liberating the amine group.

The design of “positive” contrast agents is much more facile with CEST (chemical exchange saturation transfer) imaging, an emerging MRI modality that enables the sensitive imaging of physiological factors (pH, temperature, enzyme activity, receptor expression) [18,19,23]. Unlike conventional MRI, CEST does not rely on the change in the relaxation rate of the water protons induced by the contrast agent. Instead, it is based on the exchange of magnetically saturated protons between the contrast agent and the bulk water molecules surrounding them. In essence, one proton pool (the contrast agent) is first selectively statured by applying a corresponding radiofrequency pulse (Figure 1), followed by a chemical exchange between the protons from the contrast agent pool and the bulk water pool, which leads to a reduced signal of the bulk water proton pool, resulting in a signal that can be transformed to an image [19,23]. 

To enable the CEST effect, a CEST CA needs to feature exchangeable protons in functional groups such as alcohols, amines, amides, and acids, and those protons need to resonate in the range of Larmor frequencies that allow them to be selectively saturated [19,23]. CEST contrast agents can be divided into two groups: diamagnetic (diaCEST) and paramagnetic (paraCEST) contrast agents. An example of a diaCEST contrast agent for sulfatase has been published by Sinharay et al. [24], who presented a molecule that produces a CEST signal at *δ* = 5.0 ppm before and at *δ* = 9.0 ppm after enzymatic hydrolysis of the sulfate group. The advantage of paraCEST is that thanks to the introduction of a paramagnetic metal into the CA, the exchangeable protons of the contrast agent are shifted further away from the bulk water signal, allowing them to be saturated selectively without affecting the bulk water and other molecules that are present in the human body. Another advantage of a large chemical shift difference is that it allows for observing faster proton exchange with the bulk water, resulting in a larger CEST effect. [19]

Recognizing the complementary advantages of MRI and CEST imaging, and the similar nature of metal complexes that can act as imaging agents in these two methods, we describe the parallel development of activatable contrast agents (**1-M**, Figure 1) that can be used for the imaging of sulfatase activity. Depending on the type of metal ion coordinated to the central DOTA-type ligand in **1-M**, these CAs can be used either for MRI (M = Gd^3+^) or CEST (M = Yb^3+^) imaging.

## 2. Results

### 2.1. Design and Synthesis of Contrast Agents 1-Gd and 1-Yb

**Figure 2:**
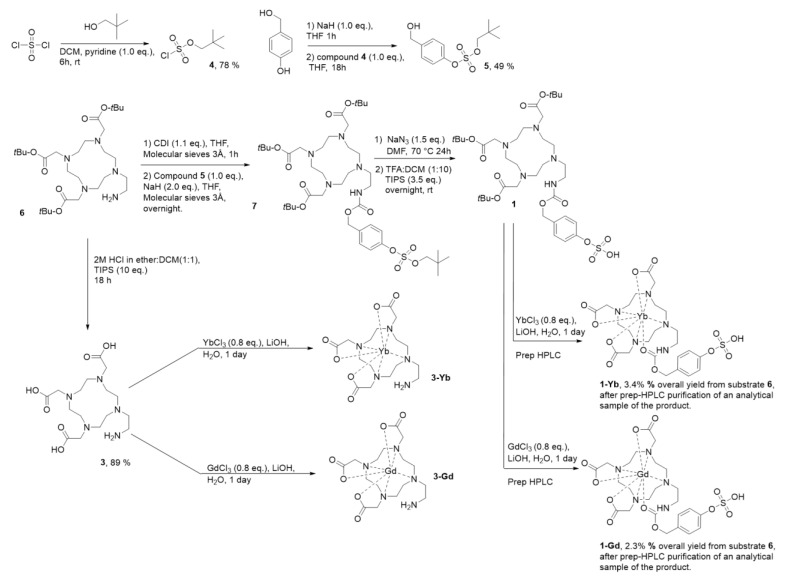
Synthetic route toward compounds **1-Gd** and **1-Yb** and the model products of their enzymatic hydrolysis, compounds **3-Gd** and **3-Yb**.

The structure of compound **1** features three key components (Figure 1): the cyclen-based ligand for a lanthanide (shown in blue, Figure 1), attached to the sulfatase-responsive sulfate ester (orange) through a self-immolation linker (in green), which has been introduced previously by Chauvin et al. [25]. We hypothesized that after hydrolysis of the sulfate group by sulfatase, the formed metastable compound **2-M** will undergo a 1,6-elimination process to form compound **3-M**. For MRI imaging, this overall process is expected to result in a change in magnetic relaxivity due to the changes in the number of water molecules bound to the metal center; while the carbamate carbonyl oxygen in **1-M** probably binds to the metal center, the nature of this bond is weak, as previously shown by Duimstra et al., for an analogous MRI CA designed for β-glucuronidase [26]. Conversely, the amine group in **3-M** has been postulated to have a higher binding affinity for the metal, provided that it is in a deprotonated state, as described by Giovenzana et al. [27]. For CEST imaging, it has been reported by Krchova et al. [28] that the amine group in compound **3-Yb** (shown in Figure 2) is able to generate CEST signals at ~40 and ~90 ppm due to the change in the position of the amine group, which moves closer to the metal ion and becomes better aligned to the magnetic axis. At the same time, we expected compound **1-Yb** to be CEST-silent in this range of the spectrum due to the masking of the amine group. 

The synthesis of ligand **1** (Figure 2) started with the reported preparation of sulfurochloridate **4**. Typically, a vacuum distillation is used for the purification of molecule **4** [29,30]. However, by lowering the equivalents of neopentyl alcohol used in the reaction from 1.4 to 1.0, we were able to purify the compound elegantly using a filtration step to remove the pyridinium salts, which resulted in compound **4** with a good yield (78%). Hereafter, compound **4** was coupled to 4-hydroxybenzyl alcohol to form the neopentyl-protected, sulfatase-responsive self-immolating linker **5**. Simultaneously, compound **6** was synthesized according to a published procedure [31] (see Supporting Information Section S2) and coupled to linker **5** through a carbamate linker using 1,1′-carbonyldiimidazole (CDI) and sodium hydride as a base. Subsequently, the neopentyl group was deprotected with sodium azide [30] and the *tert*-butyl esters were cleaved under acidic conditions, giving rise to the final ligand **1**. The purification of molecule **1**, and especially its separation from molecule **3** resulting from the deprotection of unreacted molecule **6**, proved to be highly challenging due to the very high polarity of all the compounds involved. Therefore, we opted to first insert the lanthanide metals to decrease the polarity and enable the purification of analytical samples of the resulting complexes **1-Gd** and **1-Yb** via preparative-HPLC. In parallel, the synthesis of compounds **3-Yb** and **3-Gd** was achieved, starting from compound **6.** First, under acidic conditions, the *tert*-butyl esters were cleaved, resulting in ligand **3.** The complexation of Gd^3+^ and Yb^3+^ into ligand **3** was performed following literature protocols [28].

### 2.2. Enzymatic Hydrolysis

**Figure 3:**
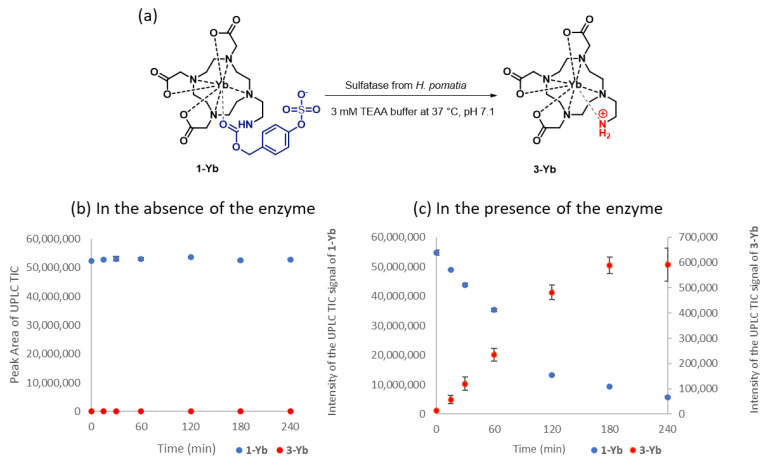
Evaluation of the stability and enzymatic conversion of complex **1-Yb**, using HPLC-MS analysis to follow the progress of its sulfatase-catalyzed hydrolysis toward **3-Yb**. (**a**) The sulfatase catalyzed hydrolysis of **1-Yb** toward **3-Yb**. (**b**) Stability test of **1-Yb** in 3 mM of the TEAA buffer in the absence of the enzyme at 37 °C, pH 7.1. (**c**) The sulfatase-catalyzed hydrolysis of **1-Yb** (1.46 mM in a 3 mM TEAA buffer at 37 °C, pH 7.1) followed by UPLC-MS with a 0.60 mg/mL added enzyme. Hydrolysis reaction and blank stability were performed in triplicate, and error bars represent the standard deviation.

With the complexes **1-M** in hand, we proceeded to test their reactivity in the presence and absence of the sulfatase enzyme (Figure 3). We evaluated whether (1) the sulfate ester is stable, i.e., it does not hydrolyze spontaneously to compound **3-M** in the absence of the enzyme, and (2) whether a sulfatase is able to accept compounds **1-M** as substrates (Figure 3a). We chose **1-Yb** as the model compound and the sulfatase from *Helix pomatia* as the model enzyme, considering that this enzyme has been also used as a model catalyst in the design of a diamagnetic CEST imaging agent by Sinharay et al. [24]. We were delighted to observe that in the absence of the enzyme, the CEST contrast agent **1-Yb** is stable in a buffer, and the *m*/*z* signal corresponding to the product of its hydrolysis and subsequent 1,6-elimination (compound **3-Yb**) could not be detected by UPLC-MS (Figure 3b). Gratifyingly, in the presence of the enzyme (Figure 3c (0.60 mg/mL sulfatase) and Figures S2 and S3 (0.13 and 0.63 mg/mL sulfatase)), the imaging agent underwent hydrolysis to the desired product, and the kinetics of this transformation were dependent on the amount of the enzyme added. Altogether, these results indicate that the designed complexes are hydrolytically stable in the absence of sulfatase and that the enzyme is able to convert them as substrates. 

### 2.3. A Fast Field Cycling NMR Relaxometry Analysis of MRI Contrast Agent 1-Gd

**Figure 4:**
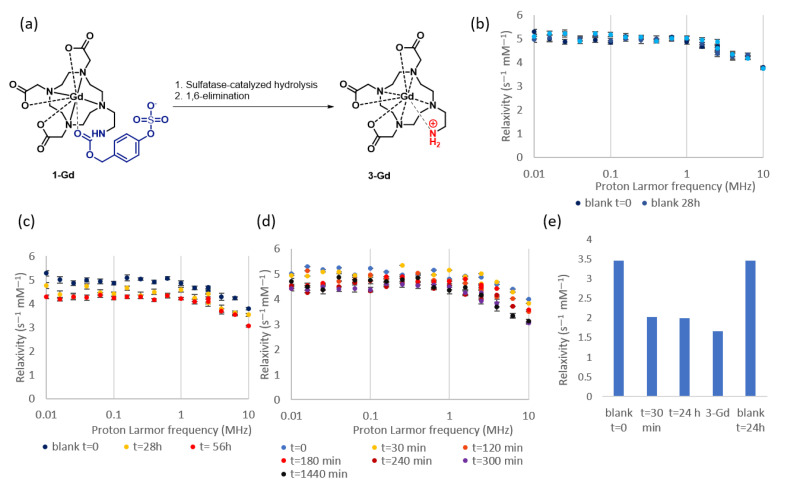
NMRD relaxometric analysis of **1-Gd** (1.0 mM) in the TEAA buffer (3 mM) at 37 °C, pH 7.35. Error bars represent the uncertainty of fitting the T_1_ curve to the experimental data. (**a**) The sulfatase catalyzed hydrolysis of **1-Gd** toward **3-Gd**. (**b**) NMRD profile of **1-Gd** without any enzyme over 56 h. (**c**) NMRD profile of **1-Gd** with 0.086 mg/mL of an enzyme over 56 h. (**d**) NMRD profile of **1-Gd** with more enzymes added (0.46 mg/mL). (**e**) The relaxivity of **1-Gd** with and without the sulfatase enzyme at time points t = 0, t = 30 min, and t = 24 h, blank stability after 24 h, and the relaxivity of independently synthesized **3-Gd** at 4.7T.

Having confirmed the expected behavior of the complexes involving ligand **1** in the presence of the sulfatase, we proceeded to evaluate the performance of **1-Gd** in terms of relaxation enhancement and explore its potential as an enzyme-responsive MRI contrast agent. For this purpose, we collected a nuclear magnetic relaxation dispersion profile (NMRD) using Fast Field Cycling (FFC) relaxometry. With this method, the relaxivity (r_1_ = 1/T_1_) of **1-Gd** was measured over a range of magnetic fields to gain insights into its magnetic properties as a contrast agent [32]. The NMRD profile of compound **1-Gd** is presented in Figure 4a, showing a typical shape for small molecule contrast agents, with a plateau at low fields and a drop in relaxivity in the 1–10 MHz range [33]. In line with the HPLC results on the compound’s stability (see Section 2.2), we did not observe major changes in the profile upon the incubation of the buffered solution for up to 56 h at 37 °C (Figure 4a). In the presence of the enzyme, however, we observed an overall decrease in relaxivity across the entire studied range of magnetic fields (Figure 4b). At 10 MHz, the total decrease in relaxivity from 4.0 s^−1^ mM^−1^ to 3.01 ^−1^ mM^−1^ was measured, giving a 25% change in total. At higher concentrations of the enzyme (Figure 4c), a change of 12.5% was observed after 120 min of enzymatic reaction. Notably, an independently prepared solution of the model reaction product (compound **3-Gd**) at the same concentration showed a relaxivity of 2.6 s^−1^ mM^−1^. Importantly, the relaxivity of compound **3-Gd** has been found before to be strongly dependent on the protonation state of the amine, and the pK_a_ was estimated to be 5.95 [27]. Since we have measured the pH of the solution to be around 1.4 units higher and to remain fairly constant (7.35 prior and 7.31 after the enzymatic conversion), we exclude the possibility that the change in relaxivity is caused by pH and not by the change in the structure of the molecule.

We then proceed to measure the relaxivity at a higher field (4.7T, Figure 4e (see Supplementary Materials Section S4, Table S9), which is more representative of the magnetic field strength used in (pre)clinical applications, i.e., 1.5-7T), where, to our delight, we observed an even more pronounced difference in relaxivity when we measured the **1-Gd** and **3-Gd** independently (52%). Upon the addition of the enzyme to the solution of **1-Gd**, this difference could be to a large extent reproduced (42%). To exclude the possibility that the difference stems from the liberation of the lanthanide ion from the complex, we performed a colorimetric assay to assess the free Gd^3+^ ions in the solution before and after enzymatic hydrolysis. The assay confirmed that there is no significant release of Gd^3+^ into the solution (see Supplementary Materials Section S6, Table S10).

Furthermore, to confirm the stability of **1-Gd** in more biologically relevant media, we incubated the solution of **1-Gd** in a human plasma-like medium (HPLM) and performed a UPLC-MS analysis before and after 24 h of incubation at 37 °C (see Supplementary Materials Section S7, Table S11 and Figure S18). Gratifyingly, we found that more than 90% of the **1-Gd** was still present in the solution after 24 h incubation.

In addition to the stability of the lanthanide complex in the buffer, we further probed its specificity toward sulfatase among other hydrolytic enzymes by following the NMRD profile changes in time in the presence of esterase from a porcine liver, which can hydrolyze substrates, including those featuring ester, thioester, and amide bonds (see Supplementary Materials Figure S3). We see no decrease in relaxivity over time in the presence of this esterase, which was also supported by UPLC-MS analysis (Supplementary Materials Figures S11 and S12). We, therefore, conclude that compound **1-Gd** shows at least a certain level of selectivity toward enzymes with sulfatase activity (see also Supplementary Materials Figure S7).

### 2.4. Z-Spectra Activity Analysis of 1-Yb for CEST Applications

**Figure 5:**
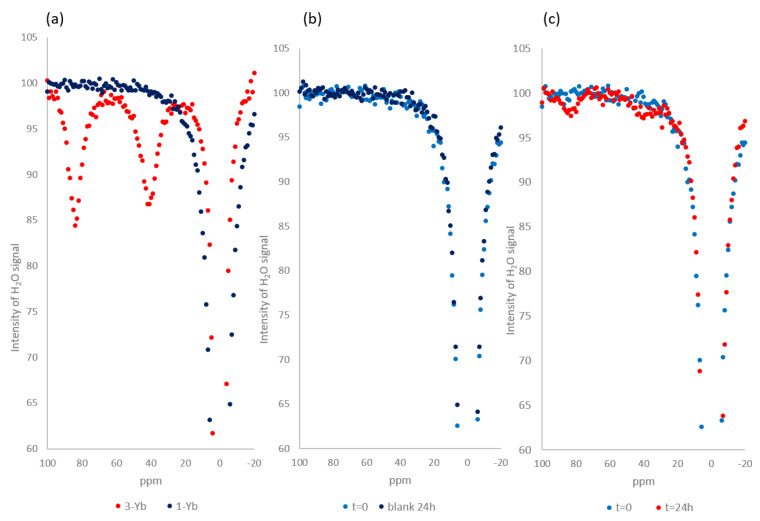
The NMR-Z profiles for **1-Yb** and **3-Yb**. (**a**) Z-spectra of **1-Yb** overlayed with **3-Yb** (20 mM in water with 10% D_2_O, pH adjusted to 7.4 with 1 mM LiOH, B0 = 11.7 T, satpwr = 28 dB, satdly = 2 s) at T = 37 °C. (**b**) Z-spectra of the solution of **1-Yb** at t = 0 and t = 24 h incubation at 37 °C (20 mM in a 3 mM TEAA buffer with 10% D_2_O, pH 7.40, B0 = 11.7 T, satpwr = 28 dB, satdly = 2 s). (**c**) Z-spectra of the enzymatic reaction of **1-Yb** at t = 0 and t = 24 h incubation at 37 °C (20 mM in a 3 mM TEAA buffer with 10% D_2_O, pH 7.4, a 0.33 mg/mL enzyme added, B0 = 11.7 T, satpwr = 28 dB, satdly = 2 s). The complete spectra are presented in the Supporting Information file (Section S5, Figures S13–S15).

We next assessed the performance of **1-Yb** as a CEST imaging agent using NMR to collect Z-spectra. During the collection of a Z-spectrum, a specific radiofrequency is applied every 1 ppm over a wide range of Larmor frequencies (typically between −100 and +100 ppm). A comparison of NMR-Z spectra of **1-Yb** and the independently prepared product of its hydrolysis **3-Yb** (Figure 5a) confirmed our hypothesis that the unactivated compound is CEST-inactive, as it does not show any signals in the 20–100 ppm region, while the independently synthesized **3-Yb** shows two signals at ~40 and ~90 ppm, which originate from the protons of the amine, as indicated by Krchová et al. [28]. The same analytical method (Figure 5b) confirmed that in the absence of the enzyme, compound **1-Yb** is hydrolytically stable, as no new signals were observed after 24 h of incubation. To our delight, the addition of the sulfatase (0.33 mg/mL) to **1-Yb** resulted in the formation of the expected two signals, showing the potential of compound **1-Yb** as a “switch-on” CEST imaging agent for the detection of sulfatase activity. The respective Z-spectrum (Figure 5b) clearly shows that prior to adding the enzyme to the contrast agent, no CEST signal is generated, whereas, after the addition of the enzyme (0.33 mg/mL, Figure 5c), two peaks are formed (42 ppm and 89 ppm). Although the signals emerging in Figure 5c do not have the same intensity as those obtained with pure compound **3-Yb** (Figure 5a), this is a result of incomplete enzymatic conversion (as confirmed by LCMS analysis, see Supplementary Materials Section S5, Figure S16) due to the fact that the CEST experiments are performed at 20 mM concentrations of substrate, while the concentration enzyme cannot be matched due to solubility issues.

Afterward, we used a colorimetric assay to assess the free Yb^3+^ ions in the solution, before and after enzymatic hydrolysis. The assay confirmed that there was no significant release of Yb^3+^ into the solution (see Supplementary Materials Section S6, Table S10).

## 3. Discussion

We designed, synthesized, and evaluated a sulfatase-responsive MRI contrast agent **1-Gd** and paraCEST imaging agent **1-Yb**, which are both stable in an aqueous solution and showed no hydrolysis without the sulfatase enzyme present. Upon the sulfatase-catalyzed hydrolysis of **1-Gd**, followed by a spontaneous 1,6-elimination of the self-immolating linker, **3-Gd** is formed, which shows a 25% difference in relaxivity at a 0.23 T magnetic field (corresponding to a 10 mHz Larmor frequency). Our results are comparable with previous work from our group by Reeβing et al. on light-responsive contrast agents [34], which upon activation, showed a decrease in relaxivity of 17%, while the difference was even larger when the photo-responsive contrast agents that were measured on a clinical 1.5T and 3.0T MRI system. In the case of the compounds presented, at a higher magnetic field (4.7T, representative for the (pre)clinical 1.5T–7T systems), we observed a 42% drop in relaxivity upon enzymatic activation. The difference in relaxivity obtained with our sulfatase-responsive contrast agents is also comparable to other enzyme-responsive contrast agents published in the literature; for example, for the imaging of β-galactosidase activity (20% difference at 11.7T) [20] and β-glucuronidase activity (27% difference at 1.4T) [26].

In our quest for a sulfatase-responsive imaging agent that shows an off-to-on effect, i.e., it is “silent” in the absence of the enzyme and only shows a signal upon enzymatic activation, we next turned to CEST imaging, using Yb^3+^ as the metal for complexation with ligand **1**. Indeed, only after the hydrolysis of **1-Yb** to **3-Yb** by the sulfatase was a saturation transfer to the bulk water observed, which gave rise to two signals (42 ppm and 89 ppm) and shifted far away from the water signal.

The effects observed for both molecules probably originate from the coordination of the amine group, which is liberated in the enzymatic process, to the metal center. For **1-Gd**, the enzymatic activation and liberation of the stronger ligand—the amine group—leads to a change in the coordination number (q) of water molecules, resulting in a decrease in relativity. Analogously, for **1-Yb**, the coordination to the metal center induces a shift in ppm from the bulk water, resulting in peaks at 42 ppm and 89 ppm resonating with the water frequency. This is due to the closer distance of the amine to the metal center and greater alignment with the magnetic axis.

Taken together, the work presented, which is supported by fast field cycling NMR relaxometry and Z-spectroscopy, provides a molecular proof-of-principle for the non-invasive imaging of the activity of key enzymes.

## 4. Materials and Methods

### 4.1. Synthetic Procedures and Spectroscopic Data

Synthesis procedures for all compounds and the characterizations of all compounds are in the Supporting Information.

### 4.2. Enzymatic Hydrolysis

Three enzymatic hydrolyses were carried out. Reaction 1 (shown in Figure 3c) was performed in triplicate, and two initial reactions (enzyme reactions 2 and 3) presented in Supporting Information (Section S3, Tables S3 and S4, and Figure S2–S4) were performed in duplicate. All the enzymatic hydrolysis reactions were analyzed by following the reaction by use of UPLC-MS; see for further details Supporting Information Section S3.

### 4.3. FFC Relaxometry

The relaxation rates were determined over a (proton) Larmor frequency range of 0.01–10 MHz at 37 °C with 17 data points collected. A stock solution of **1**-**Gd** was prepared (1.0 mM in 3 mL of a 3 mM TEAA buffer, pH 7.35) and divided into two samples for the blank and enzymatic reaction. NMRD profiles were recorded before and after adding the sulfatase from *Helix pomatia* (0.086 mg/mL, 1.38 unit) and after 28 h and 56 h (see Supplementary Materials Section S4). In addition, the stability of the blank sample was assessed by repeating the analysis after leaving the sample for 28 h and 56 h at 37 °C without the enzyme. The NMRD profile with 5 times more sulfatase was collected by adding sulfatase from *Helix pomatia* (0.46 mg/mL, 7.42 units) to the blank, and an NMRD profile was recorded at 30, 120, 180, 240, 300, and 1440 min. From both the enzyme and black reactions, the samples were taken for UPLC-MS analysis (see Supplementary Materials). The pH after the enzymatic reaction was 7.31.

The relaxation rates at 4.7T were recorded on a Varian 200 MHz NMR, with an inversion recovery method at 37 °C. A stock solution of **1-Gd** was prepared (1 mM in 1 mL of a 3 mM TEAA buffer consistent with 10% D_2_O, pH 7.35) and divided into two samples: the blank and the enzymatic reaction, to which sulfatase from *Helix pomatia* (0.46 mg/mL, 7.42 units) was added. The solution was then transferred to a 3 mm sample tube (0.25 mL per tube). Every data point was measured in duplicate (see Supplementary Materials Section S4, Table S9).

The stability of compound **1-Gd** in the presence of esterase from the porcine liver was performed in a similar way as described above. A stock solution of **1-Gd** was prepared (1 mM in 3 mL of a 3 mM TEAA buffer, pH 7.34) and divided into two samples: the blank and enzymatic reaction. NMRD profiles were recorded before and after adding the esterase (1.18 mg/mL, 73.8 units). Times points measured were 30, 120, 180, 240, and 1440 min (Supplementary Materials Section S4, Table S8, Figure S6). From both the enzyme and blank reactions, the samples were taken to analyze compounds present via UPLC-MS. (Figures S11 and S12).

The stability of compound **1-Gd** in HPLM was determined by following the samples 24 h at 37 °C with UPLC-MS. Three samples were made of **1-Gd** (1mM in 0.5 mL HPLM), and the samples were analyzed by UPLC-MS at time points t = 0 h and t = 24 h at 37 °C (see Supplementary Materials Section S7, Table S11, Figure S18).

### 4.4. Z-spectra

All Z-spectra were recorded on a Varian Oxford AS 500 MHz (*B*_0_ = 11.7T) using 5 mm sample tubes. A stock solution of **1-Yb** was prepared (20 mM in 1.5 mL of a 3 mM TEAA buffer with 10% D_2_O, pH 7.40) and divided into two samples for the blank and enzymatic reaction. The Z-spectra were recorded before adding the sulfatase from *Helix pomatia* (0.33 mg/mL, 5.32 units) and after 24 h and 48 h (see Supplementary Materials, Section S5). In addition, the stability of the blank was assessed by repeating the analysis after leaving the sample for 24 h and 48h at 37 degrees without the enzyme. The pH after the enzymatic reaction was 7.38. Standard pulse sequences for presaturation experiments were used (see Supplementary Materials Section S5 and Figure S50). Saturation offsets were set using the array function (increment 200–250 Hz). The parameters of the presaturation pulse were B0 = 11.7 T, satpwr = 28 dB, and satdly = 2 s.

## 5. Conclusions

In this manuscript, we present the proof of principle for the first sulfatase-responsive MRI contrast agent that features a sulfate attached to a Gd3^+^ complex through a self-immolating linker. Upon enzymatic activation, a decrease in relaxivity was observed (25% at 0.23T, 42% at 4.7T), making it a turn-off agent. Conversely, an off–on activation of the signal could be obtained with the CEST effect when the Yb^3+^ complex was used. Future research will focus on the engineering of the self-immolating linker, which could lead to compounds that show more efficient activation [21] of the pre-clinical aspects of the molecules presented, including the ADME profile, tumor accumulation, and toxicity.

## Data Availability

Data is contained within the article and the supplementary materials. The raw data are available on request from the corresponding author.

## Supplementary Materials

The following supporting information can be downloaded at https://www.mdpi.com/article/10.3390/ph16101439/s1. References used in supplementary materials: 28, 30, 31, 35 and 36. Synthetic procedures and NMR spectroscopic data for all new compounds, Figure S1: Synthetic overview route toward compound **1-Gd** and **1-Yb** and the model products of their enzymatic hydrolysis, compounds **3-Gd** and **3-Yb**. Figure S2. The sulfatase-catalyzed hydrolysis of **1-Yb** (1.46 mM in a 3 mM TEAA buffer at 37 °C, pH 7.1) followed by UPLC-MS with 0.13 mg/mL of an added enzyme (enzyme reaction 2). A hydrolysis reaction was performed in duplicate (reaction 2-1: substrate shown in light blue product formation in light red; reaction 2–2: substrate shown in dark blue, product formation in dark red). Figure S3. The sulfatase-catalyzed hydrolysis of **1-Yb** (1.46 mM in a 3 mM TEAA buffer at 37 °C, pH 7.1) followed by UPLC-MS with 0.63 mg/mL of an added enzyme (enzyme reaction 3). A hydrolysis reaction was performed in duplicate (reaction 3–1: substrate shown in light blue product formation in light red; reaction 3–2: substrate shown in dark blue, product formation in dark red). Figure S4. Stability test of **1-Yb** in 3 mM of a TEAA buffer in the absence of the enzyme at 37 °C, pH 7.1. Figure S5. NMRD relaxometric analysis of **1-Gd** (1.0 mM) in a TEAA buffer (3 mM) at 37 °C with the addition of 0.086 mg/mL of an enzyme followed over 56 h, including **3-Gd**. Error bars represent the uncertainty of fitting the T_1_ curve to the experimental data. Figure S6. NMRD relaxometric analysis of **1-Gd** (1.0 mM) in a TEAA buffer (3 mM) at 37 °C with the addition of 1.18 mg/mL of an esterase from a porcine liver followed over 24 h. Error bars represent the uncertainty of fitting the T1 curve to the experimental data. Figure S7. NMRD relaxometric analysis of **1-Gd** (1.0 mM) in a TEAA buffer (3 mM) at 37 °C with the addition of 1.18 mg/mL of an esterase from a porcine liver followed over 24 h. vs. the NMRD relaxometric analysis of **1-Gd** (1.0 mM) in a TEAA buffer (3 mM) at 37 °C with the addition of 0.46 mg/mL of a sulfatase followed over 24 h. Error bars represent the uncertainty of fitting the T_1_ curve to the experimental data. Figure S8. UPLC-MS analysis of blank **1-Gd** before adding sulfatase. (a) Total ion current chromatogram negative mode, (b) total ion current chromatogram positive mode, (c) 770–774 mass trace, (d) mass spectrum of the peak at Rt = 1.32 min. Figure S9. UPLC-MS analysis of sulfatase hydrolysis reaction (0.086 mg/mL) with **1-Gd** t = 28h. (a) Total ion current chromatogram negative mode, (b) total ion current chromatogram positive mode, (c) 770–774 mass trace, (d) 540–544 mass trace, (e) mass spectrum of the peak at Rt = 1.31 min, (f) mass spectrum of the peak at Rt = 0.97 min. Figure S10. UPLC-MS analysis of the sulfatase hydrolysis (0.49 mg/mL) of **1-Gd** t = 300 min. (a) Total ion current chromatogram negative mode, (b) total ion current chromatogram positive mode, (c) 770-774 mass trace, (d) 540–544 mass trace, (e) mass spectrum of the peak at Rt = 0.98 min, (f) mass spectrum of the peak at Rt = 1.41 min. Figure S11. UPLC-MS analysis of a blank **1-Gd** before adding esterase. (a) Total ion current chromatogram negative mode, (b) total ion current chromatogram positive mode, (c) 770–774 mass trace, (d) 544–545 mass trace, (e) mass spectrum of the peak at Rt = 1.43 min. Figure S12. UPLC-MS analysis of **1-Gd**, 24 h (1440 min) after adding esterase. (a) Total ion current chromatogram negative mode, (b) total ion current chromatogram positive mode, (c) 770–774 mass trace, (d) 544–545 mass trace, (e) mass spectrum of the peak at Rt = 1.67 min. Figure S13. Z-spectra of **1-Yb** overlapped on **3-Yb** (20 mM in water with 10% D_2_O, pH adjusted to 7.4 with 1 mM LiOH, B0 = 11.7 T, satpwr = 28 dB, satdly = 2 s) at T = 37 °C. Figure S14. Z-spectra of the solution of **1-Yb** at t = 0 and t = 24 h incubation at 37 °C (20 mM in a 3 mM TEAA buffer with 10% D_2_O, pH 7.40, B0 = 11.7 T, satpwr = 28 dB, satdly = 2 s). Figure S15. Z-spectra of the enzymatic reaction of **1-Yb** at t = 0 and t=24 h incubation at 37 °C (20 mM in a 3 mM TEAA buffer with 10% D_2_O, pH 7.4, 0.33 mg/mL sulfatase. Figure S16. UPLC-MS analysis of enzyme reaction with **1-Yb** t = 24 h. (a) Total ion current chromatogram negative mode, (b) total ion current chromatogram positive mode), (c) 778-790 mass trace, (d) 560-562 mass trace, (e) mass spectrum of the peak at Rt = 0.98 min, (f) mass spectrum of the peak at Rt = 1.36 min. Figure S17. Quantification of free YbIII and GdIII. (a) Calibration curve showing the ratio of absorbance intensity at λ = 573 nm and λ = 433 nm for increasing YbIII concentration in the presence of xylenol orange (0.60 mM). (b) Calibration curve showing the ratio of absorbance intensity at λ = 573 nm and λ = 433 nm for increasing GdIII concentration in the presence of xylenol orange (0.60 mM). Figure S18. UPLC-MS analysis of **1-Gd** (1.0 mM) in HPLM at 37 °C followed over 24 h. Error bars represent the standard deviation. Figures S19–S28. NMR analysis of compounds **4**, **5**, **9**, **6**, **8**, **1-Yb**, **1-Gd**, **3**, **3-Gd**, **3-Yb**. Figures S29–S38. HRSM analysis of compounds **9**, **6**, **7**, **8**, **1**, **1-Yb**, **1-Gd**, **3**, **3-Gd**, **3-Yb**. Figures S39–S49. LCMS analysis of compounds **6**, **9**, **10**, **7**, **8**, **1**, **1-Yb**, **1-Gd**, **3**, **3-Yb**, **3-Gd**. Figure S50. Pulse sequence used to measure the Z-spectra. Table S1. LCMS analysis of sulfatase enzyme reaction 1 of **1-Yb** in a TEAA buffer, pH 7.1. Peak areas of **1-Yb** and **3-Yb** for every run. In the time intervals 0, 15, 30, 60, 120, 180, 240 min. Table S2. LCMS analysis of the blanks of enzyme reaction 1 of **1-Yb** in a TEAA buffer, pH 7.1. Peak areas of **1-Yb** for every run. In the time intervals 0, 15, 30, 60, 120, 180, 240 min. (triplicate). Table S3. LCMS analysis of sulfatase enzyme reaction 2 of **1-Yb** in a TEAA buffer, pH 7.1. Peak areas of **1-Yb** and **3-Yb** for every run. In the time intervals 1, 5, 15, 30, 60, 120, 180, 240, 420, 1080 min. Table S4. LCMS analysis of sulfatase enzyme reaction 3 of **1-Yb** in a TEAA buffer, pH 7.1. Peak areas of **1-Yb** and **3-Yb** for every run. In the time intervals 0, 3, 5, 15, 30, 60, 120, 180, 240, and 420 min. Table S5. LCMS analysis of the blank of **1-Yb** in a TEAA buffer, pH 7.1. Peak areas of **1-Yb** and **3-Yb** for every run. In the time intervals 15, 30, 60, 120, 180, 420, 1080 min. Table S6. Molar relaxivity (s-1 mM-1) profiles of a sample of **1-Gd** in a TEAA buffer, pH 7.35. Blank t=0, blank 28 h, blank 56h. Enzyme reaction t = 0, enzyme reaction t = 28 h, and enzyme reaction t = 56 h (sulfatase 0.086 mg/mL). Table S7. Molar relaxivity (s-1 mM-1) profiles of a sample of **1-Gd** in a TEAA buffer pH 7.35, time points t = 0, 30, 120, 180, 240, 300, and 1440 min. (sulfatase 0.46 mg/mL). Table S8. Molar relaxivity (s-1 mM-1) profiles of a sample of **1-Gd** in a TEAA buffer, pH 7.34, time points t = 0, 30, 120, 180, 240, 300, and 1440 min. (esterase 1.18 mg/mL). Table S9. Molar relaxivity (s-1 mM-1) profiles of a sample of **1-Gd** in a TEAA buffer pH 7.35 at 4.7 T. Blank t = 0, blank 24 h, enzyme (sulfatase) reaction t= 30, 50 min, 60 min, 120 min, 180 min, 24 h. All points are measured in duplicate. Table S10. Results of the determination of free metal concentration. Column 1: sample name, column 2: ratio of absorbance intensity at λ = 573 nm and λ = 433 nm. Column 3: calculated free lanthanide concentration in μM. Column 4: recalculated concentration of free metal taking into account the dilution of the sample. Column 5: % of free lanthanide in the sample. Table S11. LCMS analysis of **1-Gd** in HPLM. Peak areas of **1-Yb** for time point t = 0 and t = 24 h at 37 °C. Refs. [35,36] has been cited in supplementary materials.
