# Supplementary material for: The Development of a Smart Magnetic Resonance Imaging and Chemical Exchange Saturation Transfer Contrast Agent for the Imaging of Sulfatase Activity
Source: Pharmaceuticals (Basel). 2023 Oct 11;16(10):1439. doi: 10.3390/ph16101439 (PMC10610007; doi:10.3390/ph16101439)
Supplement: Supplementary file 1 [file pharmaceuticals-16-01439-s001.zip › pharmaceuticals-2581028-supplementary.pdf]

Supplementary Materials

Development of a smart MRI and CEST contrast agent for the imaging of sulfatase activity

**Ilse M. Welleman [1,2], Friederike Reeβing [1,2], Hendrikus H. Boersma [1,3], Rudi A. J. O. Dierckx [1], Ben L. Feringa [2]
and Wiktor Szymanski [1,2,*,†]**

[1] Department of Radiology, Medical Imaging Center, University Medical Center Groningen, University of Groningen, Hanzeplein 1, 9713 GZ Groningen, The Netherlands; i.m.welleman@umcg.nl (I.M.W.); f.reesing@umcg.nl (F.R.); h.h.boersma@umcg.nl (H.H.B.); r.a.dierckx@umcg.nl (R.A.J.O.D.)

[2] Stratingh Institute for Chemistry, University of Groningen, Nijenborgh 4, 9747 AG Groningen, The Netherlands; b.l.feringa@rug.nl

[3] Department of Clinical Pharmacy and Pharmacology, Nuclear Medicine and Molecular Imaging, University Medical Center Groningen, University of Groningen, Hanzeplein 1, 9713 GZ Groningen, The Netherlands

* Correspondence: w.szymanski@umcg.nl

† Current address: Groningen Research Institute of Pharmacy, Department of Medicinal Chemistry, Photopharmacology and Imaging, University of Groningen, Antonius Deusinglaan 1, 9713 AV Groningen, The Netherlands.

1. General information

All solvents used for extraction, filtration and chromatography were of commercial grade, and used without further purification. Reagents were purchased from Sigma- Aldrich, TCI, BOOM and Combi Blocks and were used without further purification. For purification with column chromatography silica gel from Macherey-Nagel (Silica 60 M, 0.04-0.063 nm, 230-400 mesh) was used.

The TLC analysis were performed on Merck silica gel 60, 0.25 mm plates and stained with potassium permanganate stain (a mixture of $KMnO_4$ (3 g), K_2CO_3 (10 g), water (300 mL)) or Seebach's stain (a mixture of phosphomolybdic acid (2.5 g), $Ce(SO_4)_2$ (1 g), H_2SO_4 (con 6 ml) and water 94 ml) or were visualized under a UV-lamp (λ = 254 nm).

NMR spectra were obtained using Agilent Technologies 400 MR (400/54 Premium Shielded) (^{1}H: 400 MHz, ^{13}C: 101 MHz) using $CDCl_3$, $(CD_3)_2SO$ or D_2O as solvent. Chemical shift values are reported in ppm with the solvent resonance as the internal standard ($CDCl_3$: δ 7.26 for ^{1}H, δ 77.16 for ^{13}C, $(CD_3)_2SO$: δ 2.50 for ^{1}H and δ 39.52 for ^{13}C and D_2O: δ 4.79 for ^{1}H). Data are reported as follows: chemical shifts (δ), multiplicity (s = singlet, d = doublet, t = triplet, q =quartet, br = broad, m = multiplet), coupling constant J (Hz), integration.

High Resolution Mass measurements were performed using a Thermo Scientific LTQ OribitrapXL spectrometer.

CH-100 BIOSAN dry block Heating/Cooling thermostat was used as heating device during the enzymatic cleavage of sulfate group.

The determination of free Yb^{3+} and Gd^{3+} concentration was performed using a Synergy H1 microplate reader.

NMRDs were recorded on a Stelar 0.25T FFC SMARtracer relaxometer (Mede, Italy).

Relaxivity at 4.7 Telsa was recorded on a Varian Oxford 200 MHz.

UPLC–MS analysis was performed using a ThermoFisher Scientific Vanquish UPLC System (Waltham, MA, USA) with a reversed phase C18 column (Acquity UPLC BEH C18 (1.7 μm, 2.1 150 mm) in combination with an LCQ Fleet mass spectrometer and UV–vis detector at 254 nm. Using Program 1: Eluent A: acetonitrile Eluent B: water, both with 0.1% v/v formic acid added. Program 0-2 min 5% of eluent A, from 2-10 gradient 5-90% eluent A, 10-12 min flush of 100% A, 12-18 min going back to 5% eluent A. Or on Acquity UPLC-MS with TQD fitted with a BEH C18 column (1.7 uM 2.1 x 50 mm). Using Program 2: Eluent A: acetonitrile Eluent B: water, both with 0.1% v/v formic acid added. Program 0-2 min 5% of eluent A, from 2-10 gradient 5-90% eluent A, 10-15 min flush of 100% A, 15-20 min going back to 5% A.
Program 3: Eluent A: acetonitrile Eluent B: water, both with 0.1% v/v formic acid added. Program 0-2 min 2% of eluent A, from 2-10 gradient 2-90% eluent A, 10-15 min flush of 100% A, 15-20 min going back to 2% A

Enzymatic analysis was performed on a Thermo Scientific vanquish UHPLC with ACQUITY UPLC BEH C8, 2.1mm x 150 mm 1.7μm particles column and Thermo Scientific LCQ fleet ion trap as mass detector. Program 4: Eluent A: acetonitrile Eluent B: water, both with 0.1% v/v formic acid added. Program 0-3 min 2% of eluent A, from 3-7 gradient 2-90% eluent A, 7-12 min flush of 100% A, 12-15 min going back to 2% A.

NMR-Z spectrums were recorded on Varian Oxford AS 500 MHz.

Preparative HPLC purification was performed on a Shimadzu HPLC system with a Phenomenex® Kinetex 5 μm EVO C18 100 Å column, using Program 3: Eluent A: acetonitrile Eluent B: 10 mM triethylammonium acetate in water. Program: 0-20 min: gradient 2-25% eluent A, 20-30 min 100 % eluent A, 30-36 min 2% A.

The pH measurements were performed on a Mettler Toledo FiveEasy pH meter

2. Synthetic procedures and spectroscopic data

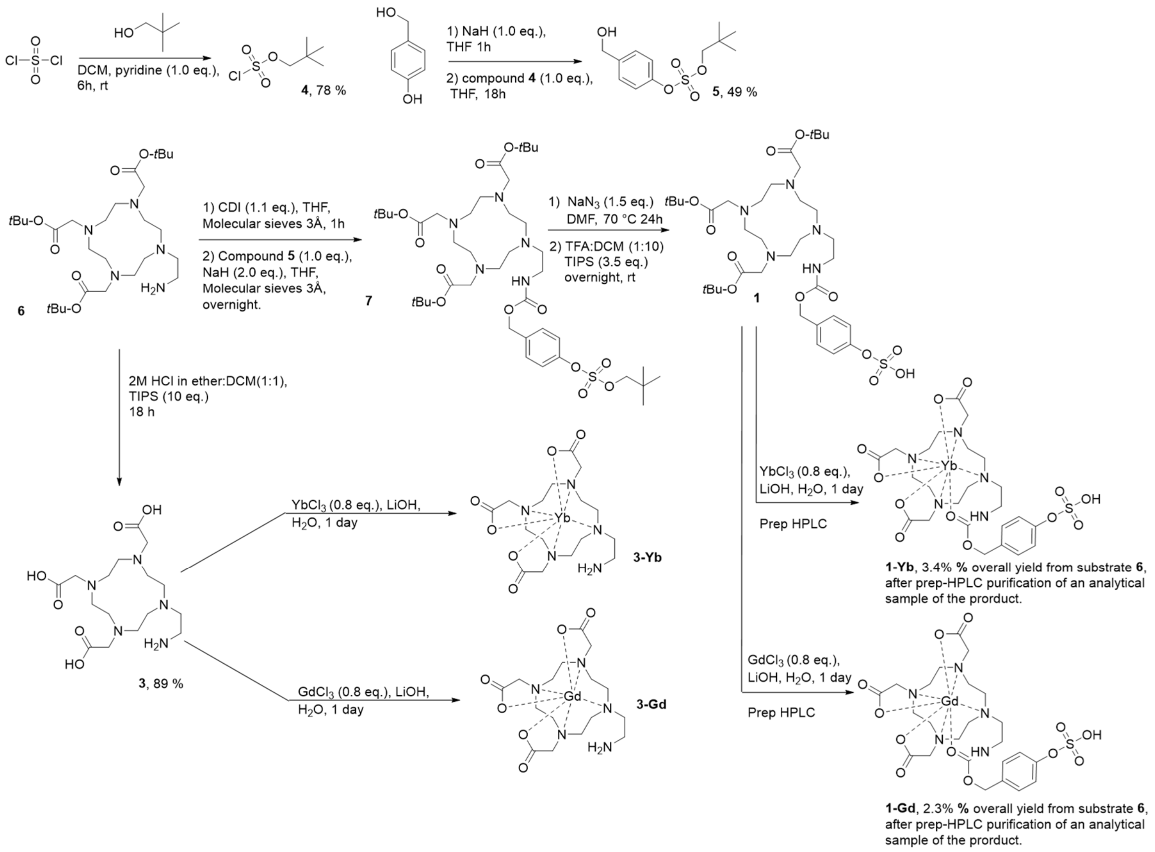

Figure S1. Synthetic overview of the route towards compound **1-Gd** and **1-Yb** and the model products of their enzymatic hydrolysis, compounds **3-Gd** and **3-Yb**.

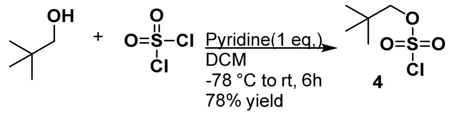

4: Neopentyl chlorosulfate: Prepared by a modification of a literature procedure[1]. A solution of sulfuryl chloride (4.30 mL, 53.0 mmol, 1.00 eq.) in anhydrous DCM (20 mL) was cooled down to -78 °C under nitrogen atmosphere. Then, a solution of neopentyl alcohol (4.70 g, 53.0 mmol, 1.00 eq.) and dry pyridine (4.30 mL, 53.0 mmol, 1.00 eq.) in anhydrous DCM (20 mL) was added dropwise via a dropping funnel, over a period of 30 min. The resulting colorless solution was allowed to warm up to room temperature and was stirred under nitrogen atmosphere for 6 h. Upon completion, the crude reaction mixture was filtered to remove the precipitated pyridinium salts and the filtrate was concentrated under reduced pressure. (Temperature of the water bath should not surpass 30 °C) The crude product (clear liquid) was purified by a filtration over silica (DCM as solvent) affording the desired pure neopentyl chlorosulfate **4** as a colorless liquid with a strong odor. Isolated yield 78% (7.74 g, 41.5 mmol). Reaction was performed 5 times with yields ranging from 55-78%, compound **4** was stored for max 1 day at room temperature in a closed vial with glass lit.
^{1}H NMR (400 MHz, Chloroform-*d*) δ 4.17 (s, 2H), 1.05 (s, 9H).^{13}C NMR (101 MHz, Chloroform-*d*) δ 85.5, 31.9, 25.9. NMR spectra in agreement with the literature data.[1]

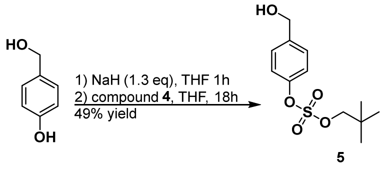

5: 4-(Hydroxymethyl)phenyl neopentyl sulfate:

A dried three-neck flask was put under nitrogen atmosphere and was charged with NaH (129 mg, 5.30 mmol, 1.10 eq.) and 20 mL of dry THF. The flask was cooled with an ice bath and 4-hydroxybenzyl alcohol (600 mg, 4.88 mmol, 1.00 eq.) was introduced in portions. After stirring the reaction mixture at room temperature for 1 h, a solution of neopentyl chlorosulfate **4** (1.00 g, 5.30 mmol, 1.10 eq.) in anhydrous THF (10 mL) was added dropwise via a syringe. After the addition, the resulting reaction mixture underwent color changes from pastel pink/purple to yellow to brown. The resulting brown solution was stirred at room temperature for 18 h. Upon completion (TLC), the solids were filtered off, and the solvent was evaporated. The crude residue was purified by recrystallization in DCM, affording the desired compound **5** as a brown solid. Isolated yield 49% (700 mg, 2.55 mmol). Reaction was performed 5 times with yields ranging from 47-54%, compound **5** was stored for max 7 days at room temperature in a closed vial.

R_f = 0.65 (pentane/AcOEt, 3:2, v/v); Mp. 72-74 °C; ^{1}H NMR (400 MHz, Chloroform-*d*) δ 7.42 (d, *J* = 8.6 Hz, 2H), 7.30 (d, *J* = 8.6 Hz, 2H), 4.71 (s, 2H), 4.09 (s, 2H), 1.00 (s, 9H) ppm. ^{13}C NMR (101 MHz, Chloroform-*d*) δ 149.6, 140.1, 128.3, 121.1, 83.5, 64.4, 31.9, 25.9 ppm. NMR spectra in agreement with the literature data.[1]

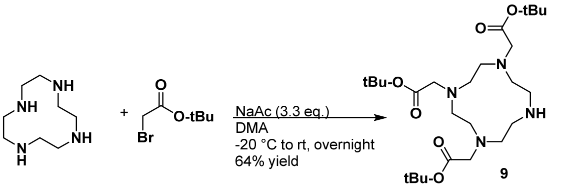

9: Tri-*tert*-butyl 2,2',2''-(1,4,7,10-tetraazacyclododecane-1,4,7-triyl)triacetate: Prepared via a literature procedure.[2] A solution of cyclen (1.00 g, 5.81 mmol, 1.00 eq.) and sodium acetate (1.57 g, 19.1 mmol, 3.30 eq.) in dimethylacetamide (DMA) (12 mL) was cooled down to -20 °C. Then, a solution of *tert*-butyl bromoacetate (3.71 g, 19.1 mmol, 3.30 eq.) in DMA (4 mL) was added dropwise. The resulting white suspension was allowed to warm up to room temperature and was stirred overnight. Upon completion of the reaction (LCMS), the crude reaction mixture was poured into water (65 mL). To the resulting colorless solution, potassium bicarbonate (3.00 g, 30.0 mmol) was added in portions. The precipitate was collected by filtration and dissolved in chloroform (50 mL). The solution was washed with water and dried with magnesium sulfate. The filtrate was concentrated under reduced pressure. The crude residue was purified by recrystallization with ether (50 mL) affording the desired pure compound **9** as a white solid. Isolated yield 64% (2.27 g, 3.80 mmol). Reaction was performed 5 times with yields ranging from 58-68%, compound **9** was stored at room temperature in a closed vial. R_f = 0.68 (DCM/MeOH, 9:1, v/v); Mp. 176-178 °C; ^{1}H NMR (400 MHz, Chloroform-*d*) δ 10.18 (s, 2H), 3.32 (s, 4H), 3.24 (s, 2H), 3.04 (t, *J* = 4.8 Hz, 4H), 2.91 – 2.78 (m, 12H), 1.40 (d, *J* = 3.1 Hz, 27H) ppm.^{13}C NMR (101 MHz, Chloroform-*d*) δ 170.5, 169.6, 81.7, 81.56, 58.2, 51.3, 49.2, 47.5, 28.2, 28.1 ppm. NMR spectra in agreement with the literature data.[2] HRMS (ESI+) calc. for $[M+H]^+$ ($C_{26}H_{51}N_4O_6$): 515.3803, found: 515.3801

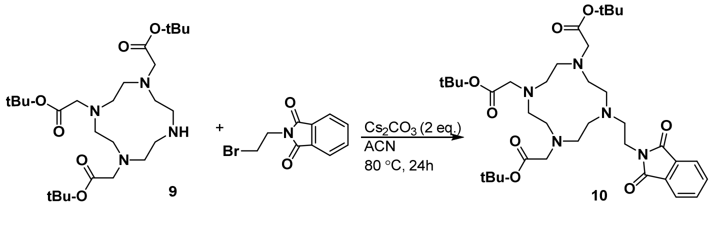

10: Tri-*tert*-butyl 2,2',2''-(10-(2-(1,3-dioxoisoindolin-2-yl)ethyl)-1,4,7,10-tetraazacyclodode-cane-1,4,7-triyl)triacetate: Prepared via a literature procedure.[3] Compound **9** (653 mg, 1.10 mmol, 1.00 eq.) was dissolved in $MgSO_4$ dried ACN (10 mL) under nitrogen atmosphere. *N*-(2-Bromoethyl)phthalimide (653 mg, 1.1 mmol, 1.0 eq.) and cesium carbonate (720 mg, 2.20 mmol, 2.00 eq.) were added. The suspension was heated to 80 °C and stirred for 24 h. Upon reaction completion (LCMS), the crude reaction mixture was filtered. The yellow filtrate was evaporated under reduced pressure and chloroform (20 mL) was added. The impurities where filtered off and the solvent was evaporated, affording the desired crude

compound **10** as a yellow oil. R_f = 0.65 (DCM/MeOH, 9:1, v/v); ^{1}H-NMR (400 MHz, $CDCl_3$): δ 7.82-7.84 (m, 2H), 7.68–7.71 (m, 2H), 3.74-3.77 (t, J=6.7Hz, 2H), 3.21 (s, 6H), 2.69-2.79 (m, 18H), 1.44 (s, 27H) ppm. Compound **10** was used in the next step without further purification.

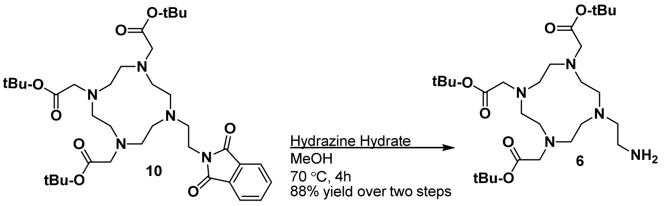

6: Tri-*tert*-butyl 2,2',2''-(10-(2-aminoethyl)-1,4,7,10-tetraazacyclododecane-1,4,7-triyl)triacetate: Prepared via a literature procedure.[3] The crude compound **10** was dissolved in methanol (14 mL) and hydrazine hydrate (reagent grade 50-60%, 120 μL) was added while stirring. The reaction mixture was heated to 70 °C and stirred for 4 h. Upon reaction completion (LCMS), the solvent was evaporated. DCM (20 mL) was added and the resulting solids were filtered out. The filtrate was washed first with distilled water (three times, 30 mL) and then with an aqueous solution of KOH (20%, 20 mL). The organic layer was dried with magnesium sulfate. Evaporation of the solvent under reduced pressure yielded compound **6** as an amber oil (465 mg, 88% over two steps from compound **9**). Reaction was performed 4 times with yields ranging from 72-88%, compound **6** was stored at room temperature in a closed vial. Rf = 0.64 (DCM/MeOH, 9:1, v/v) ^{1}H NMR (400 MHz, Chloroform-d) *δ* 3.45 – 3.24 (m, 6H), 3.13 – 2.33 (m, 20H), 1.42 (s, 27H).^{13}C NMR (101 MHz, Chloroform-d) *δ* 171.5, 170.6, 81.6, 81.1, 56.5, 56.1, 51.6, 50.5, 50.0, 49.6, 46.2, 37.5, 28.2, 28.1. NMR spectra in agreement with the literature data. [3] HRMS (ESI+) calc. for $[M+H]^+$ ($C_{28}H_{56}N_5O_6^+$): 558.4225, found: 558.4219.

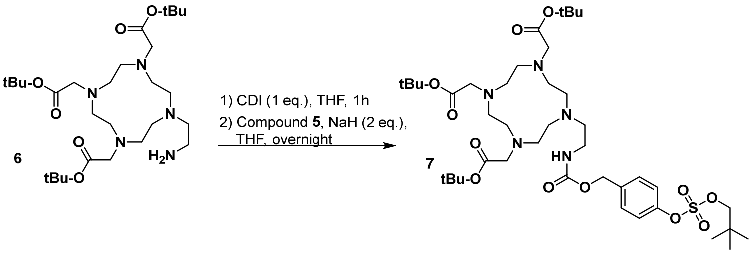

7: Tri-*tert*-butyl 2,2',2''-(10-(2-((((4-((neopentyloxy)sulfonyl)oxy)benzyl)oxy)carbonyl)-amino)ethyl)-1,4,7,10-tetraazacyclododecane-1,4,7-triyl)triacetate:

A three necked flask was put under nitrogen atmosphere and charged with molecular sieves (3 Å beads, 4-8 mesh). A solution of compound **6** (800 mg, 1.44 mmol, 1.00 eq.) in dry THF (5 mL) was added. While stirring, a solution of 1,1'-carbonyldiimidazole (302 mg, 1.80 mmol, 1.30 eq) in dry THF (5 mL) was added, and the reaction mixture was stirred and followed by LCMS. Upon 80% conversion a solution of compound **5** (600 mg, 2.16 mmol, 1.50 eq) and NaH (100 mg, 4.32 mmol 2.00 eq) in 2 mL dry THF was added. The reaction was followed by LCMS. After completion, the reaction mixture was filtered and the solvent was evaporated to obtain crude **7** as dark amber oil. ^{1}H NMR (400 MHz, $CDCl_3$) δ: 7.51-7.49 (d, 2H), 7.41-7.40 (d, 2H), 5.07 (s, 2H), 4.10 (s, 2H), 3.28– 3.33 (two signals overlapping, 6H), 2.58–2.84 (m, 20H), 1.44-1,46 (s, 27H), 1.01 (s, 9H) ppm. Compound **7** was used in the next step without further purification.

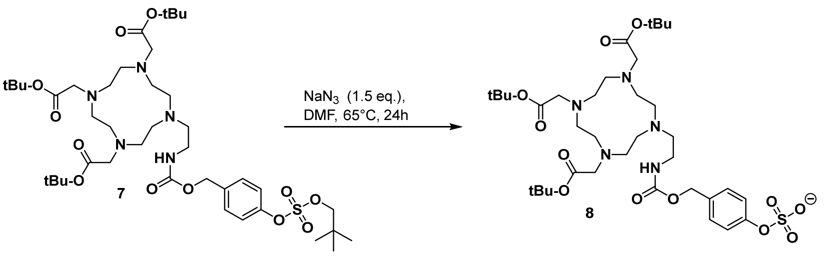

8: 4-((((2-(4,10-bis(2-(*tert*-butoxy)-2-oxoethyl)-7-(2-(neopentyloxy)-2-oxoethyl)-1,4,7,10-tetraazacyclododecan-1-yl)ethyl)carbamoyl)oxy)methyl)phenyl sulfate:

Compound **7** (1.20 g, 1.44 mmol, 1.00 eq.) was dissolved in DMF (7 mL), and sodium azide (140.0 mg, 2.16 mmol, 1.50 eq.) was added in portions. The reaction mixture was heated to 65 °C and stirred overnight. After completion, water was added and the reaction mixture was freeze-dried resulting in the crude compound **8,** as eggshell white powder. ^{1}H NMR (400 MHz, Methanol-d_4) δ 7.34 (d, J = 8.3 Hz, 2H), 7.27 (d, J = 7.9 Hz, 2H), 5.07 (s, 2H), 3.43 (d, J = 25.7 Hz, 12H), 2.98 (s, 4H), 2.75 (s, 9H), 1.48 (s, 27H). Analytical sample was purified giving 1.5 mg of material for ^{1}H analysis. Compound **8** was used in the next step without further purification.

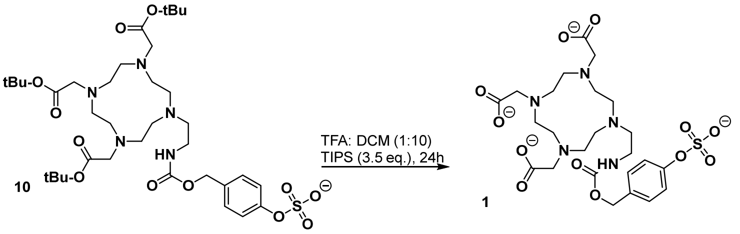

1: 2,2',2''-(10-(2-((((4-(sulfooxy)benzyl)oxy)carbonyl)amino)ethyl)-1,4,7,10-tetraazacyclodo-decane-1,4,7-triyl)triacetic acid:

Crude compound **8** (1.00 g, 1.25 mmol, 1.00 eq.) was dissolved in DCM (27 mL), and tri-*iso*-propylsilane (TIPS) (2.00 mL, 9.35 mmol, 3.50 eq.) was added. The reaction mixture was stirred and TFA (2.70 mL) was added. The resulting slightly white reaction mixture was left stirring at rt overnight. Upon complete deprotection (LCMS), the solvent was evaporated (with the rotary evaporator water bath set to temperature under 25 °C). The resulting sticky solid was washed with 3x 50 mL DCM, 3x 50 mL ether, 3x 50 mL ethyl acetate, 3x 50 mL pentane and 3x 50 mL ACN affording the crude compound **1** as an eggshell white powder. ^{1}H NMR (400 MHz, DMSO-d_6) δ 7.27 (d, J = 8.2 Hz, 2H), 7.16 (d, J = 8.3 Hz, 2H), 4.99 (s, 2H), 3.60 (s, 1H), 3.48 (s, 2H), 2.92 (d, J = 17.4 Hz, 1H), 2.73 (s, 1H), 2.50 (s, 199H), 2.24 (s, 1H), 2.08 (s, 1H) ppm. Compound **1** was used in the next step without further purification.

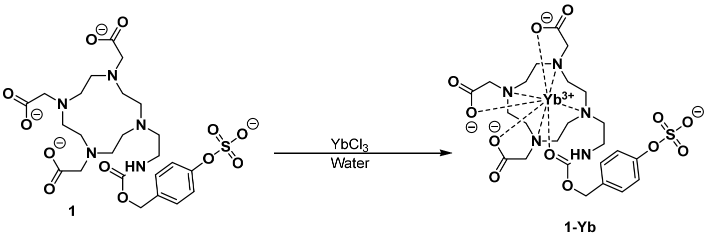

1-Yb:

A 100 mL flask was charged with compound **1** (600 mg, 0.970 mmol, 1.00 eq) and water was added (30 mL), giving rise to a suspension. Ytterbium chloride (301 mg, 0.78 mmol, 0.80 eq) was added to the suspension. Afterwards, the mixture was stirred and the pH was adjusted to 6.0 with 1 M LiOH. The reaction mixture was stirred for 1 d, the pH was adjusted to pH 6.5 and the level of complexation was checked with LCMS. A sample of the crude complex was purified by preparative HPLC, using program 3 (see general information section 1). The product (R_t = 11.5 min) was collected and freeze dried affording the desired compound **1-Yb** as a fluffy white solid. Isolated yield 3.4% (25 mg, 0.03 mmol).

Reaction was performed 3 times with yields ranging from 3.4-4.1%, compound **1-Yb** was stored at -20 °C in a closed vial. For ^{1}H-NMR see Figure S24. HRMS (ESI-) calc. for $[M-H]^{-}$ ($C_{24}H_{33}N_{5}O_{12}SYb^{-}$): 789.1240, found: 789.1242.

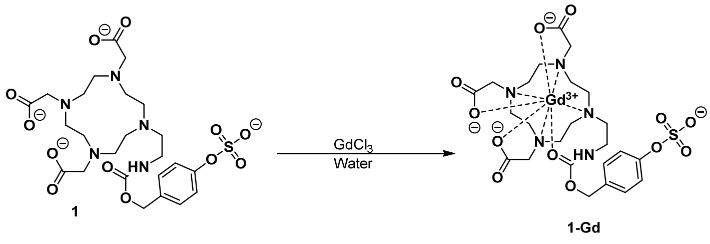

1-Gd:
A 100 mL flask was charged with compound **1** (300 mg, 0.49 mmol, 1.00 eq) and water was added (15 mL), giving rise to a suspension. Gadolinium chloride (102.0 mg, 0.38 mmol, 0.80 eq) was added. Afterwards, the mixture was stirred and the pH was adjusted to 6.0 with 1 M LiOH. The reaction mixture was stirred for 1 d, the pH was adjusted to pH 6.5 and the level of complexation was checked with LCMS. A sample of the crude complex was purified by preparative HPLC, using program 3 (see general information section 1). The product (R_t = 11.6 min) was collected and freeze dried affording the desired compound **1-Gd** as a fluffy white solid. Isolated yield 2.3% (8.4 mg, 0.01 mmol). Reaction was performed 2 times with yields ranging from 2.3-3.4 %, compound **1-Gd** was stored at -20 °C in a closed vial. For ^{1}H-NMR see Figure S25. HRMS (ESI-) calc. for $[M-H]^{-}$ ($C_{24}H_{33}N_{5}O_{12}SGd^{-}$): Exact Mass: 773.1093, found: 773.1102.

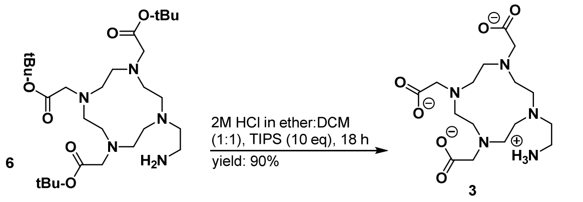

3: 2,2',2''-(10-(2-ammonioethyl)-1,4,7,10-tetraazacyclododecane-1,4,7-triyl)triacetate (IWE 149, 447):
Compound **8** (260 mg, 0.47 mmol, 1.00 eq) was dissolved in 10 mL DCM, and tri-*iso*-propylsilane (TIPS) (0.96 mL, 4.49 mmol, 10 eq.) was added. The reaction mixture was stirred and 2 M HCl in ether (10 mL) was added. The resulting slightly white reaction mixture was left stirring overnight at rt. Upon complete deprotection (LCMS), the solid was filtered off and washed with 3x 30 mL DCM, 3x 30 mL ether, 3x 30 mL ethyl acetate and 3x 30 mL pentane affording the compound **3** as slightly yellow powder. Isolated yield 90% (163 mg, 0.42 mmol). Reaction was performed 3 times with yields ranging from 89-92%, compound **3** was stored at -20 °C in a closed vial.^{1}H NMR (400 MHz, Deuterium Oxide) δ 4.10 – 4.05 (m, 3H), 3.57 (s, 7H), 3.48 (s, 1H), 3.39 (s, 1H), 3.15 (s, 6H), 3.04 (s, 3H) ppm. 1H NMR (400 MHz, Deuterium Oxide) δ 4.30 – 3.95 (m, 4H), 3.91 – 3.29 (m, 11H), 3.26 – 2.76 (m, 11H) ppm. ^{13}C NMR (151 MHz, Deuterium Oxide) δ 174.4, 168.5, 90.4, 85.8, 54.9, 53.0, 51.9, 50.5, 49.9, 48.5, 36.0 ppm. NMR spectra in agreement with the literature data.[4] HRMS (ESI+) calc. for $[M+H]^{+}$ ($C_{16}H_{32}N_{5}O_{6}^{+}$): 390.2347, found: 390.2345.

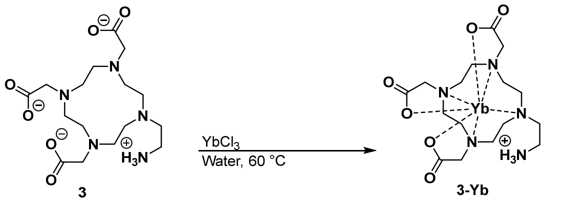

3-Yb:

A 20 mL vial was charged with compound **3** (100 mg, 0.256 mmol, 1.0 eq) dissolved in water (6 mL). Ytterbium chloride (79.4 mg, 0.205 mmol, 0.80 eq) was added, the vial was shaken, and the pH was adjusted to pH 6.0 with 1 M LiOH. Afterwards the reaction mixture was stirred at 60 °C for 24 h, the pH was adjusted to pH 6.5 and the complexation was checked with LCMS. The crude complex was freeze dried affording the desired compound **3-Yb** as a slightly yellow solid. Isolated yield 95 % (136.0 mg, 0.242 mmol). Compound **3-Yb** was stored at -20 °C in a closed vial. For ^{1}H-NMR see Figure S27. HRMS (ESI+) calc. for $[M+H]^+$ ($C_{16}H_{29}N_5O_6Yb^+$): 561.1500, found:561.1505

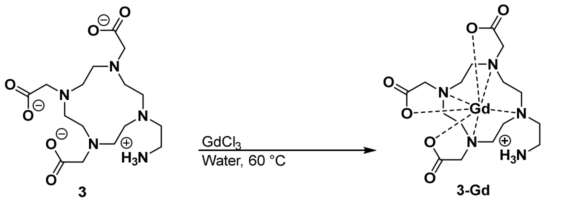

3-Gd:
A 20 mL vial was charged with compound **3** (100 mg, 0.256 mmol, 1.00 eq) dissolved in water (6 mL). Gadolinium chloride (54.0 mg, 0.205 mmol, 0.80 eq) was added, the vial was shaken and the pH was adjusted to pH 6.0 with 1 M LiOH. Afterwards the reaction mixture was stirred at 60 °C for 24 h, the pH was adjusted to pH 6.5 and the complexation was checked with LCMS. The crude complex was freeze dried affording the desired compound **3-Gd** as a yellow solid. Isolated yield 95 % (131.2 mg, 0,240 mmol). Compound **3-Gd** was stored at -20 °C in a closed vial. For ^{1}H-NMR see Figure S28., HRMS (ESI+) calc. for $[M+H]^+$ ($C_{16}H_{29}N_5O_6Gd^+$): 545.1353, found:545.1365.

3. Enzymatic hydrolysis:

Three enzyme hydrolysis where performed, enzyme reaction 1 (0.60 mg/mL), enzyme reaction 2 (0.13 mg/mL) and enzyme reaction 3 (0.63 mg/mL). The enzymatic hydrolysis reactions were analysed by following the reaction with UPLC-MS.

The following stock solutions were prepared for the reaction 1: Stock 1: 2.85 mg of compound **1-Yb** was dissolved in water (0.6 mL); Stock 2: NaCl (20 mg, 0.34 mmol) was dissolved in water (10 mL); Stock 3: sulfatase from *Helix pomatia* (3.4 mg, 55 units) was dissolved in stock 2 (2.73 mL). Stock 4: 10mM of triethylamine acetate (TEAA), with pH adjusted to 7.1 by adding acetic acid. Then the following reaction mixtures were prepared: Blank1: 0.1 mL of stock 1, 0.125 mL of stock 4, 0.2 mL of stock 2. Blank2: 0.1 mL of stock 1, 0.125 mL of stock 4, 0.2 mL of stock 2. Blank3: 0.1 mL of stock 1, 0.125 mL of stock 4, 0.2 mL of stock 2. Enzyme reaction A1: 0.1 mL of stock 1, 0.125 mL of stock 4 and 0.2 mL of stock 3. Enzyme reaction A2: 0.1 mL of stock 1, 0.125 mL of stock 4 and 0.2 mL of stock 3. Enzyme reaction A3: 0.1 mL of stock 1, 0.125 mL of stock 4 and 0.2 mL of stock 3. The reactions were heated at 37.5 °C and were analyzed by UPLC-MS after certain intervals. On Acquity UPLC-MS with TQD fitted with a BEH C8 column (1.7 uM 2.1 x 50 mm). Using Program 3: Eluent A: acetonitrile Eluent B: water, both with 0.1% v/v formic acid added. Program 0-2 min 2% of eluent A, from 2-10 gradient 2-90% eluent A, 10-15 min flush of 100% A, 15-20 min going back to 2% A. Raw data set are shown in supporting information table 1 and 2, visualized in Figure 3c of the manuscript. The pH after the enzymatic reaction was 7.1.

The following stock solutions were prepared for enzyme reaction 2: Stock 1: 2.70 mg of compound **1-Yb** was dissolved in water (0.55 mL); Stock 2: NaCl (20 mg, 0.34 mmol) was dissolved in water (10 mL); Stock 3: sulfatase from *Helix pomatia* (1.2 mg, 19.36 units) was dissolved in stock 2 (4.4 mL). Stock 4: 10mM of triethylamine acetate (TEAA), with pH adjusted to 7.1 by adding acetic acid. Then the following reaction mixtures were prepared: Blank : 0.1 mL of stock 1, 0.125 mL of stock 4, 0.2 mL of stock 2. Blank2 : 0.1 mL of stock 1, 0.125 mL of stock 4, 0.2 mL of stock 2. Enzyme reaction 2-1: 0.1 mL of stock 1, 0.125 mL of stock 4 and 0.2 mL of stock 3. Enzyme reaction 2-2: 0.1 mL of stock 1, 0.125 mL of stock 4 and 0.2 mL of stock 3.

The following stock solutions were prepared for enzyme reaction 3: Stock 1: 2.70 mg of compound **1-Yb** was dissolved in water (0.55 mL); Stock 2: NaCl (20 mg, 0.34 mmol) was dissolved in water (10 mL); Stock 3: sulfatase from *Helix pomatia* (7.45 mg, 120 units) was dissolved in stock 2 (4.4 mL). Stock 4: 10mM of triethylamine acetate (TEAA), with pH adjusted to 7.1 by adding acetic acid.

Then the following reaction mixtures were prepared: Blank1 : 0.1 mL of stock 1, 0.125 mL of stock 4, 0.2 mL of stock 2. Blank2 : 0.1 mL of stock 1, 0.125 mL of stock 4, 0.2 mL of stock 2. Enzyme reaction 3-1: 0.1 mL of stock 1, 0.125 mL of stock 4 and 0.2 mL of stock 3. Enzyme reaction 3-2: 0.1 mL of stock 1, 0.125 mL of stock 4 and 0.2 mL of stock 3.

Both enzyme reactions (2 and 3) were heated at 37.5 °C and were analyzed by UPLC-MS after certain intervals. This performed on a Thermo Scientific vanquish UHPLC with ACQUITY UPLC BEH C8, 2.1mm x 150 mm 1.7μm particles column and Thermo Scientific LCQ fleet ion trap as mass detector. Solvent: Water: ACN with in both solvents 0.1% formic acid; Method: 17 min, 0-2 min 5% ACN, from 2-10 gradient to 90% ACN, followed by a flush of 100% 10-12 min, 12-17 min going back to 5% ACN. Results are shown in table 3-5, visualized in Figure S2-4 of the manuscript. The pH after the enzymatic reaction was 7.1

Table S1. LCMS analysis of sulfatase enzyme reaction 1, of **1-Yb** in TEAA buffer pH 7.1. Peak Areas of **1-Yb** and **3-Yb**. for every run. In the time intervals 0, 15, 30, 60, 120, 180, 240 min. (Triplicate)

min	*Area 1-Yb*	*Area 1-Yb*	*Area 1-Yb*	*SD*	*1-Yb average*
0	55655976	54520148	54107972	801697.2	54761365
15	49112036	48626848	48912224	243848.2	48883703
30	44402656	43207228	44088880	619786.8	43899588
60	34787168	35738160	35536592	501107.4	35353973
120	13479848	13121169	13222116	184962.5	13274378
180	9342721	9254473	9448355	97070.83	9348516
240	5791907	5984247	5382287	307446.2	5719480

min	*Area 3-Yb*	*Area 3-Yb*	*Area 3-Yb*	*SD*	*3-Yb average*
0	13385	14113	15205	916.0466	14234.33
15	39017	65006	67617	15812.47	57213.33
30	94816	116410	147820	26653.06	119682
60	257532	240736	208432	24954.84	235566.7
120	463213	467391	514524	28495.01	481709.3
180	614535	551365	597079	32621.39	587659.7
240	626563	631462	516975	591666.7	591666.7

Table S2. LCMS analysis of the blanks of enzyme reaction 1, of **1-Yb** in TEAA buffer pH 7.1. Peak Areas of **1-Yb** for every run. In the time intervals 0, 15, 30, 60, 120, 180, 240 min. (Triplicate)

min	*Area 1-Yb*	*Area 1-Yb*	*Area 1-Yb*	*SD*	*1-Yb average*	*Area 3-Yb*	*Area 3-Yb*	*Area 3-Yb*
0	52642931	52135576	52050532	320306.7	52276346.33	ND	ND	ND
15	52598772	52818426	52898028	154993.8	52771742	ND	ND	ND
30	52440248	53866768	52885884	729804.1	53064300	ND	ND	ND
60	52712248	53512684	52880056	422112.8	53034996	ND	ND	ND
120	53733404	53202768	53778024	320022.1	53571398.67	ND	ND	ND
180	52820386	52066452	52533052	380502.5	52473296.67	ND	ND	ND
240	52942040	52926960	52677036	148838	52848678.67	ND	ND	ND

ND: not detected in TIC file.

Table S3. LCMS analysis of sulfatase enzyme reaction 2, of **1-Yb** in TEAA buffer pH 7.1. Peak Areas of **1-Yb** and **3-Yb**. for every run. In the time intervals 1, 5, 15, 30, 60, 120, 180, 240, 420, 1080 min. (Duplicate)

min	*Area 1-Yb*	*Area 3-Yb*	*Area 1-Yb*	*Area 3-Yb*
1	12863.36	212.69	13139.48	382.94
5	12857.76	579.43	12689.16	649.38
15	11602.95	1002.25	12006.90	1231.42
30	10788.92	1553.66	11732.97	1465.95
60	9884.77	2467.12	9930.29	2636.46
120	7155.47	3816.83	7524.51	3306.18
180	6518.46	4703.64	6473.79	4895.29
240	5648.57	5186.97	5449.38	4888.85
420	3872.98	5787.61	4229.24	5866.88
1080	664.69	7320.89	620.01	7763.30

Table S4. LCMS analysis of sulfatase enzyme reaction 3, of **1-Yb** in TEAA buffer pH 7.1. Peak Areas of **1-Yb** and **3-Yb**. for every run. In the time intervals 0, 3, 5, 15, 30, 60, 120, 180, 240 and 420 min. (Duplicate)

time	*Area of 1-Yb*	*Area of 3-Yb*	*Area of 1-Yb*	*Area of 3-Yb*
0	10684.30	427.02	9979.83	315.78
3	8476.84	1204.67	7554.43	1320.24
5	6945.07	1208.33	6582.00	1345.87
15	4268.54	2184.27	5030.38	2563.10
30	2618.63	3916.05	2805.23	3737.56
60	1297.04	5007.98	1349.66	4438.49
120	161.92	5202.96	180.76	5001.99
180	63.59	5025.42	123.56	5359.58
240	0.00	5954.35	0.00	5195.64
420	0.00	5556.40	0.00	4530.53

Table S5. LCMS analysis of the blank of enzyme reaction 2, of **1-Yb** in TEAA buffer pH 7.1. Peak Areas of **1-Yb** and **3-Yb**. for every run. In the time intervals 15, 30, 60, 120, 180, 420, 1080 min.

time	*Area 1-Yb*	*Area 3-Yb*
15	14148.50	186.37
30	13757.97	242.18
60	13580.00	237.89
120	14150.96	179.79
180	13488.41	144.67
420	13758.68	250.99
1080	15674.92	103.43

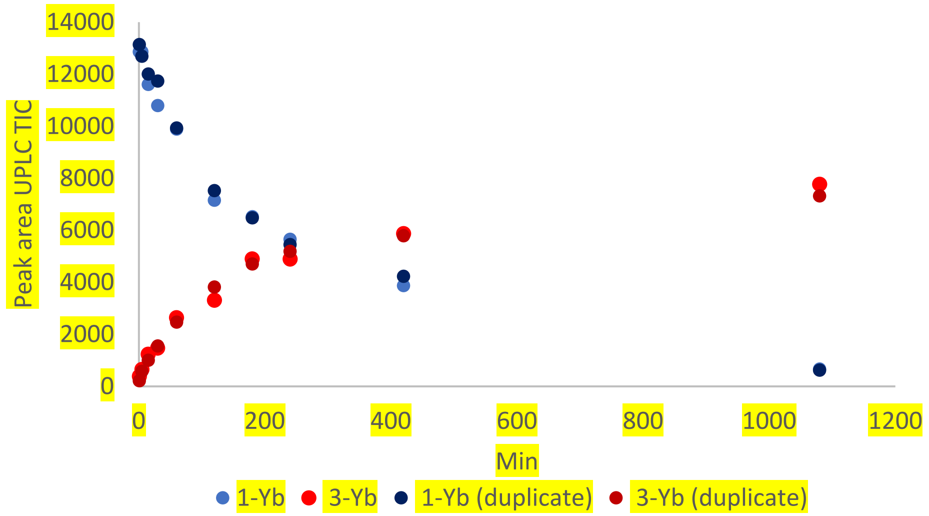

Figure S2. The sulfatase-catalyzed hydrolysis of **1-Yb** (1.46 mM in 3 mM TEAA buffer at 37 °C, pH 7.1) followed by UPLC-MS with 0.13 mg/mL of added enzyme (Enzyme reaction 2). Hydrolysis reaction was performed in duplicate (reaction 2-1: substrate shown in light blue product formation in light red; reaction 2-2: substrate shown in dark blue, product formation in dark red)

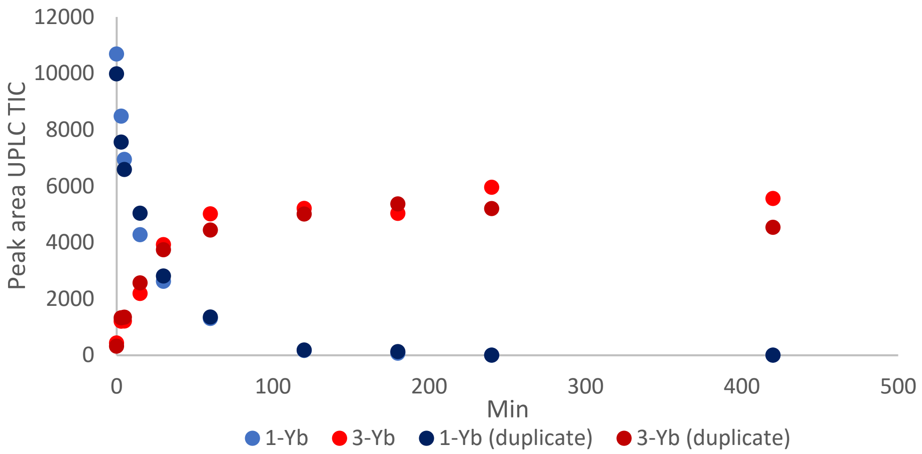

Figure S3. The sulfatase-catalyzed hydrolysis of **1-Yb** (1.46 mM in 3 mM TEAA buffer at 37 °C, pH 7.1) followed by UPLC-MS with 0.63 mg/mL of added enzyme (Enzyme reaction 3). Hydrolysis reaction was performed in duplicate (reaction 3-1: substrate shown in light blue product formation in light red; reaction 3-2: substrate shown in dark blue, product formation in dark red)

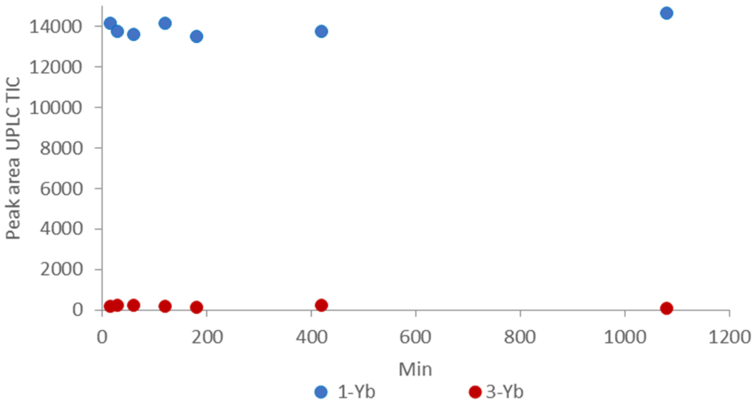

Figure S4. Stability test of **1-Yb** in 3 mM of TEAA buffer in the absence of the enzyme at 37 °C, pH 7.1.

4. NMRD profiles

NMRDs were recorded on a Stelar 0.25T FFC SMARtracer relaxometer (Mede, Italy). The relaxation rates were determined over a (proton) Larmor frequency range of 0.01–10 MHz at 37 °C with 17 data points collected. A stock solution of **1-Gd** was prepared (1 mM in 3 mL of 3 mM TEAA buffer, pH 7.35) and divided into two samples: blank and the enzymatic (sulfatase) reaction. NMRD profiles were recorded before adding the enzyme (0.086 mg/mL, 1.38 unit of sulfatase from *Helix pomatia*) and after 28 h and 56 h. Furthermore, a solution **3-Gd** (1 mM in 3 mL of 3 mM TEAA buffer, pH 7.35) was also measured. In addition, the stability of the blank was assessed by repeating the analysis after leaving the sample for 28 h and 56 h at 37 °C without enzyme. The NMRD profile with more enzyme was collected by adding sulfatase from *Helix pomatia* (0.46 mg/mL, 7.42 units) to the blank, and a NMRD profile was recorded at 30, 120, 180, 240, 300 and 1440 min.(table 6-7) From both enzyme and blank reactions, samples were taken to analyse compounds present via UPLC-MS. (Figure S8-9)

The stability of compound **1-Gd** in the presence of esterase from porcine liver was also assessed. A stock solution of **1-Gd** was prepared (1 mM in 3 mL of 3 mM TEAA buffer, pH 7.34) and divided into two samples: blank and the enzymatic reaction. NMRD profiles were recorded before adding the enzyme (1.18 mg/mL, 73.8 units of esterase), and a NMRD profile was recorded at 30, 120, 180, 240 and 1440 min (table 8, Figure S6). From both enzyme and blank reactions, samples were taken to analyse compounds present via UPLC-MS. (Figure S11-12). In Figure S4 a comparison is shown between the sulfatase relaxivity results and the esterase relaxivity results.

The relaxation rates of **1-Gd**, **3-Gd** and sulfatase hydrolysis at 4.7T were recorded with inversion recovery method at 37 °C. A stock solution of **1-Gd** was prepared (1 mM in 1 mL of 3 mM TEAA buffer consistent of 10% D_2O, pH 7.35) and divided into two samples: blank and the enzymatic reaction to which sulfatase from *Helix pomatia* (0.46 mg/mL, 7.42 units) was added. The solutions where then transferred to a 3 mm sample tubes (0.25 mL per tube) and measured. Every data point measured in duplicate (table 9).

Table S6. Molar relaxivity (s^{-1} mM^{-1}) profiles of a sample of **1-Gd** in TEAA buffer pH 7.35. Blank t=0, Blank 28 h, Blank 56h. Enzyme reaction t=0, Enzyme reaction t=28 h and Enzyme reaction t=56 h. (sulfatase 0.086 mg/mL)

MHz	*blank t=0*	*blank 28 h*	*t=28 h*	*blank 56 h*	*t= 56 h*	*3-Gd*
10.00	3.7907	3.7531	3.5301	3.7497	3.0645	2.5821
6.31	4.2298	4.3115	3.6157	4.1847	3.5517	2.9863
3.98	4.2872	4.2409	3.7902	4.356	3.6828	3.4277
2.51	4.5358	4.4809	4.0455	4.8712	4.2274	3.5783
2.51	4.681	4.3767	4.4137	4.5967	4.0887	3.6048
1.58	4.6673	4.8044	4.2454	4.9469	4.0885	3.6006
0.99	4.8652	5.0276	4.596	5.012	4.2182	3.6769
0.63	5.0652	4.9871	4.3487	5.0304	4.3342	3.7565
0.39	4.9131	5.0004	4.4955	4.8978	4.1563	3.6311
0.25	5.0381	4.939	4.3514	5.0592	4.2791	3.8449
0.16	5.0871	5.0045	4.6539	5.0597	4.2848	3.7449
0.10	4.8536	4.93	4.4047	5.1813	4.2453	3.8059
0.06	4.9471	5.181	4.4689	5.2158	4.3637	3.7666
0.04	4.998	5.0021	4.7297	4.9059	4.254	3.7308
0.03	4.8648	5.2236	4.2971	5.2194	4.301	3.7149
0.02	5.0057	5.033	4.396	5.2286	4.185	3.6296
0.01	5.2824	4.9672	4.768	5.0902	4.2927	3.6936

Table S7. Molar relaxivity (s^{-1} mM^{-1}) profiles of a sample of **1-Gd** in TEAA buffer pH 7.35, time points, t=0, 30, 120, 180,240,300 and 1440 min. (sulfatase 0.46 mg/mL)

MHz	*t=0*	*t=30 min*	*t=120 min*	*t=180 min*	*t=240 min*	*t=300 min*	*t=1440 min*
10.00	4.0059	3.8321	3.5049	3.5672	3.1114	3.0537	3.1278
6.31	4.3922	4.2882	4.0154	3.7158	3.7081	3.3438	3.3397
3.98	4.5909	4.6885	4.0922	4.4352	4.1575	3.8466	3.7051
2.51	4.539	5.0231	4.4837	4.4619	4.1539	4.2592	4.1716
2.51	4.8709	4.6213	4.3601	4.5645	3.9694	3.9299	4.1773
1.58	4.9146	4.8925	4.6428	4.7988	4.1906	4.2675	4.4845
0.99	4.7945	5.1548	4.7353	4.7109	4.5531	4.4854	4.3434
0.63	5.1674	4.9713	4.7521	4.6745	4.4241	4.5717	4.4791
0.39	4.9535	4.8589	5.0359	4.7186	4.5726	4.5498	4.8507
0.25	4.9742	5.3343	4.837	4.8954	4.6494	4.5906	4.7742
0.16	5.0824	4.6614	4.7984	4.9033	4.5076	4.6036	4.7055
0.10	5.2304	4.949	4.9597	4.7237	4.3205	4.3872	4.7474
0.06	4.969	4.9418	4.7862	4.6651	4.6729	4.4245	4.7522
0.04	5.2384	5.0952	4.7144	4.4947	4.4909	4.4113	4.8711
0.03	5.1736	5.0745	4.5676	4.6457	4.3892	4.4847	4.3705
0.02	5.3059	4.9173	5.1393	4.5506	4.2538	4.3716	4.4919
0.01	5.0175	4.9473	4.4904	4.4186	4.5361	4.4155	4.7152

Table S8. Molar relaxivity (s^{-1} mM^{-1}) profiles of a sample of **1-Gd** in TEAA buffer pH 7.34, time points, t=0, 30, 120, 180,240,300 and 1440 min. (esterase 1.18 mg/mL)

MHz	*t=0*	*t=0 after 24 h*	*t= 30 min*	*t= 60 min*	*t= 120 min*	*t=180 min*	*t=240 min*	*t=1440 min*
10.00	3.9240	3.9373	4.1380	4.2136	4.2694	4.1273	4.2206	4.2254
6.31	4.2133	4.3027	4.4061	4.5946	4.4338	4.3878	4.2278	4.5903
3.981	4.5165	4.6161	4.6006	4.3817	4.2951	4.3510	4.4614	4.3723
2.51	4.8992	4.7008	4.9219	4.7293	4.6202	4.6171	4.6698	4.7133
2.51	4.5862	4.8061	5.0671	5.1517	4.8844	4.5062	4.5639	5.2327
1.58	4.9652	4.9827	5.0330	4.8700	4.8760	4.7564	4.8712	5.0892
0.99	4.8954	4.9365	5.0720	4.9578	5.0358	4.6654	5.2024	4.9743
0.63	5.0474	4.8844	5.0603	5.0112	5.0081	5.3054	5.0757	5.3064
0.39	5.1320	5.1240	5.1419	5.3587	5.2120	5.0272	5.3819	5.1112
0.25	5.0120	5.2130	5.1549	5.5829	5.1364	5.2044	5.2475	5.3316
0.16	5.0428	5.0859	5.4191	5.1323	5.2484	5.1030	5.2763	5.2227
0.10	5.0683	4.9142	5.3819	5.1766	5.2119	5.4354	5.1241	5.2186
0.06	5.0664	4.9781	5.5505	5.4054	5.3174	5.3486	5.2359	5.2785
0.04	4.9007	5.0752	5.4120	4.9889	5.3311	5.1867	5.3692	5.1663
0.03	5.0048	5.1107	5.2115	5.3018	5.0029	5.3156	4.8727	5.3777
0.02	5.1244	5.0765	5.0342	5.6386	5.1956	5.2996	5.2830	5.3643
0.01	5.0894	5.1315	5.1801	5.2824	5.1232	4.8547	5.2702	5.3457

Table S9. Molar relaxivity (s^{-1} mM^{-1}) profiles of a sample of **1-Gd** in TEAA buffer pH 7.35 at 4.7 T. Blank t=0, Blank 24 h, Enzyme (sulfatase) reaction t= 30, 50 min 60 min 120 min, 180 min, 24 h. All points measured in duplicate.

Blank t=0	*t= 30 min*	*t=50 min*	*t=60 min*	*t=120 min*	*t=180 min*	*t=24 h*	*3-Gd*	*blank t= 24 h*
3.4560	2.0252	2.0252	2.0062	2.0143	2.0153	1.9884	1.6689	3.4654
3.4561	2.0291	2.0111	2.0037	2.0073	2.0081	1.9925	1.6643	3.4532

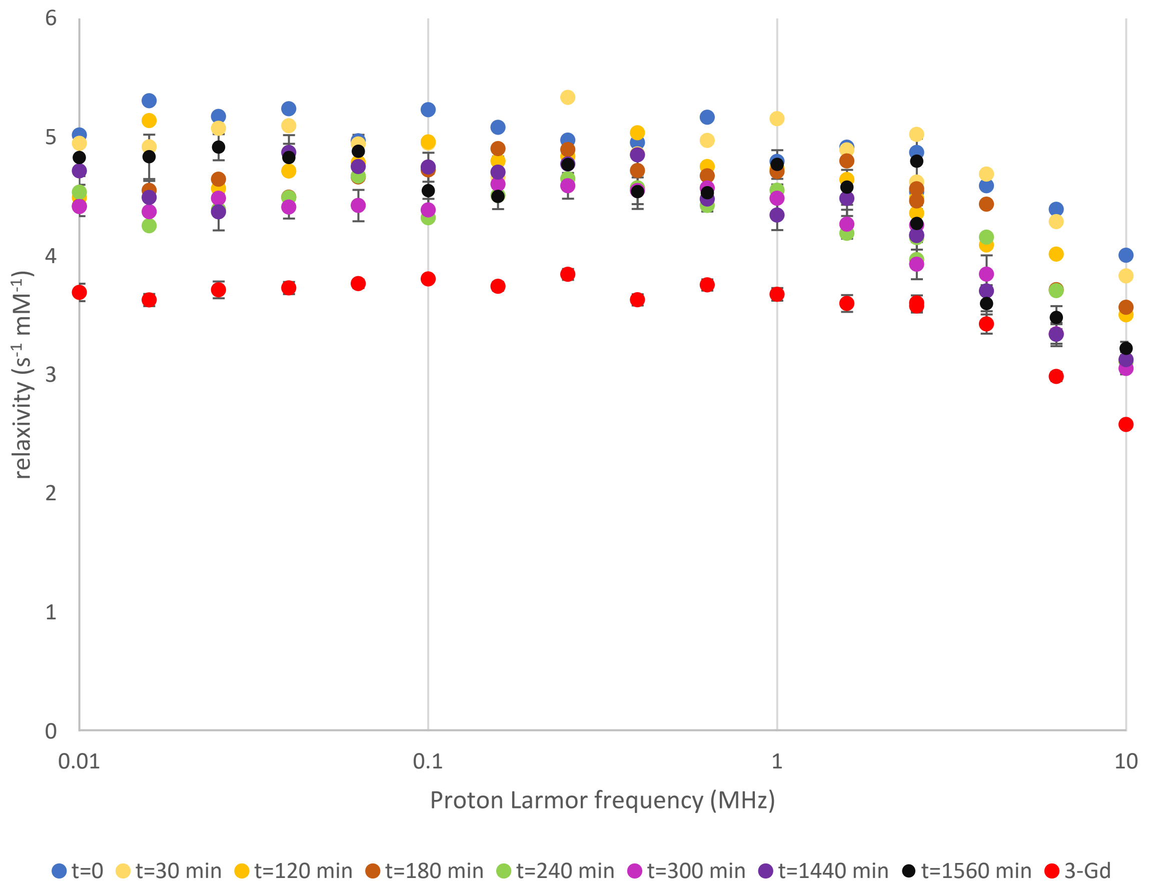

Figure S5. NMRD relaxometric analysis of **1-Gd** (1.0 mM) in TEAA buffer (3 mM) at 37 °C with the addition of 0.086 mg/mL of enzyme followed over 56 h, including **3-Gd**. Error bars represent uncertainty of fitting the T_1 curve to the experimental data.

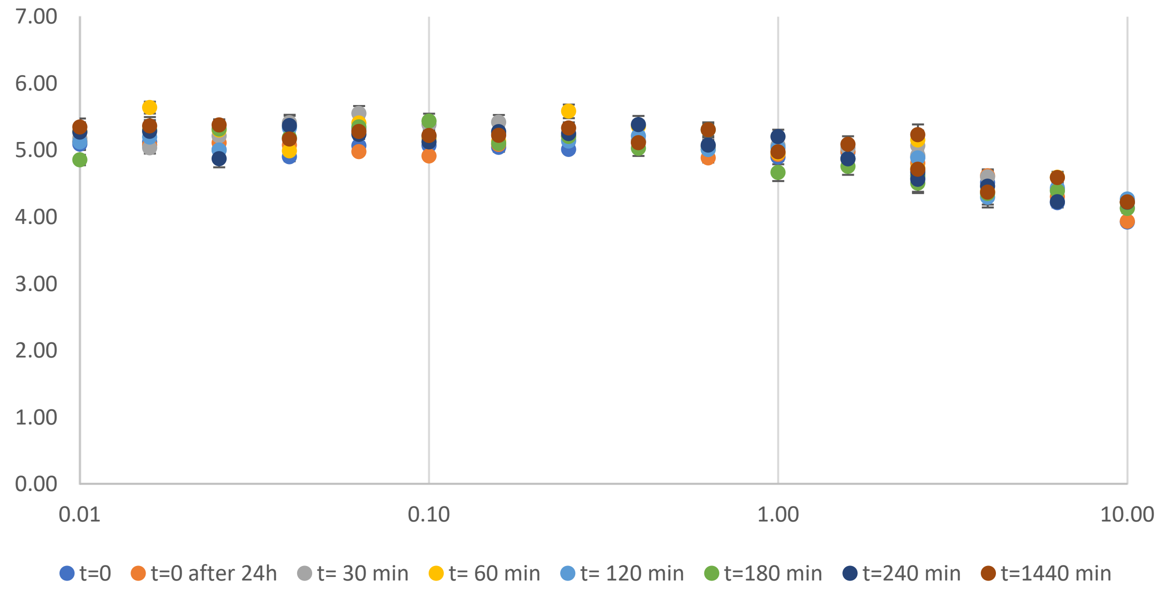

Figure S6. NMRD relaxometric analysis of **1-Gd** (1.0 mM) in TEAA buffer (3 mM) at 37 °C with the addition of 1.18 mg/mL of esterase from porcine liver followed over 24 h. Error bars represent uncertainty of fitting the T_1 curve to the experimental data.

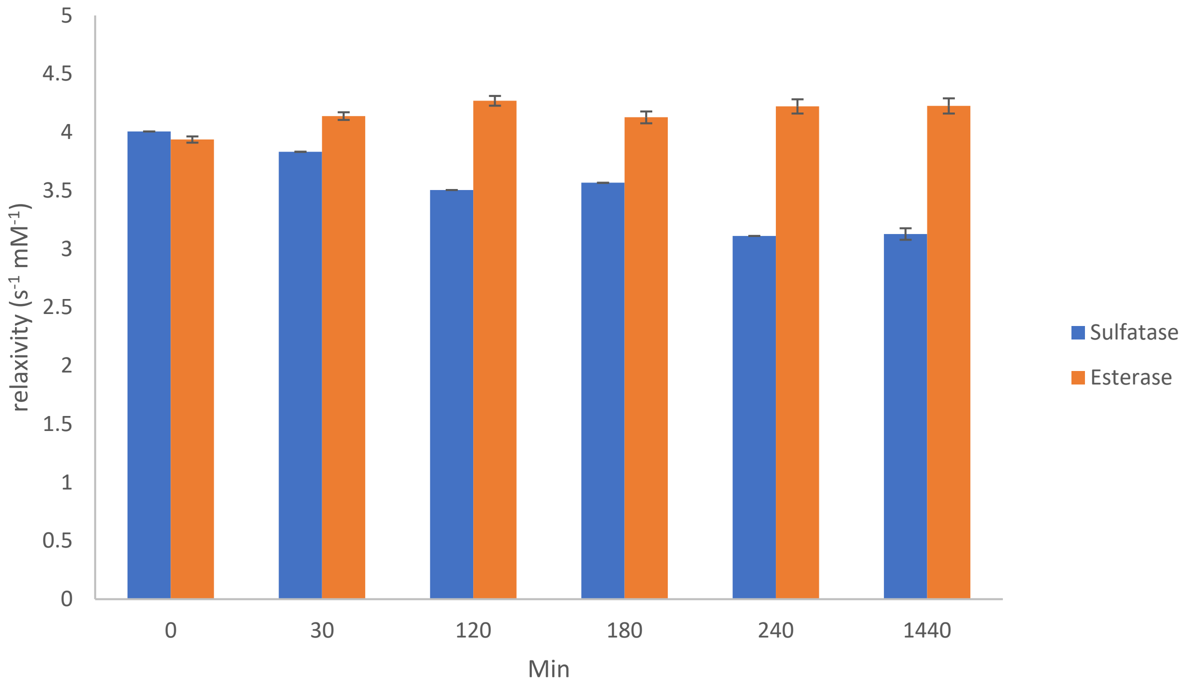

Figure S7. NMRD relaxometric analysis of **1-Gd** (1.0 mM) in TEAA buffer (3 mM) at 37 °C with the addition of 1.18 mg/mL of esterase from porcine liver followed over 24 h. vs the NMRD relaxometric analysis of **1-Gd** (1.0 mM) in TEAA buffer (3 mM) at 37 °C with the addition of 0.46 mg/mL of sulfatase followed over 24 h. Error bars represent uncertainty of fitting the T_1 curve to the experimental data.

UPLC analysis of the selected samples from the NMRD profile measurements.

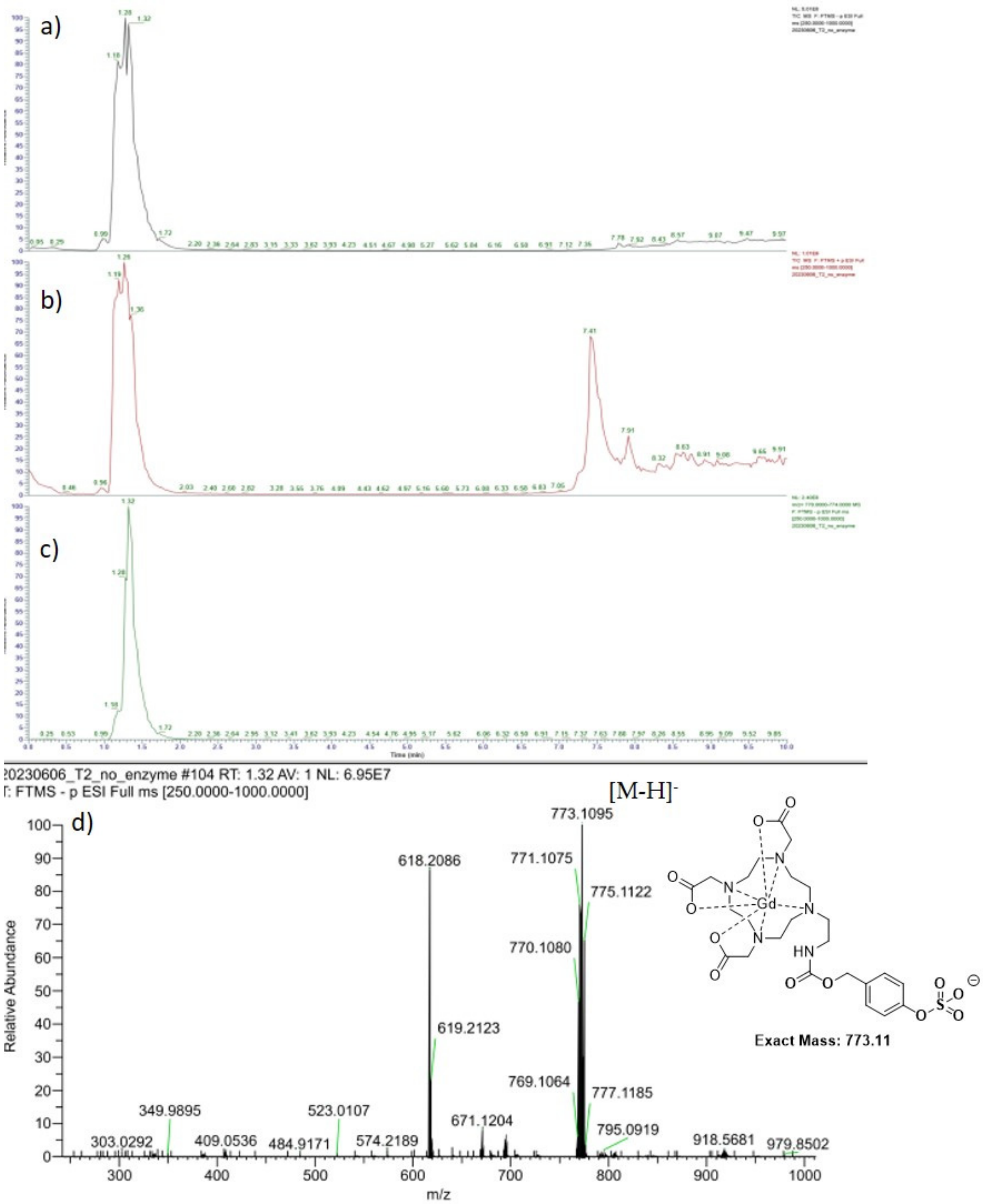

Figure S8. UPLC-MS analysis of blank **1-Gd** before adding sulfatase. (a) total ion current chromatogram negative mode, (b) total ion current chromatogram positive mode), (c) 770-774 mass trace, (d) mass spectrum of the peak at R_t = 1.32 min.

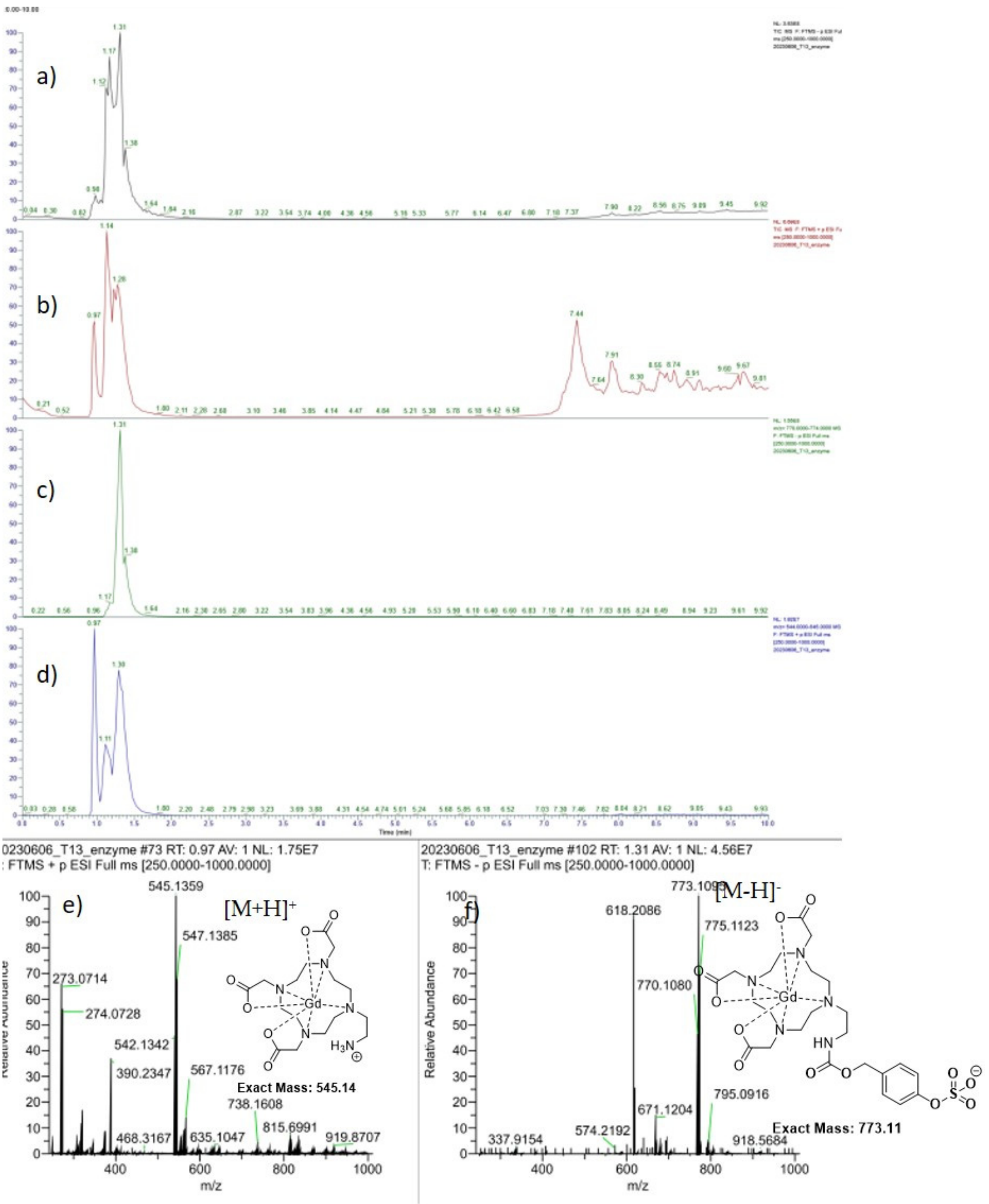

Figure S9. UPLC-MS analysis of sulfatase hydrolysis reaction (0.086 mg/mL) with **1-Gd** t=28h. (a) total ion current chromatogram negative mode, (b) total ion current chromatogram positive mode), (c) 770-774 mass trace, (d) 540-544 mass trace, (e) mass spectrum of the peak at R_t = 1.31 min. (f) mass spectrum of the peak at R_t = 0.97 min.

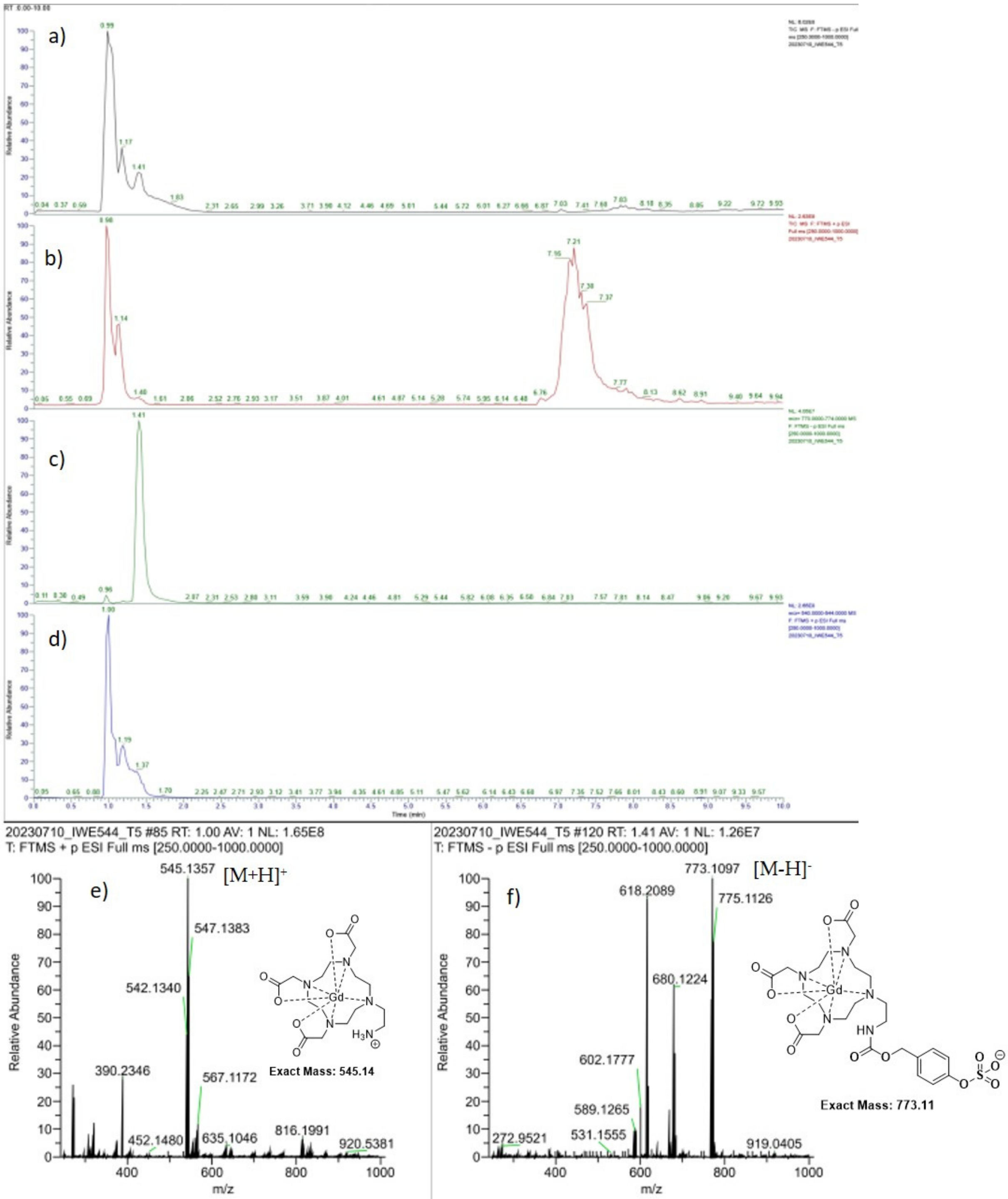

Figure S10. UPLC-MS analysis of sulfatase hydrolysis (0.49 mg/mL) of **1-Gd** t=300 min. (a) total ion current chromatogram negative mode, (b) total ion current chromatogram positive mode), (c) 770-774 mass trace, (d) 540-544 mass trace, (e) mass spectrum of the peak at R_t = 0.98 min. (f) mass spectrum of the peak at R_t = 1.41 min.

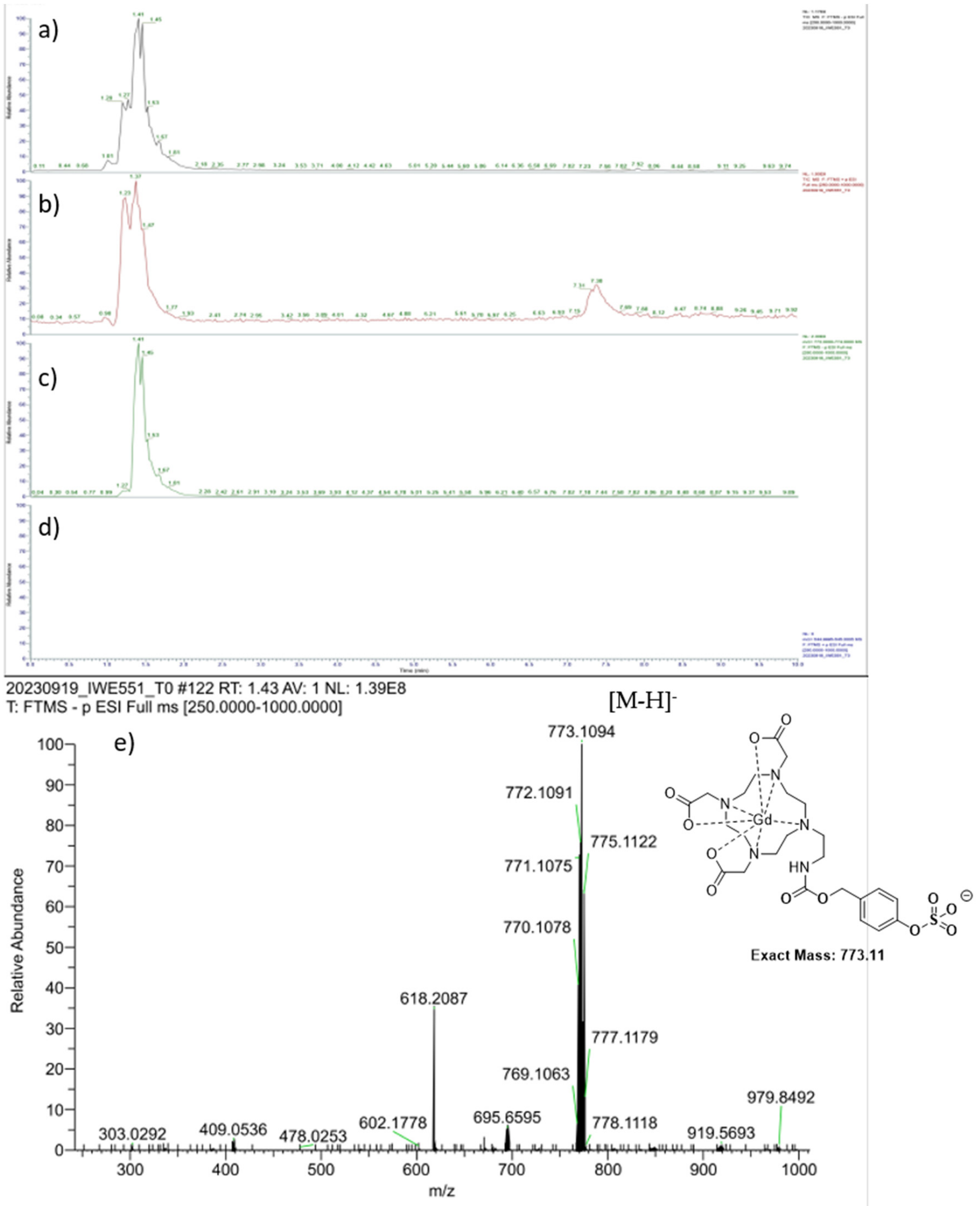

Figure S11. UPLC-MS analysis of blank **1-Gd** before adding esterase. (a) total ion current chromatogram negative mode, (b) total ion current chromatogram positive mode), (c) 770-774 mass trace, (d) 544-545 mass trace (e) mass spectrum of the peak at R_t = 1.43 min.

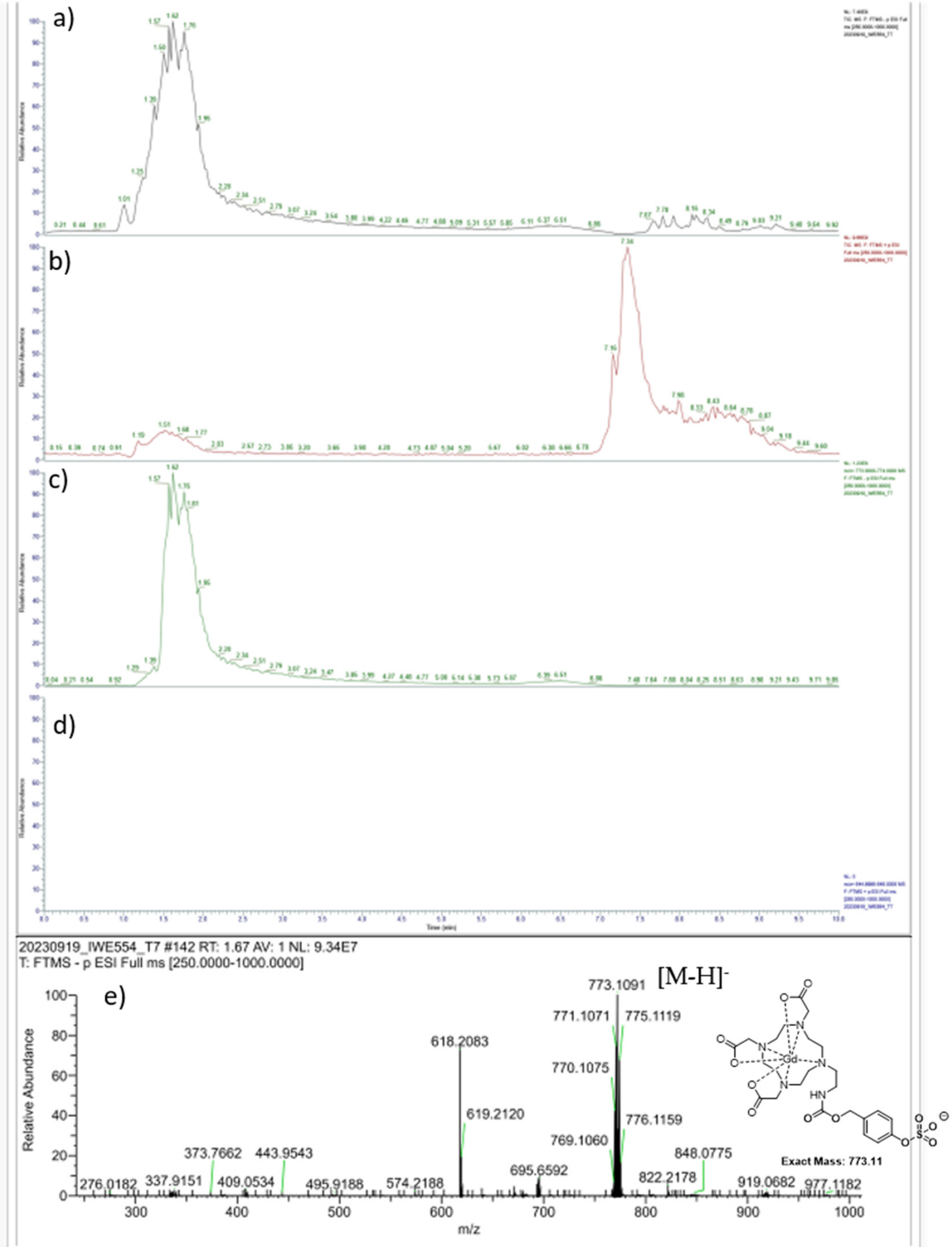

Figure S12. UPLC-MS analysis of **1-Gd**, 24 h (1440 min) after adding esterase. (a) total ion current chromatogram negative mode, (b) total ion current chromatogram positive mode), (c) 770-774 mass trace, (d) 544-545 mass trace, (e) mass spectrum of the peak at R_t = 1.67 min.

5. Z-spectra

All Z-spectra were recorded on a Varian Oxford AS 500 MHz (B_0 = 11.7T), using 5 mm sample tubes. A stock solution of **1-Yb** was prepared (20 mM in 1.5 mL of 3 mM TEAA buffer with 10% D_2O) this stock solution was then divided into two samples, blank and the enzymatic reaction (0.75 mL each). The Z-spectra were recorded before adding the enzyme (0.33 mg/mL 5.32 units of sulfatase) and after 24 h and 48 h. In addition, the stability of the blank was assessed by repeating the analysis after leaving the sample for 24 h and 48 h at 37 °C without enzyme.

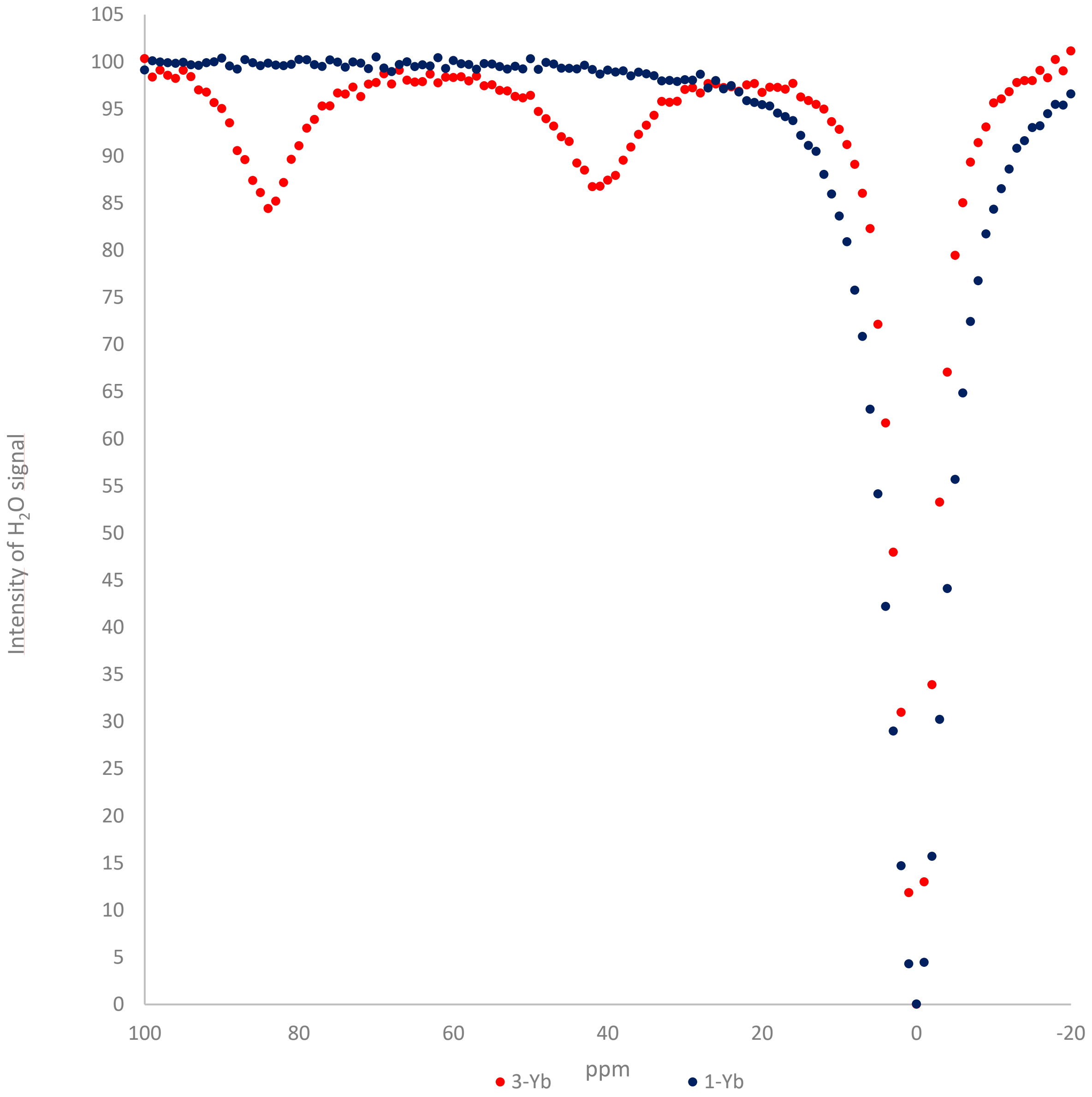

Figure S13. Z-spectra of **1-Yb** overlapped on **3-Yb** (20 mM in water with 10% D_2O, pH adjusted to 7.4 with 1 mM LiOH, B_0 = 11.7 T, satpwr = 28 dB, satdly = 2 s) at T = 37 °C.

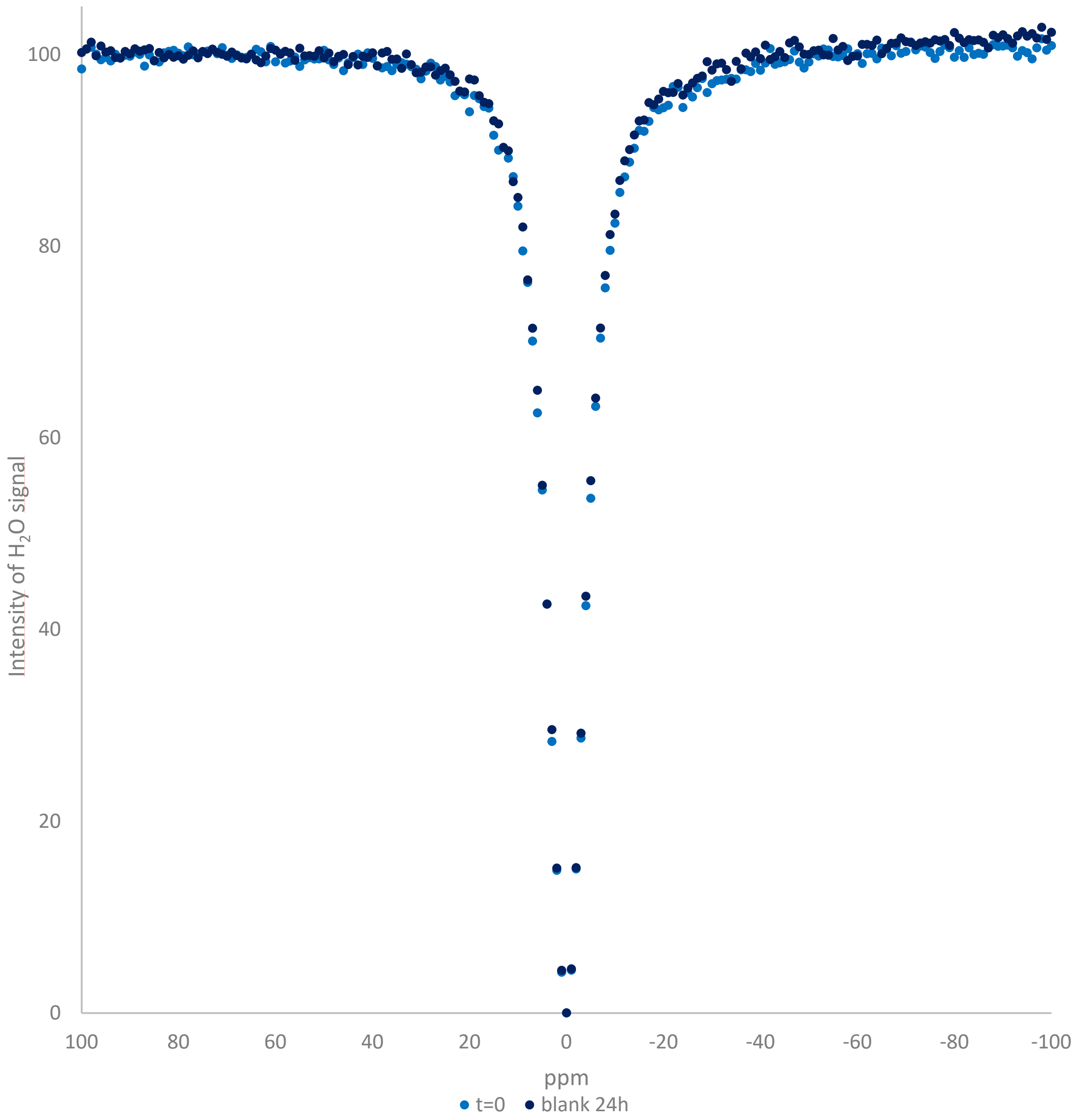

Figure S14. Z-spectra of the solution of **1-Yb** at t = 0 and at t = 24 h incubation at 37 °C (20 mM in 3 mM TEAA buffer with 10% D_2O, pH 7.40, B_0 = 11.7 T, satpwr = 28 dB, satdly = 2 s).

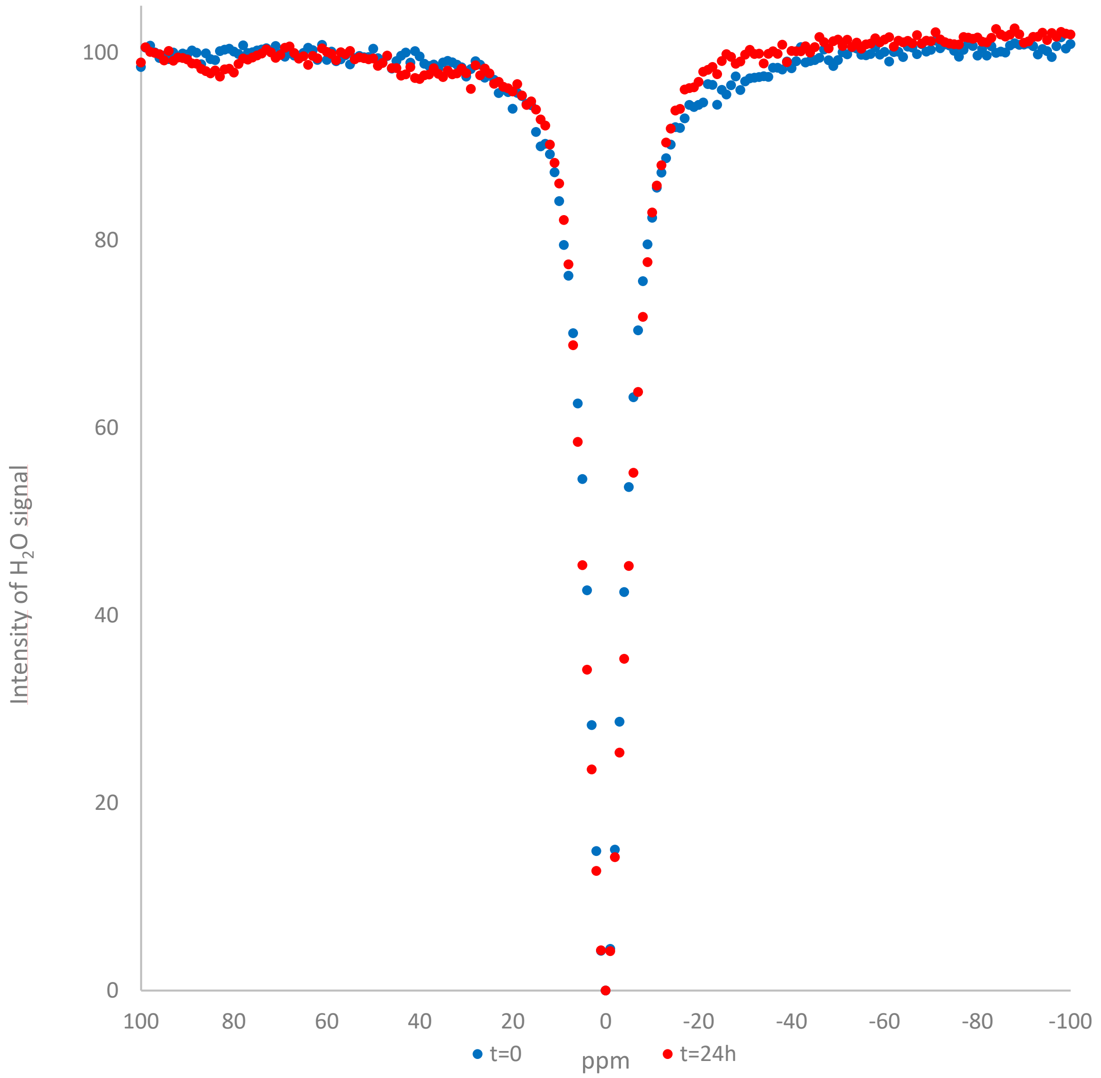

Figure S15. Z-spectra of the enzymatic reaction of **1-Yb** at t=0 and at t=24 h incubation at 37 °C (20 mM in 3 mM TEAA buffer with 10% D_2O, pH 7.4, 0.33 mg/mL sulfatase.

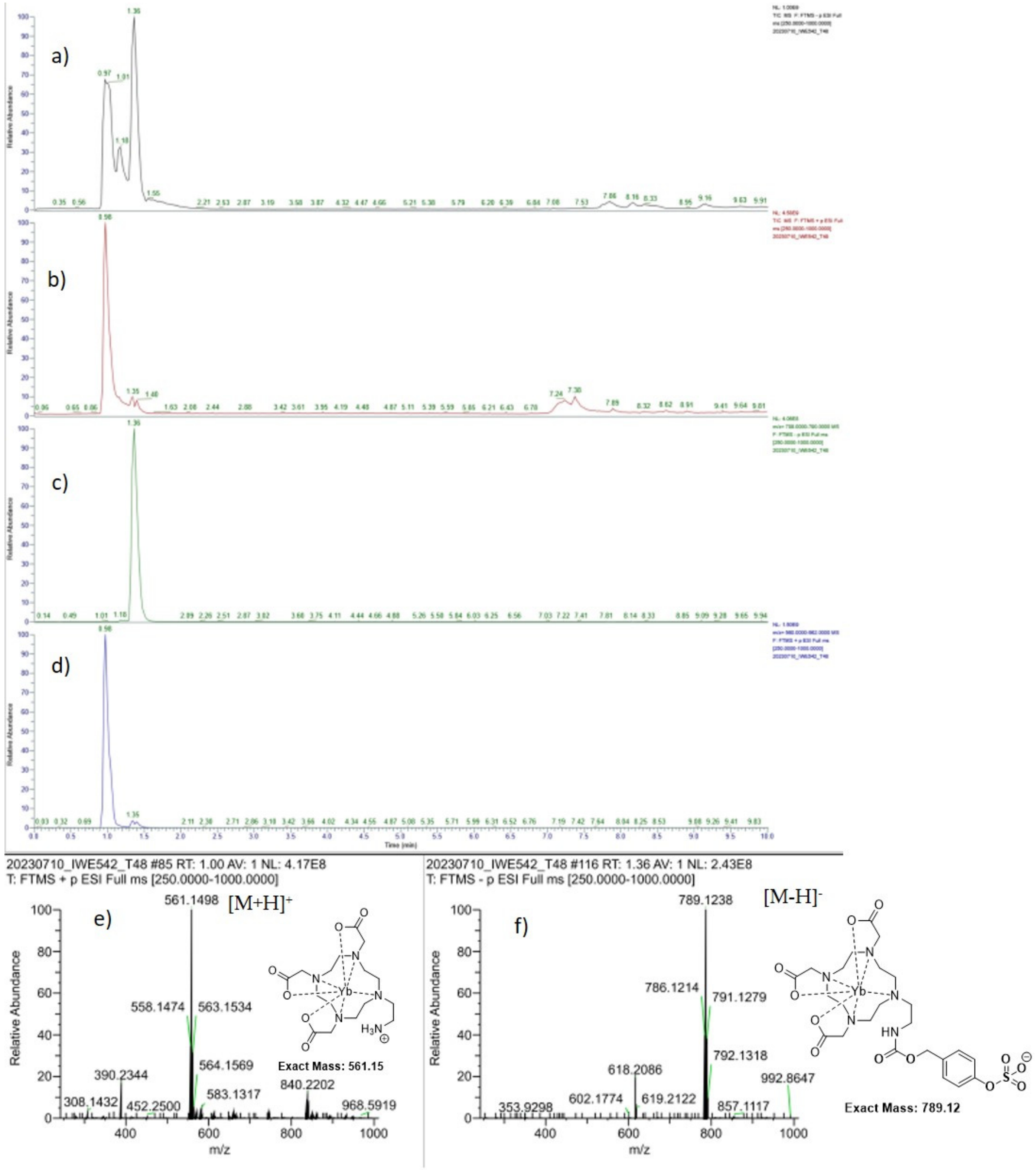

Figure S16. UPLC-MS analysis of enzyme reaction with **1-Yb** t = 24 h. (a) total ion current chromatogram negative mode, (b) total ion current chromatogram positive mode), (c) 778-790 mass trace, (d) 560-562 mass trace, (e) mass spectrum of the peak at R_t = 0.98 min. (f) mass spectrum of the peak at R_t = 1.36 min.

6. Determination of free Yb^{3+} and Gd^{3+} concentration:

A previously published assay was adapted for the determination of free metal concentration. [5] The concentration of free Yb^{+3} and Gd^{+3} was quantified by determination of the ratio of absorbance intensity at λ= 573 nm and λ = 433 nm of an Yb^{+3}-xylenol orange complex and Gd^{+3}-xylenol orange complex in ammonium acetate buffer (100 mM, pH 5.8, 0.60 mM xylenol Orange) using a microplate reader. The contrast agents were diluted with ammonium acetate buffer before analysis.

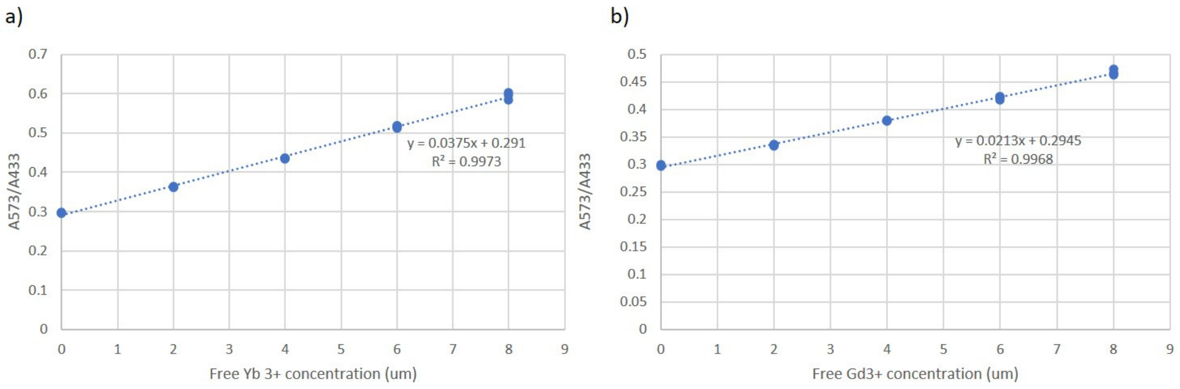

Figure S17. Quantification of free Yb^{III} and Gd^{III}. (a) Calibration curve showing the ratio of absorbance intensity at λ = 573 nm and λ = 433 nm for increasing Yb^{III} concentration in the presence of xylenol orange (0.60 mM). (b) Calibration curve showing the ratio of absorbance intensity at λ = 573 nm and λ = 433 nm for increasing Gd^{III} concentration in the presence of xylenol orange (0.60 mM).

For the analysis the samples of **1-Yb** (20 mM concentration from the Z-spectra measurement before and after enzymatic hydrolysis) and **1-Gd** (1 mM from the NMRD profiles measurements before and after enzymatic reaction) were diluted to a 20 µM concentration, the respectively free Yb^{+3} and Gd^{+3} was determined to be around 1-2% of each sample. All samples where measured in Duplo.

Table S10. Results of the determination of free metal concentration. Column 1: sample name, column 2: ratio of absorbance intensity at λ = 573 nm and λ = 433 nm. Column 3: calculated free lanthanide concentration in µM. Column 4: recalculated concentration of free metal taking into account the dilution of the sample. Column 5: % of free lanthanide in the sample.

Sample	*A573/A433*	*µM*	*recalculated with dilution factor µM*	*Free Ln^{3+} %*
1-Yb	0.30	0.27	272	1.36
1-Yb	0.30	0.37	369	1.85
1-Yb after enzyme	0.30	0.36	363	1.82
1-Yb after enzyme	0.31	0.42	422	2.11
1-Gd	0.30	0.27	13.6	1.36
1-Gd	0.30	0.28	13.9	1.39
1-Gd after enzyme	0.31	0.53	26.7	2.67
1-Gd after enzyme	0.30	0.36	18.2	1.82
1-Gd after more enzyme	0.31	0.55	27.4	2.74
1-Gd after more enzyme	0.30	0.44	21.9	2.19

7. Stability test in human plasma-like medium

Initial stability test of compound **1-Gd** was performed in human plasma-like medium (HPLM), and the samples were followed by UPLC-MS. For the analysis three samples of **1-Gd** (1 mM, in 0.5 mL HPLM) were injected in the LCMS before and after 24 h at 37 °C. Analyzed with UPLC-MS program 3. Results are shown in table 11 and visualized in Figure S18.

Table S11. LCMS analysis of **1-Gd** in HPLM. Peak Areas of **1-Yb** for time point t = 0 and t = 24 h at 37 °C.

time points (h)	*Area 1-Gd*	*time points (h)*	*Area 1-Gd*
0_1	23146674	24_1	23288452
0_2	24217270	24_2	22372496
0_3	24025010	24_3	23860014
SD	570761.7	*SD*	750374.2
Average	23796318	*Average*	23173654

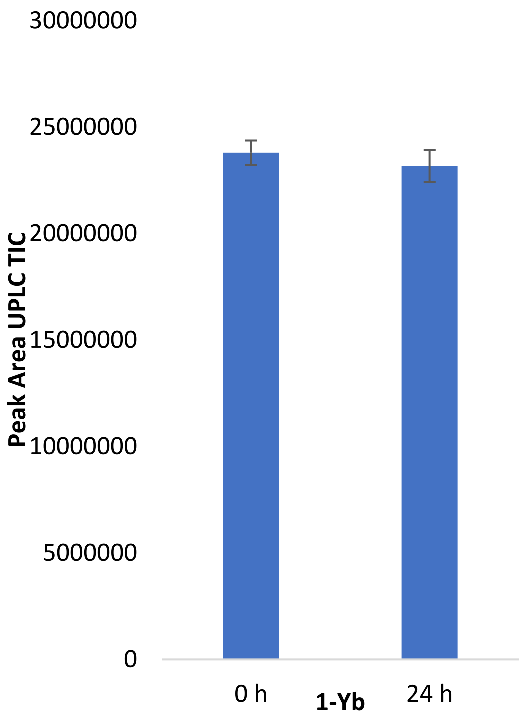

Figure S18. UPLC-MS analysis of **1-Gd** (1.0 mM) in HPLM at 37 °C followed over 24 h. Error bars represent the standard deviation.

8. NMRs

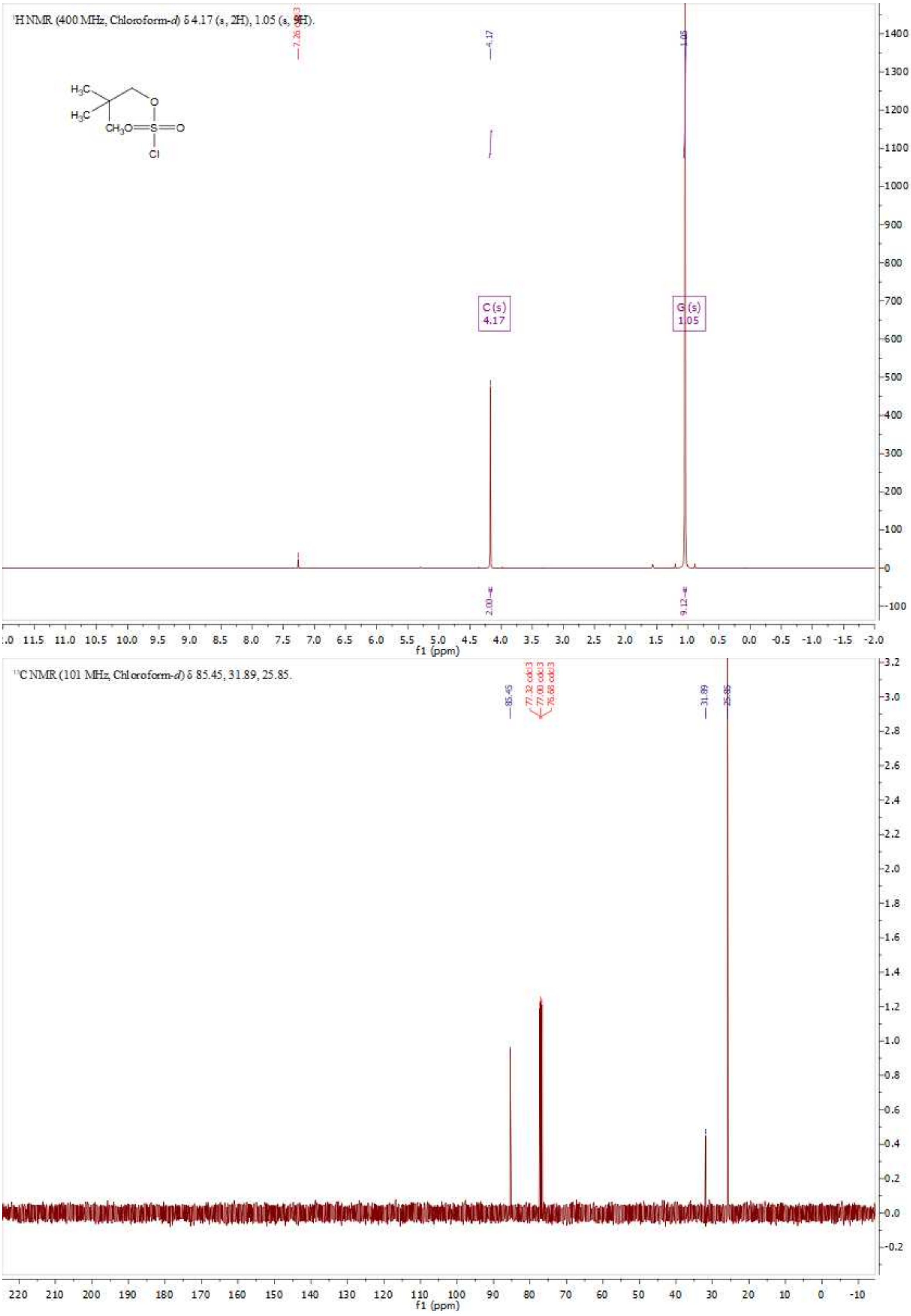

Figure S19. ^{1}H NMR and ^{13}C NMR of Neopentyl chlorosulfate **(4)**.

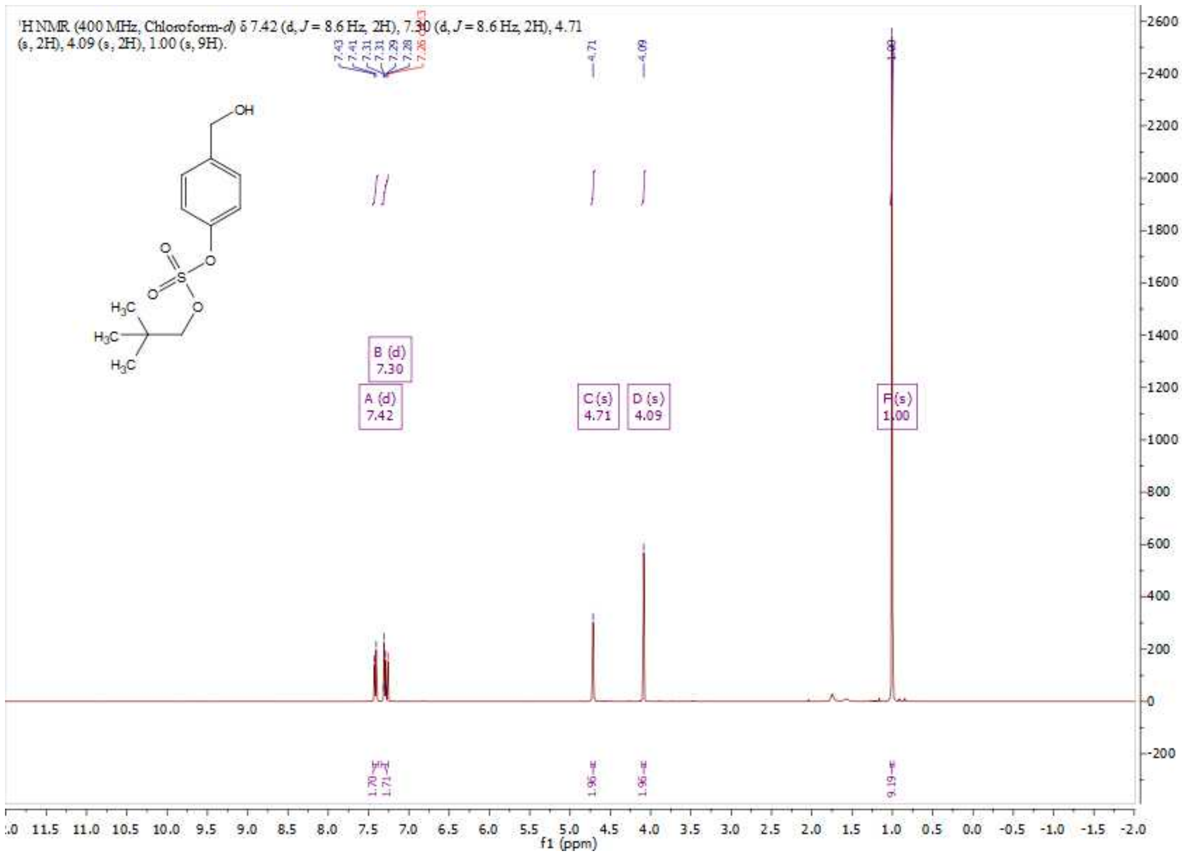

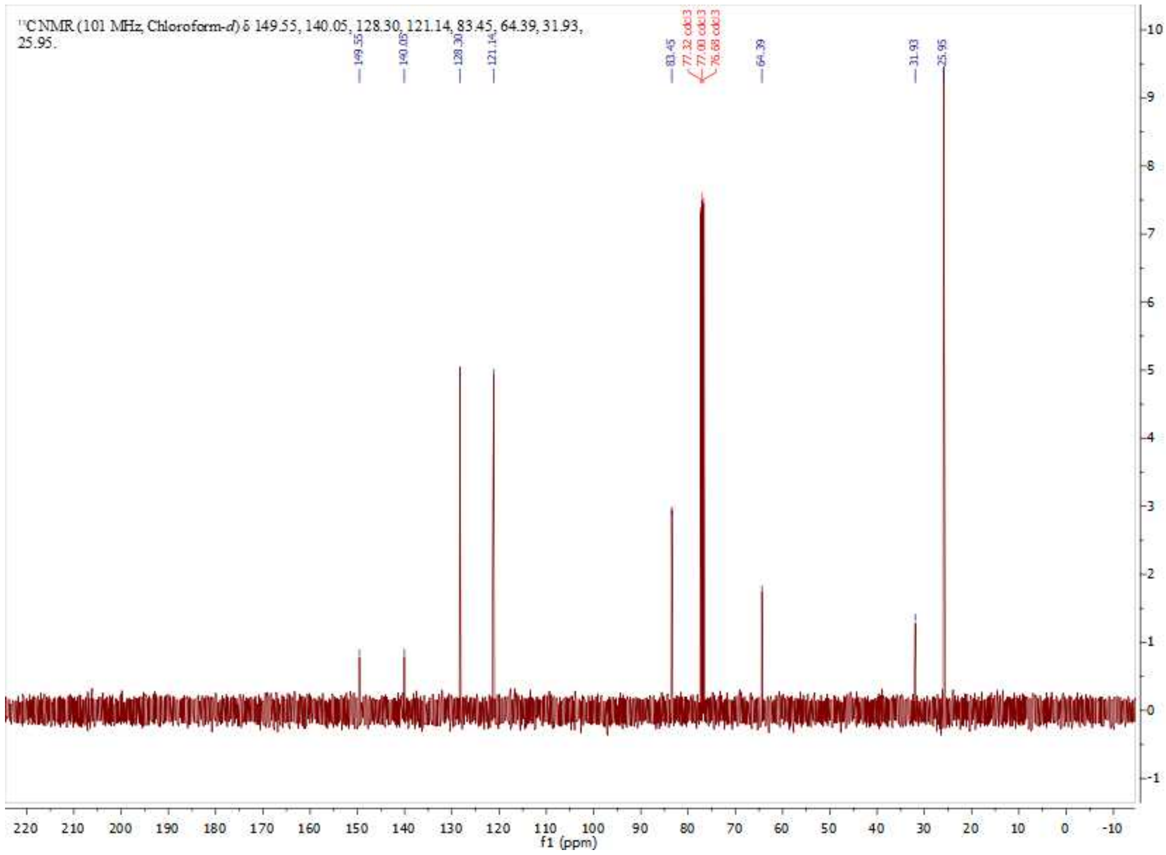

Figure S20. ^{1}H NMR and ^{13}C NMR of 4-(Hydroxymethyl)phenyl neopentyl sulfate **(5).**

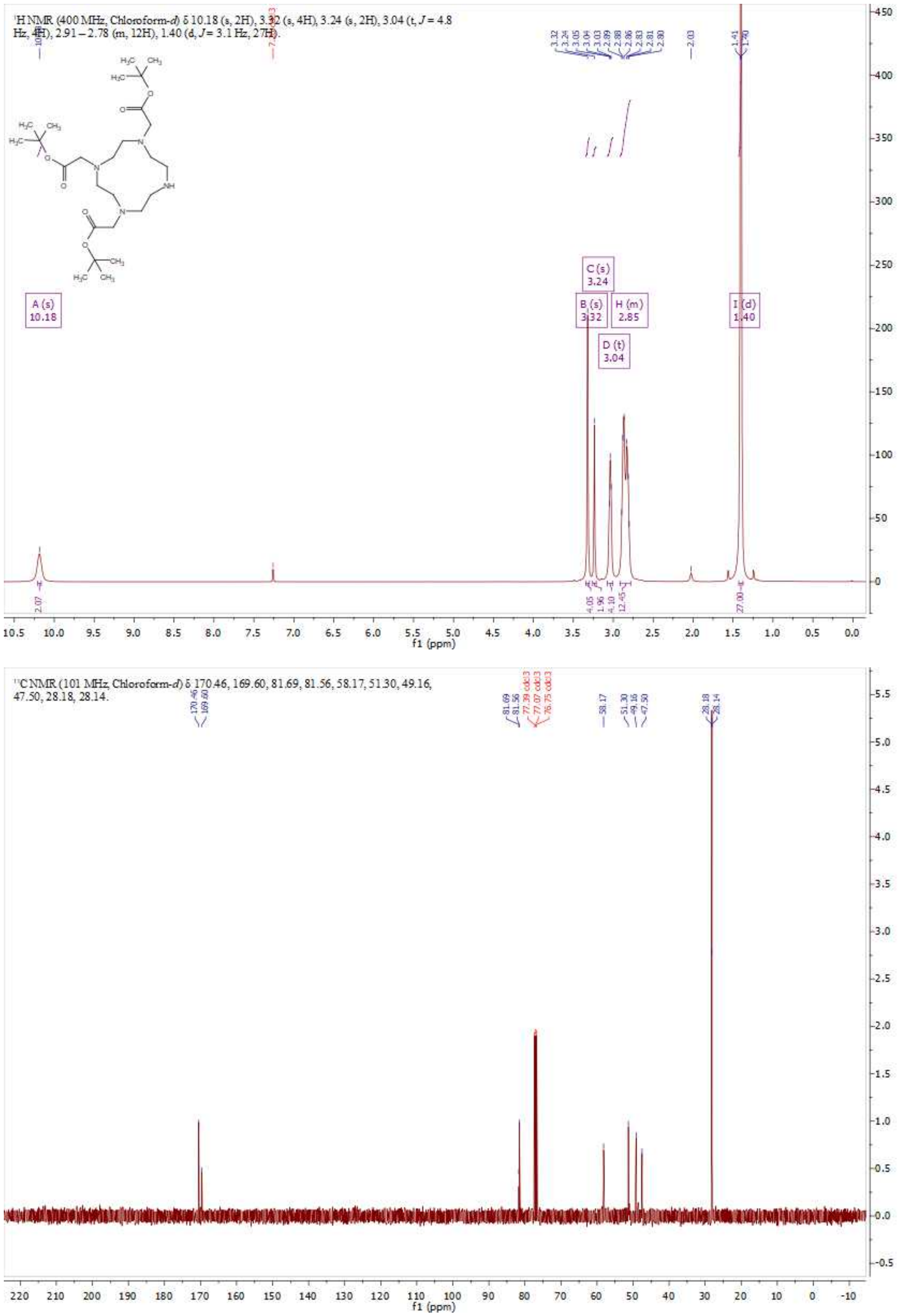

Figure S21. ^{1}H NMR and ^{13}C NMR of Tri-tert-butyl 2,2',2''-(1,4,7,10-tetraazacyclododecane-1,4,7-triyl)triacetate **(9)**.

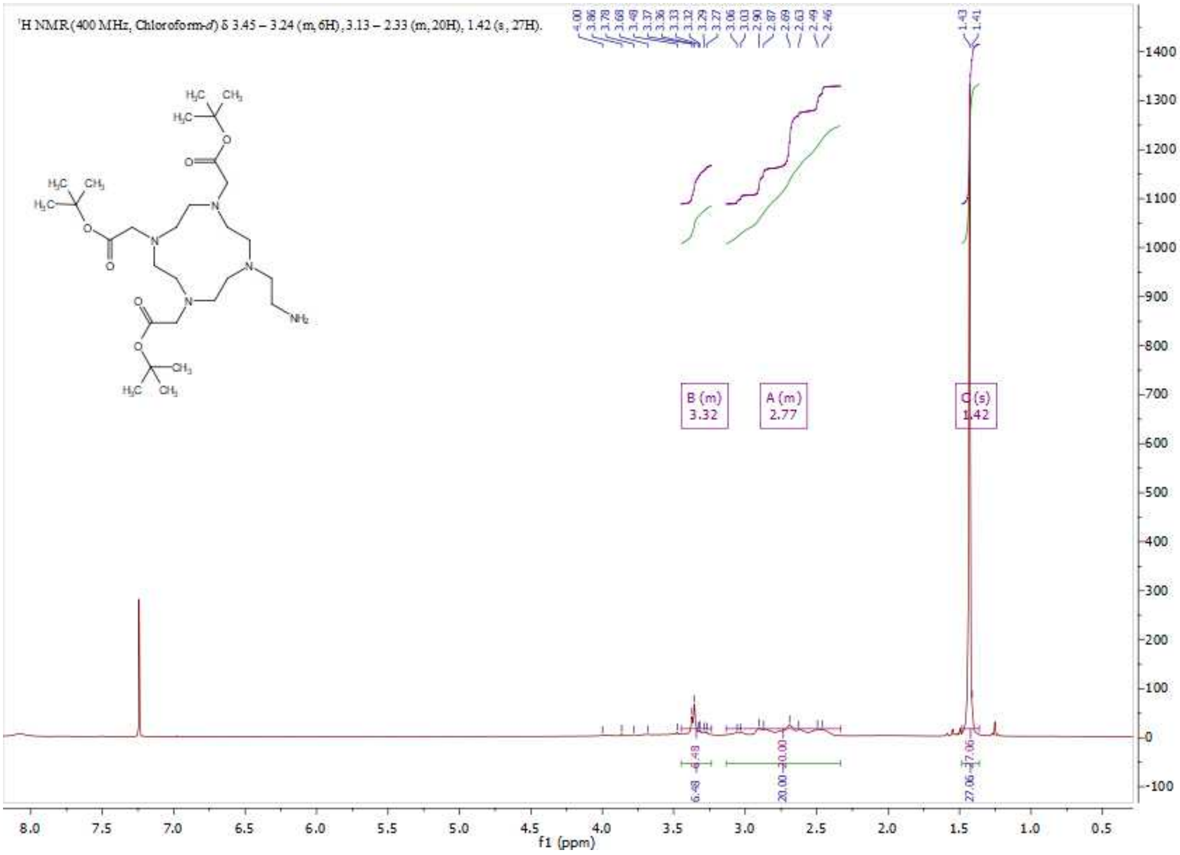

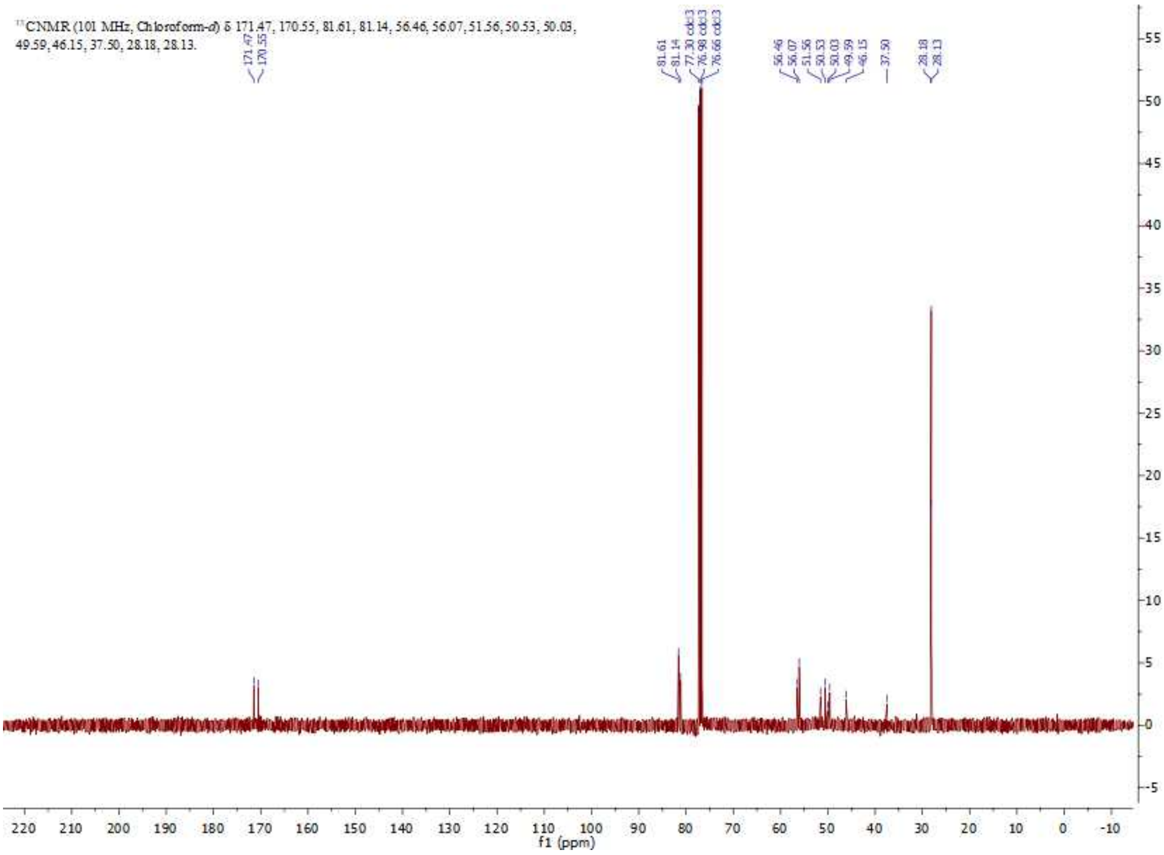

Figure S22. ^{1}H NMR and ^{13}C NMR of Tri-tert-butyl 2,2',2''-(10-(2-aminoethyl)-1,4,7,10-tetraazacyclododecane-1,4,7-triyl)triacetate **(6)**.

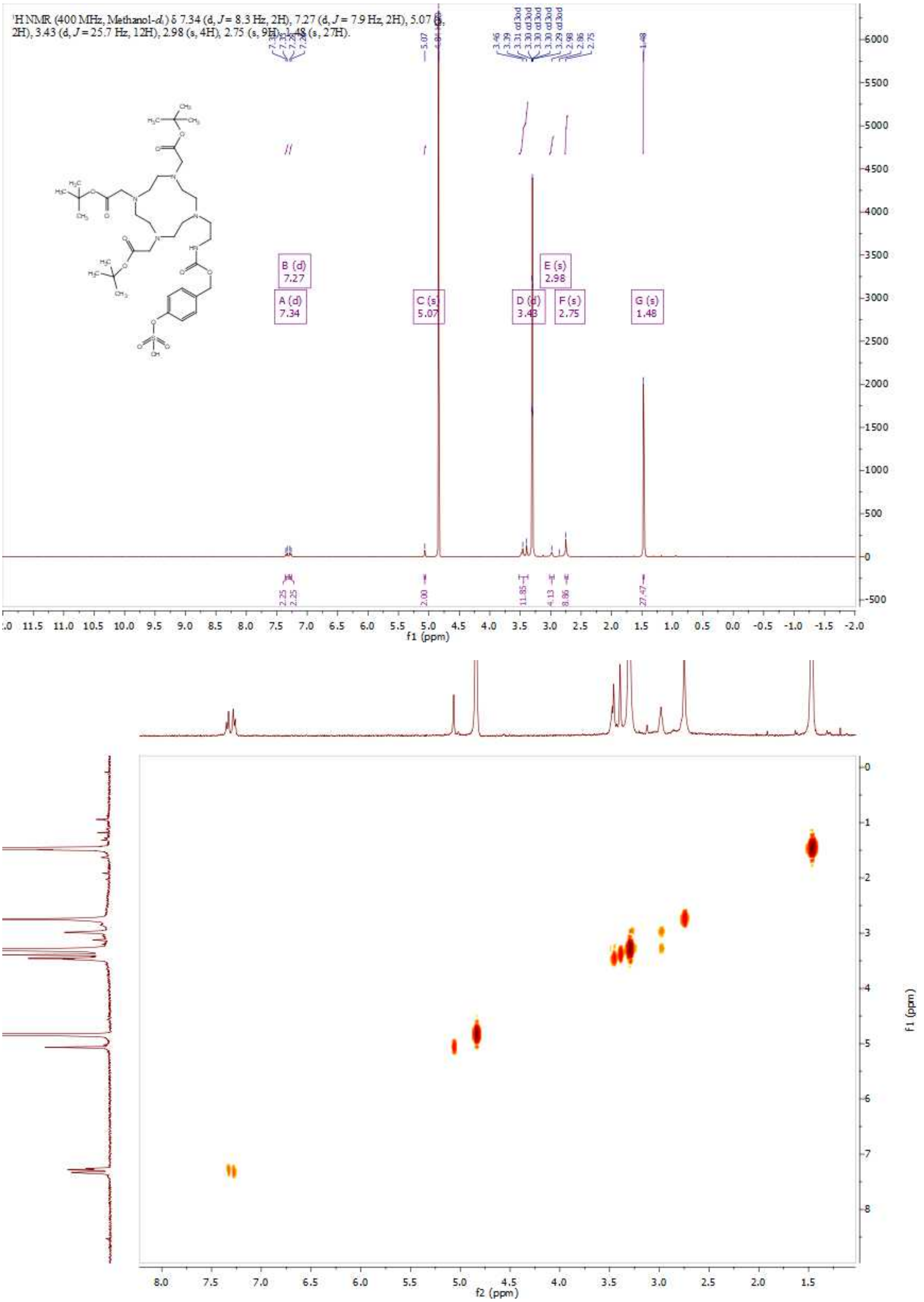

Figure S23. ^{1}H NMR and COSY of 4-((((2-(4,10-bis(2-(tert-butoxy)-2-oxoethyl)-7-(2-(neopentyloxy)-2-oxoethyl)-1,4,7,10-tetraazacyclododecan-1-yl)ethyl)carbamoyl)oxy)methyl)phenyl sulfate **(8)**.

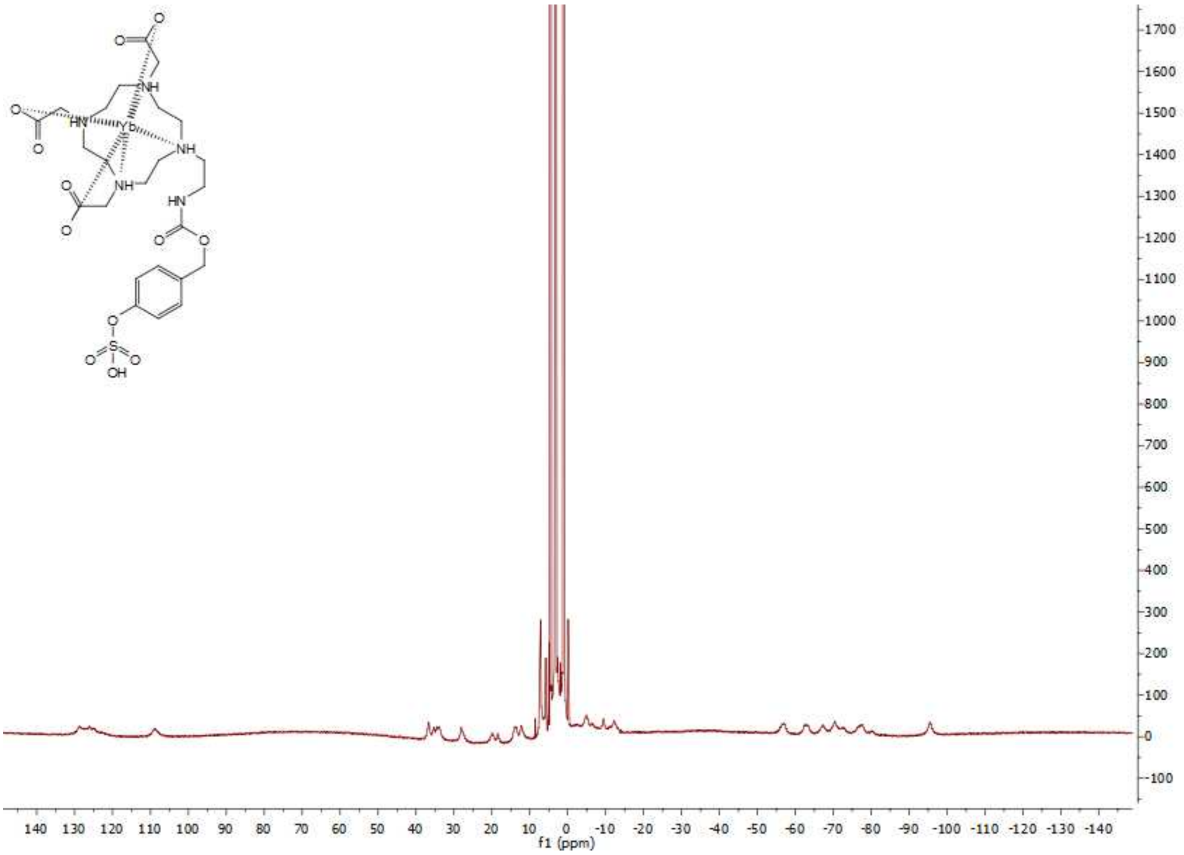

Figure S24. ^{1}H NMR of **1-Yb** taking in TEAA buffer solution.

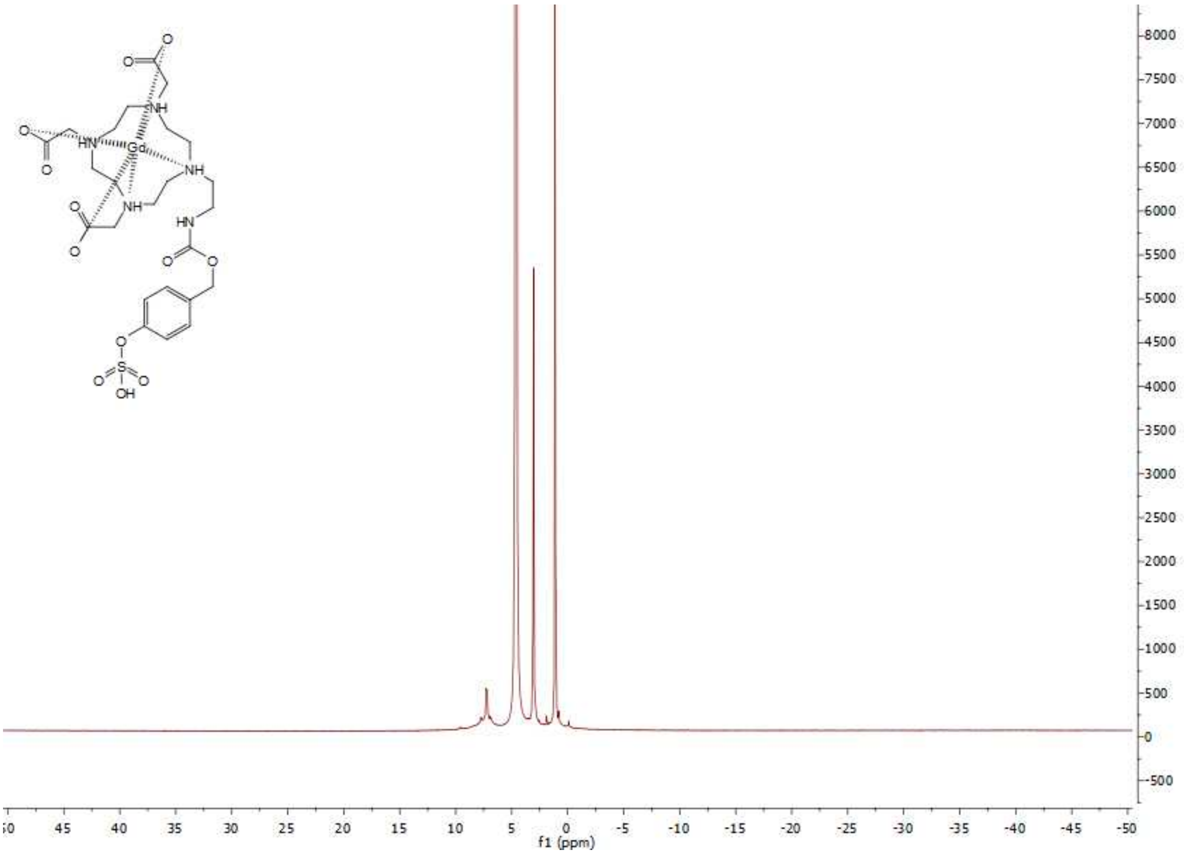

Figure S25. ^{1}H NMR of **1-Gd**, taking in TEAA buffer solution.

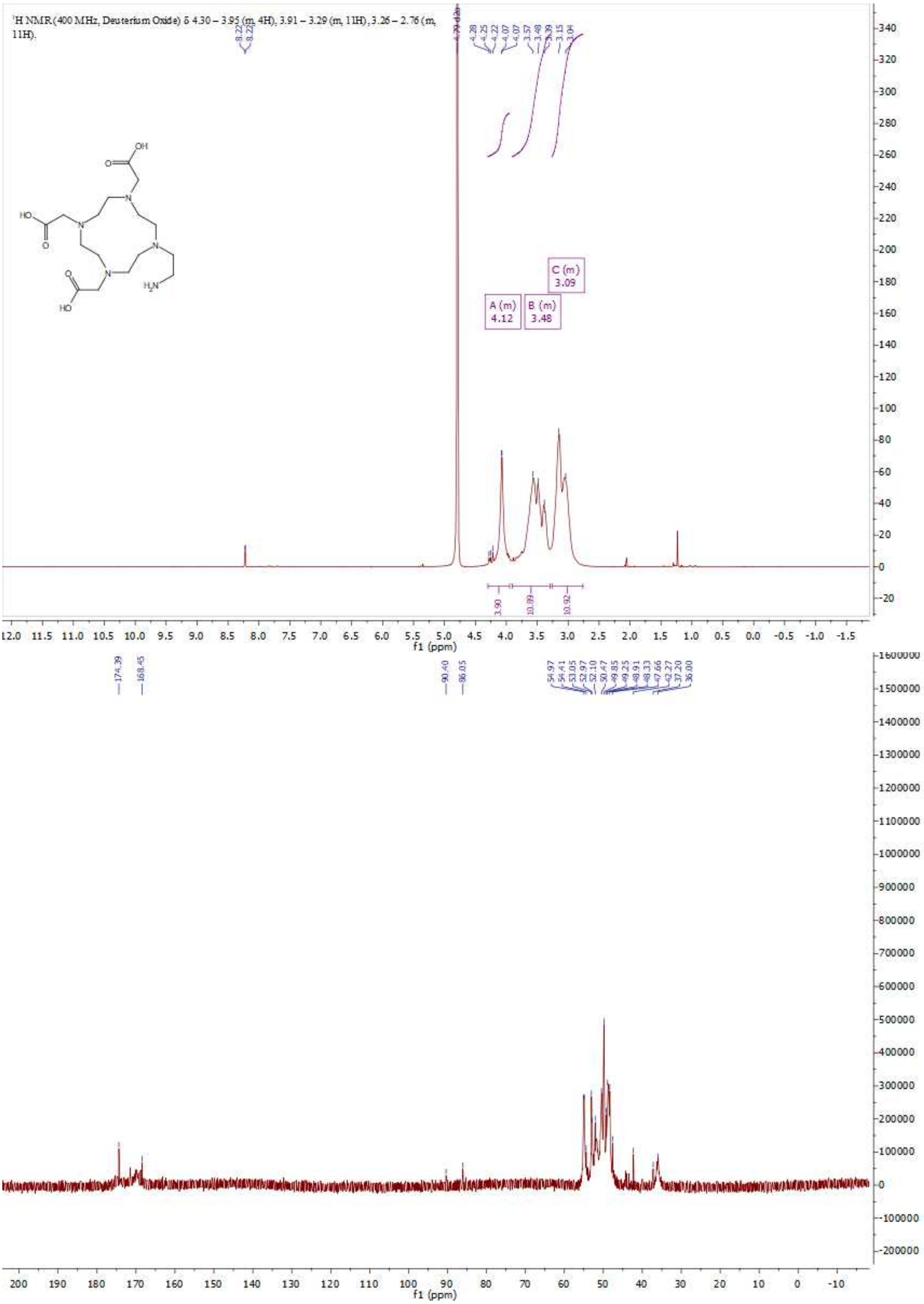

Figure S26. ^{1}H NMR and ^{13}C NMR of 2,2',2''-(10-(2-ammonioethyl)-1,4,7,10-tetraazacyclododecane-1,4,7-triyl)triacetate (**3**).

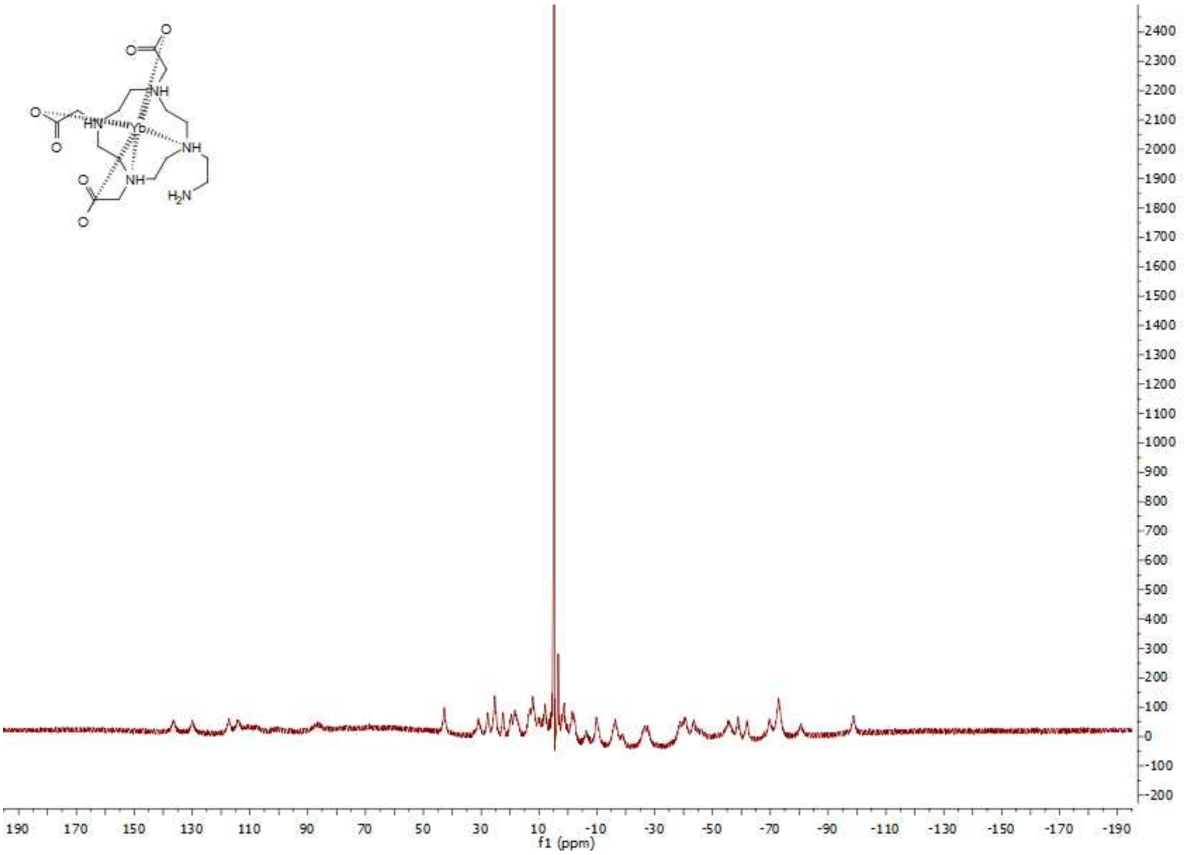

Figure S27. ^{1}H NMR of **3-Yb**.

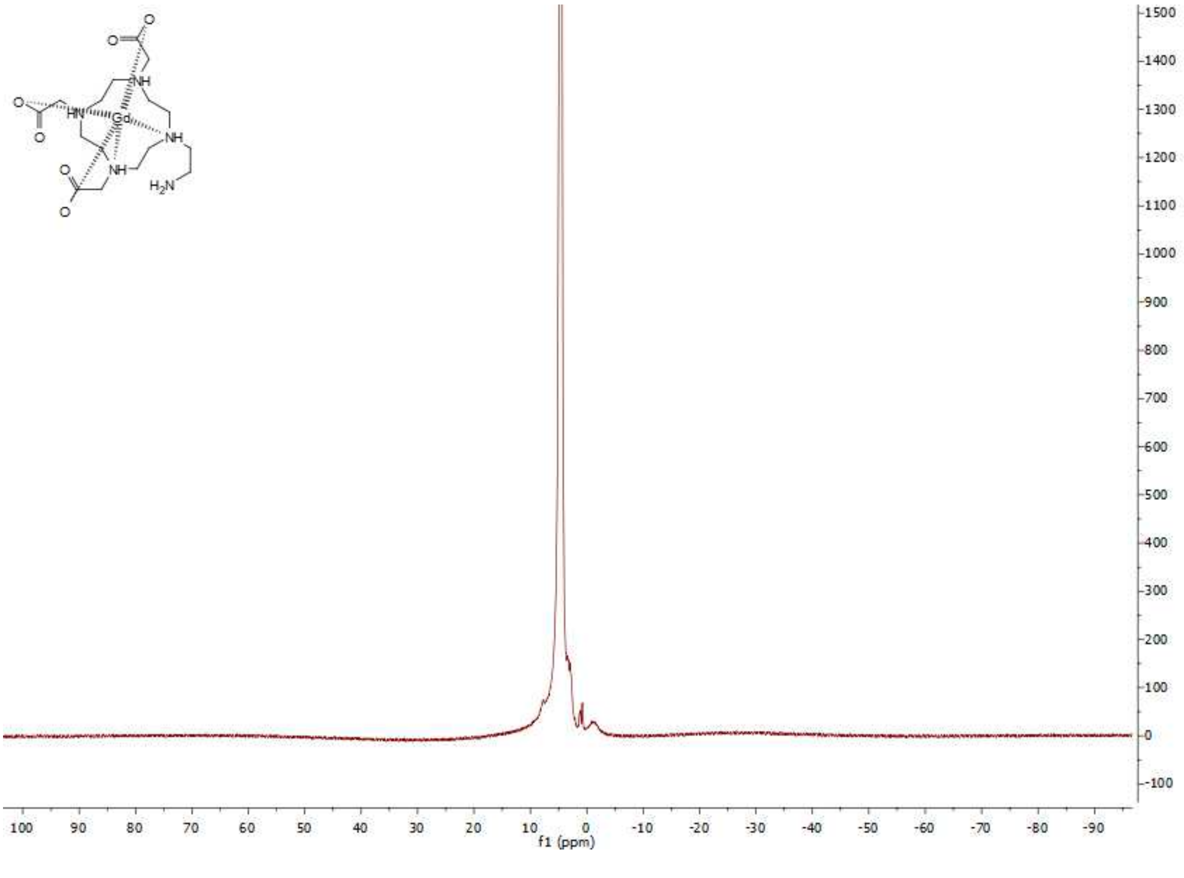

Figure S28. ^{1}H NMR of **3-Gd**.

9. HRMS

Compound 9

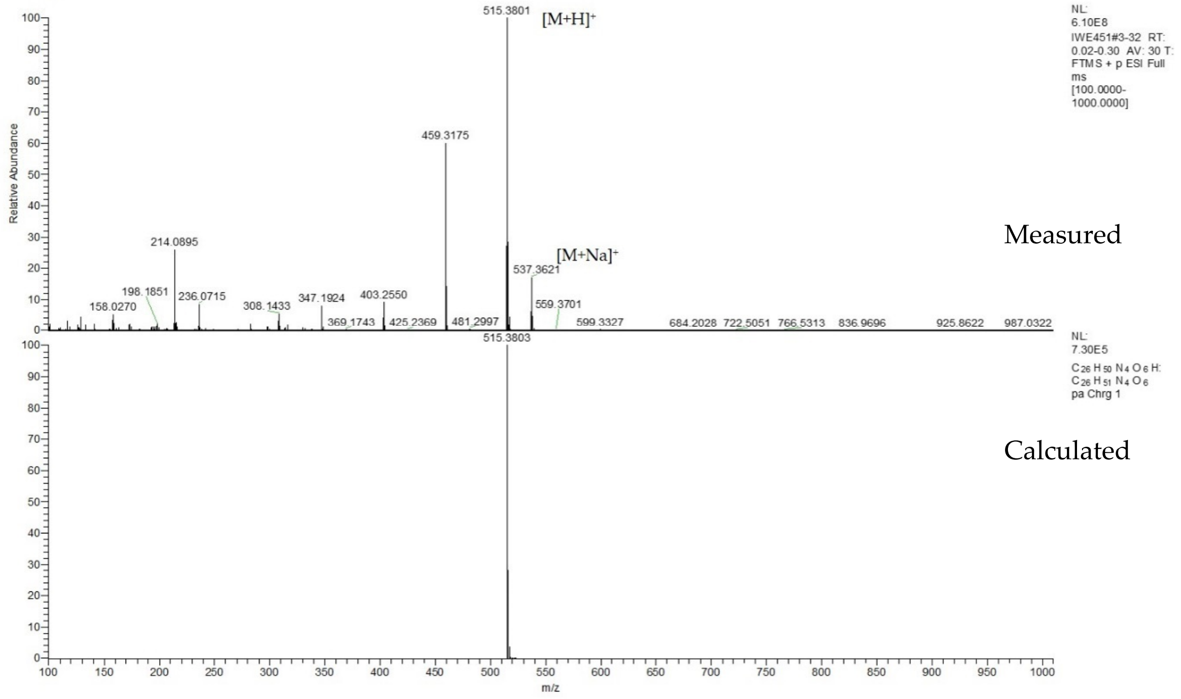

Compound 9 Zoomed in ESI positive (M+H)

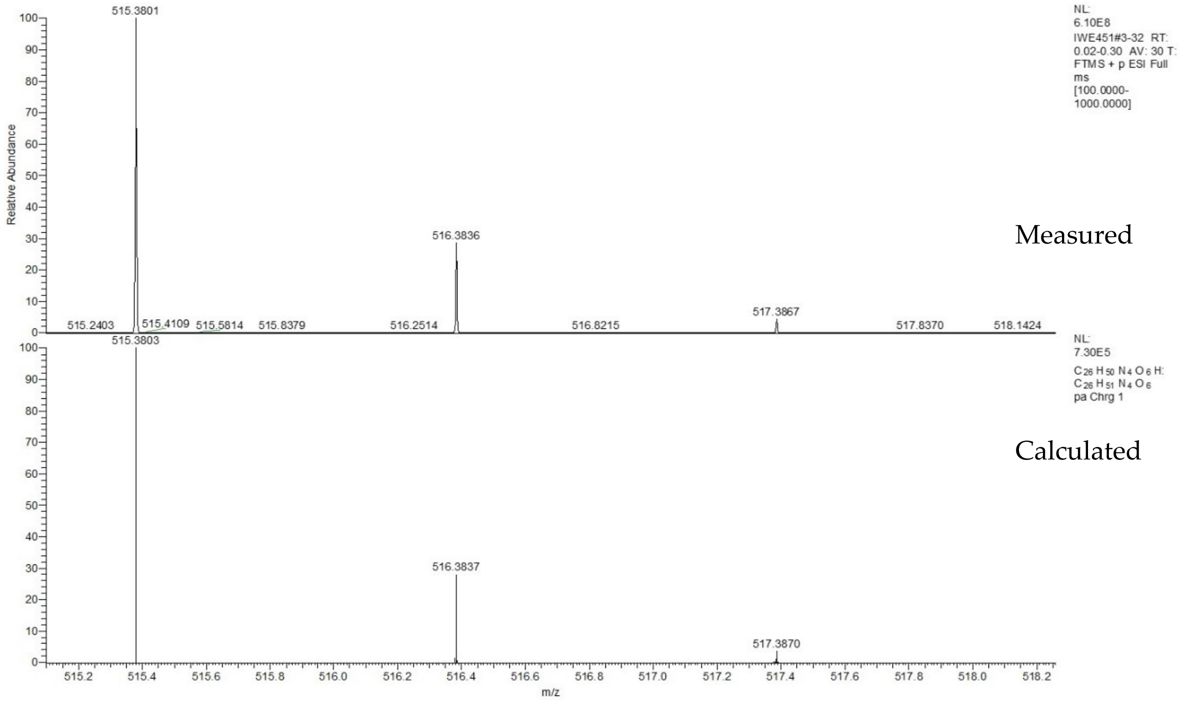

Figure S29. HRSM of compound **9**, HRMS (ESI+) calc. for $[M+H]^+$ ($C_{26}H_{51}N_4O_6$): 515.3803, found: 515.3801.

Compound 6

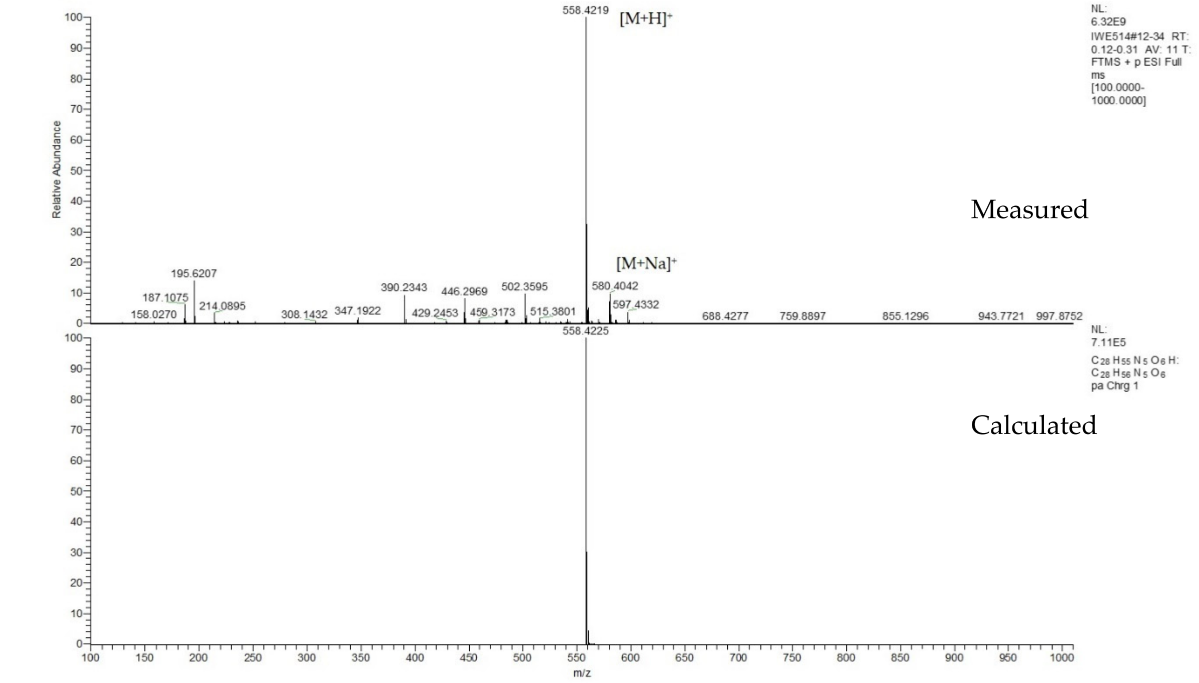

Compound 6 Zoomed in ESI positive (M+H)

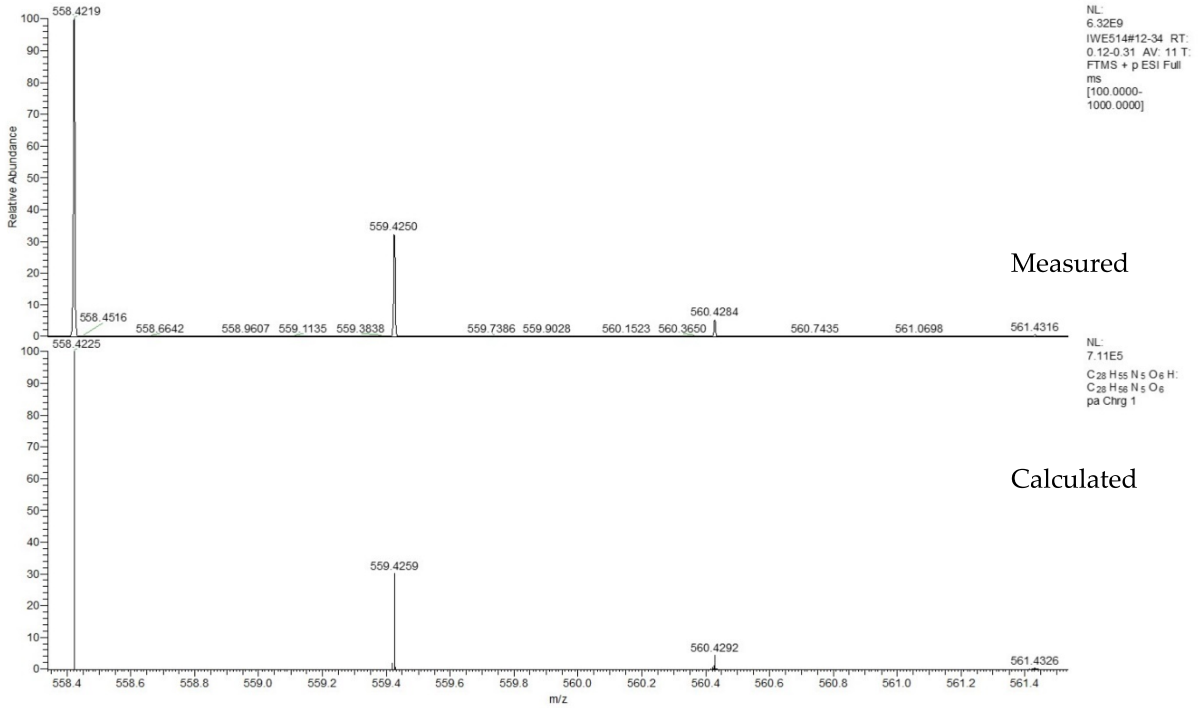

Figure S30. HRSM of compound **6**, HRMS (ESI+) calc. for [M+H]+ ($C_{28}H_{56}N_5O_6^+$): 558.4225, found: 558.4219.

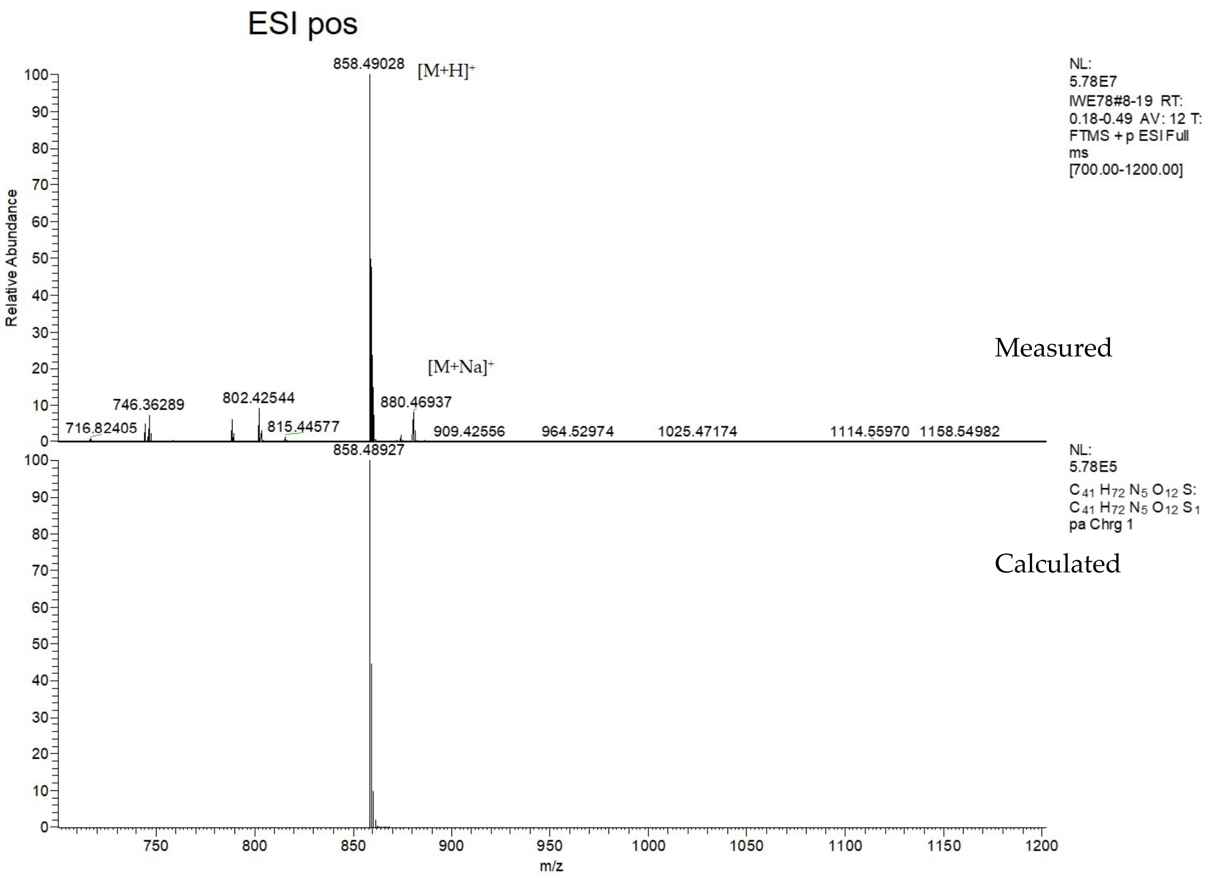

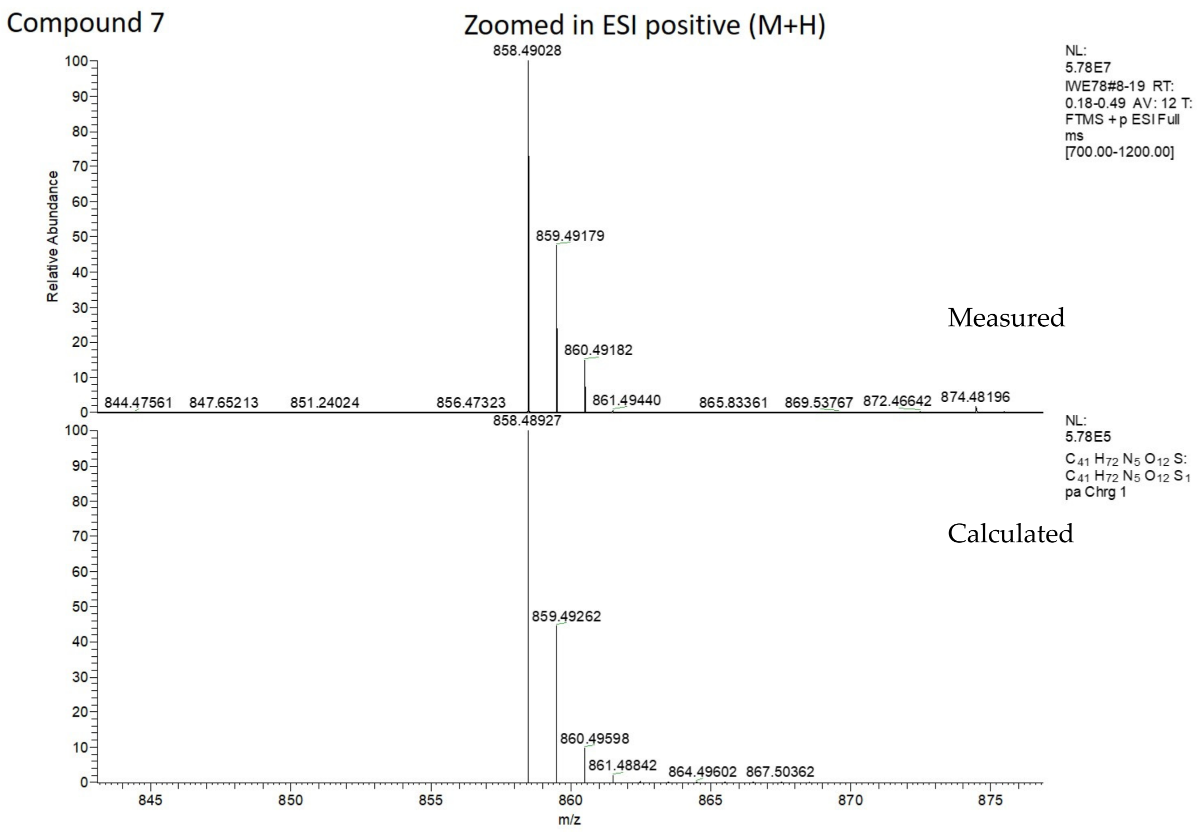

Figure S31. HRSM of compound **7**, HRMS (ESI+) calc. for $[M+H]^+$ ($C_{41}H_{72}N_5O_{12}S^+$): 858.4893 found: 858.4903.

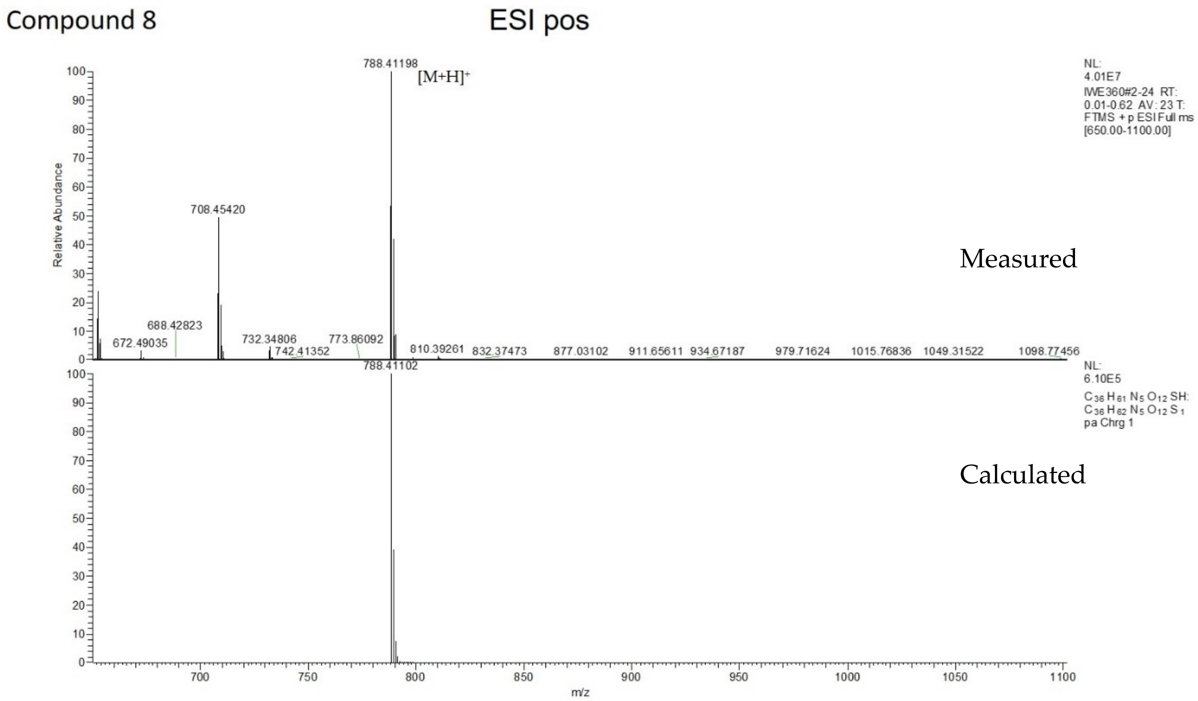

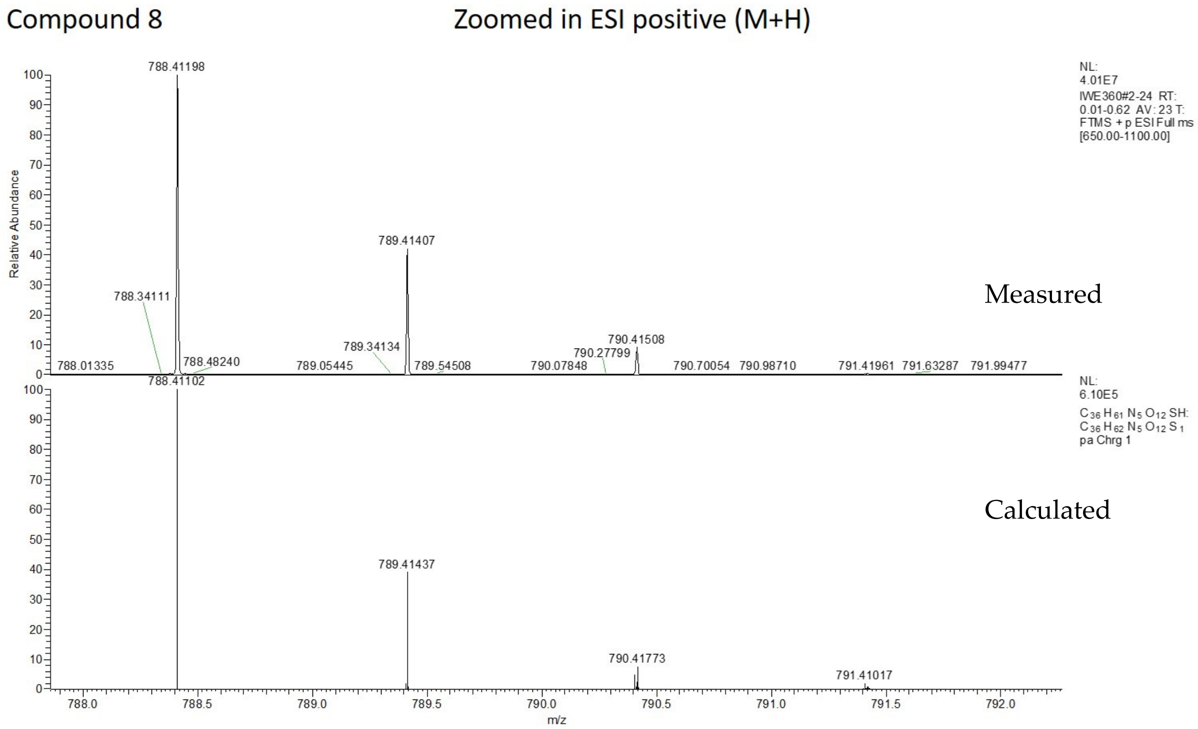

Figure S32. HRSM of compound **8**, HRMS (ESI+) calc. for $[M+H]^+$ ($C_{36}H_{62}N_5O_{12}S^+$): 788.4110 found: 788.4119.

Compound 1 ESI pos

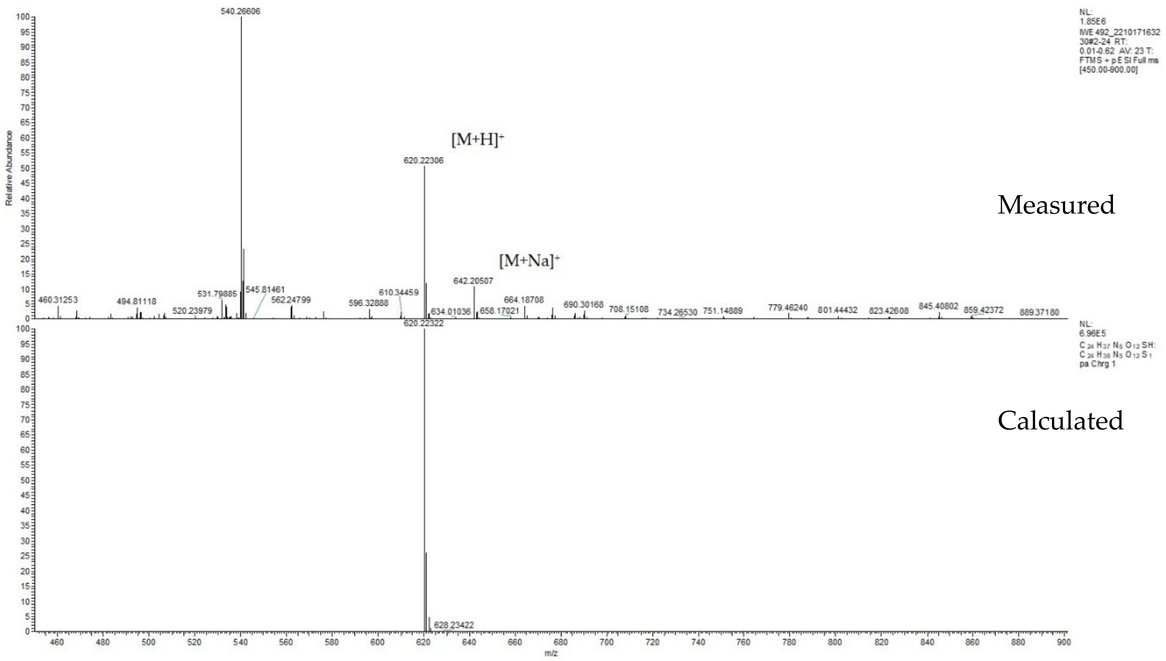

Compound 1 Zoomed in ESI positive (M+H)

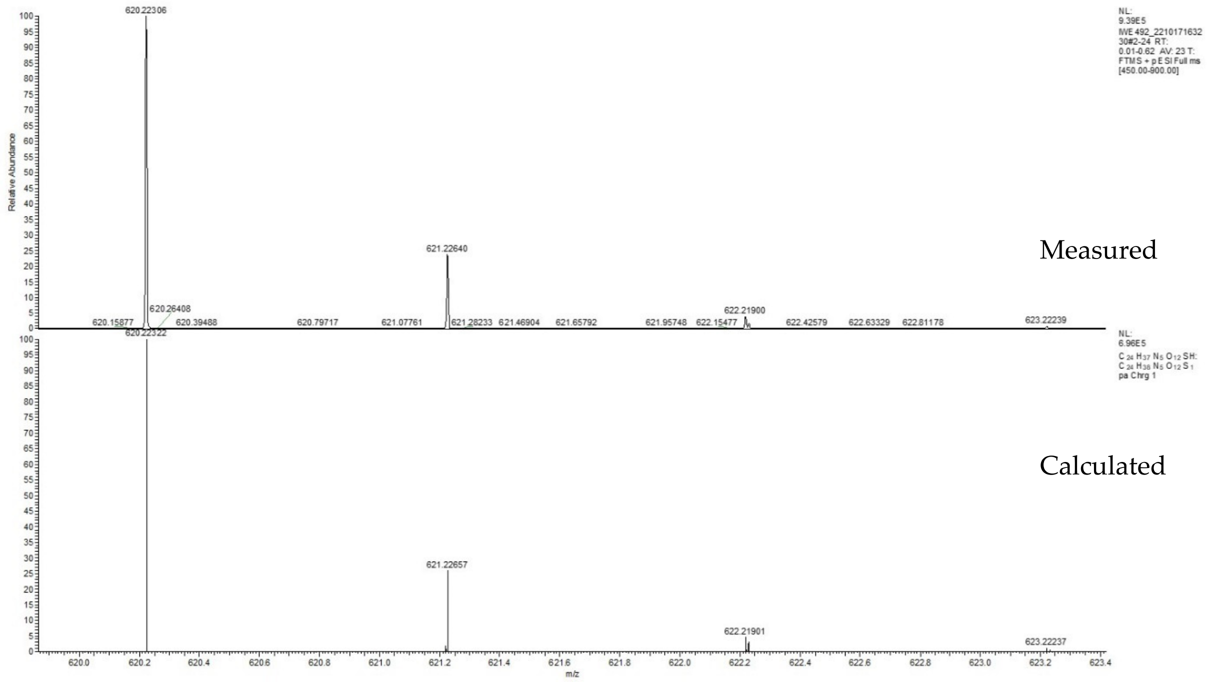

Figure S33. HRSM of compound **1**, HRMS (ESI+) calc. for [M+H]+ ($C_{24}H_{38}N_5O_{12}S^+$): 620.2232 found: 620.2230.

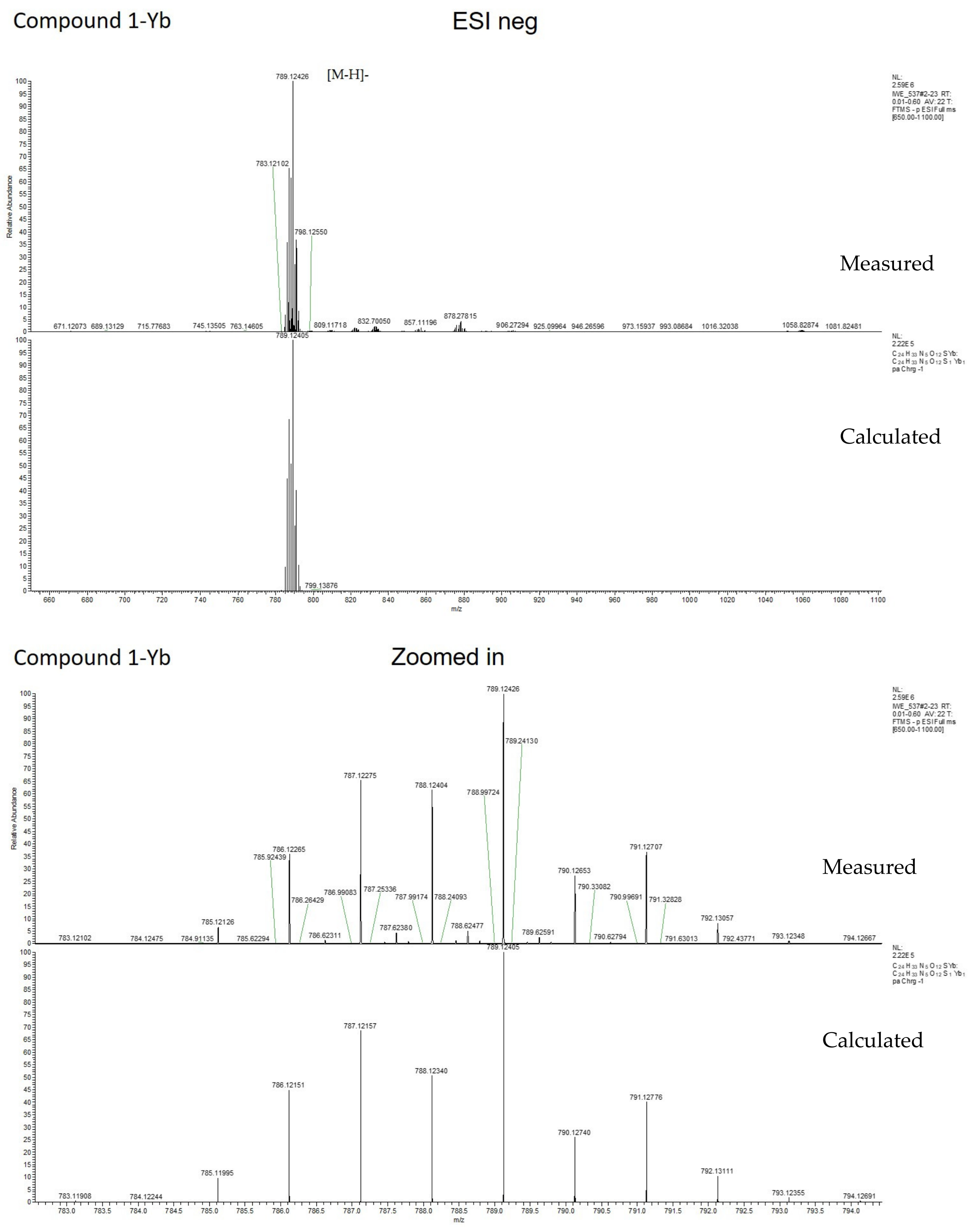

Figure S34. HRSM of compound **1-Yb**, HRMS (ESI-) calc. for [M-H]$^-$ ($C_{24}H_{33}N_5O_{12}SYb^-$): 789.1240, found: 789.1242.

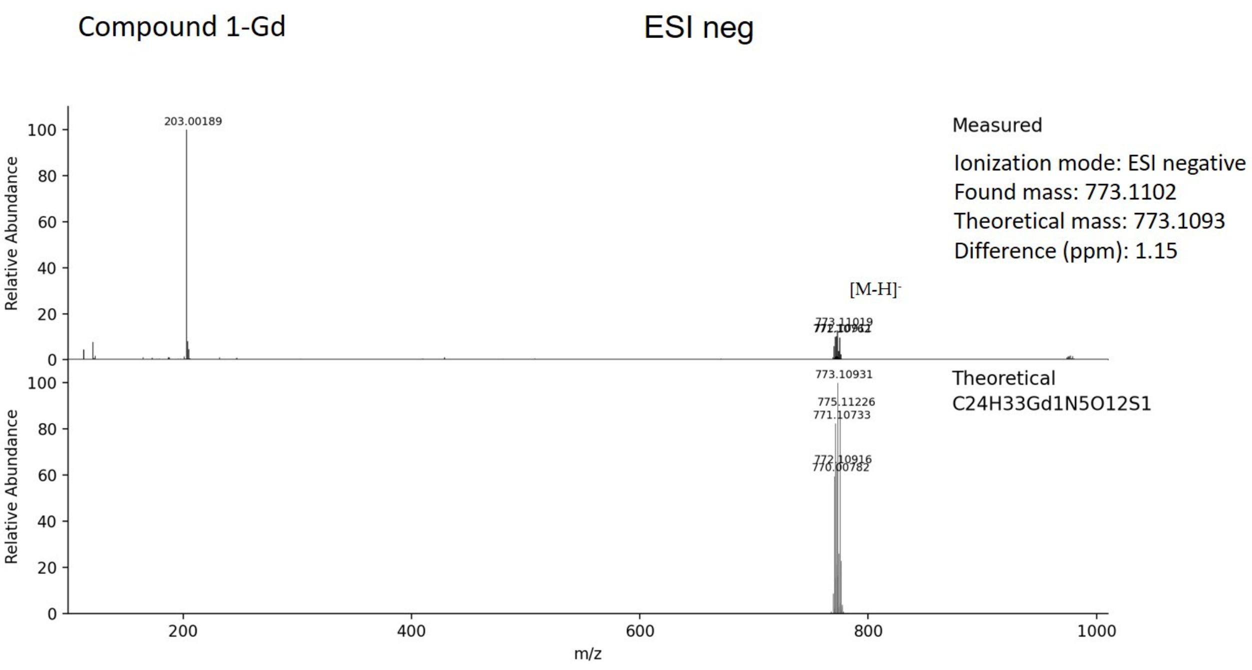

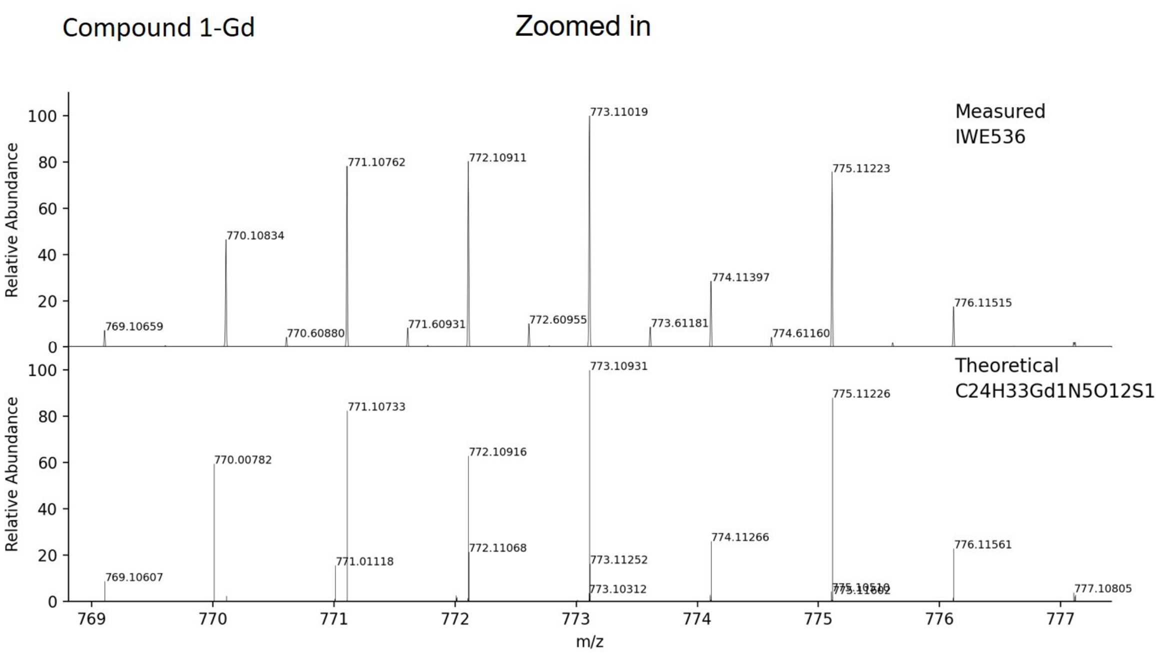

Figure S35. HRSM of **1-Gd**, HRMS (ESI-) calc. for [M-H]$^-$ ($C_{24}H_{33}N_5O_{12}SGd^-$): Exact Mass: 773.1093, found: 773.1102.

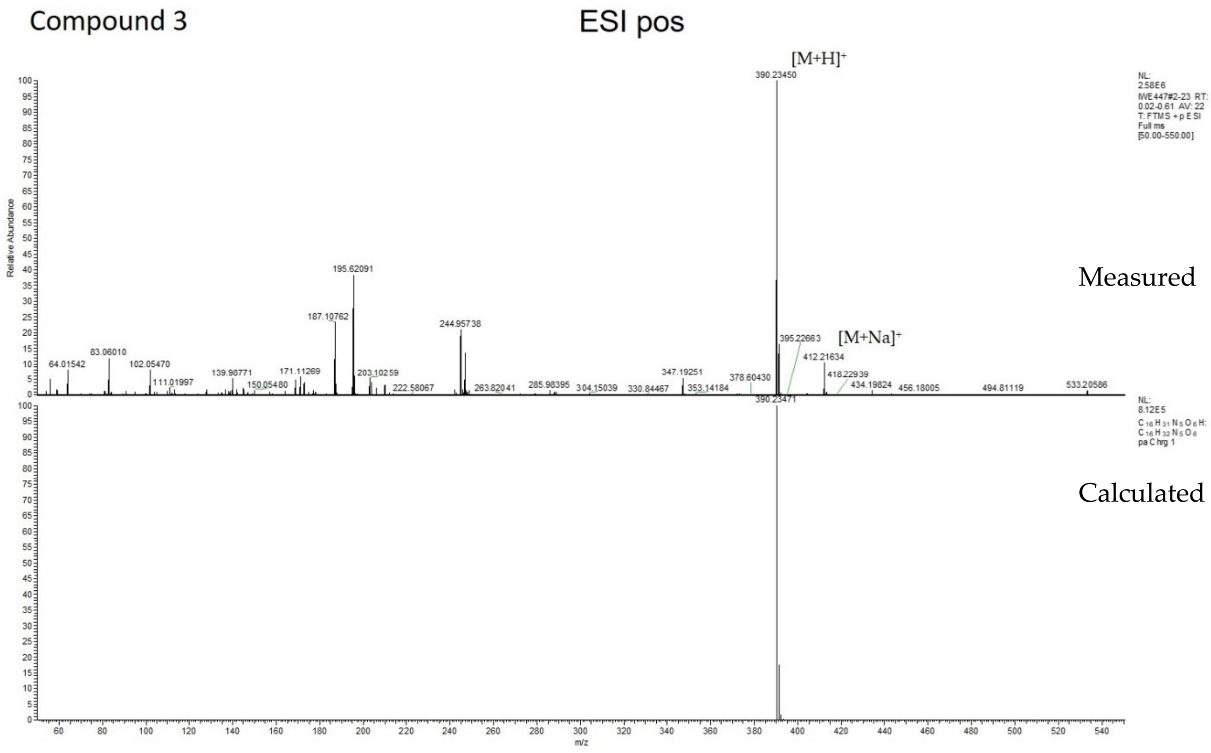

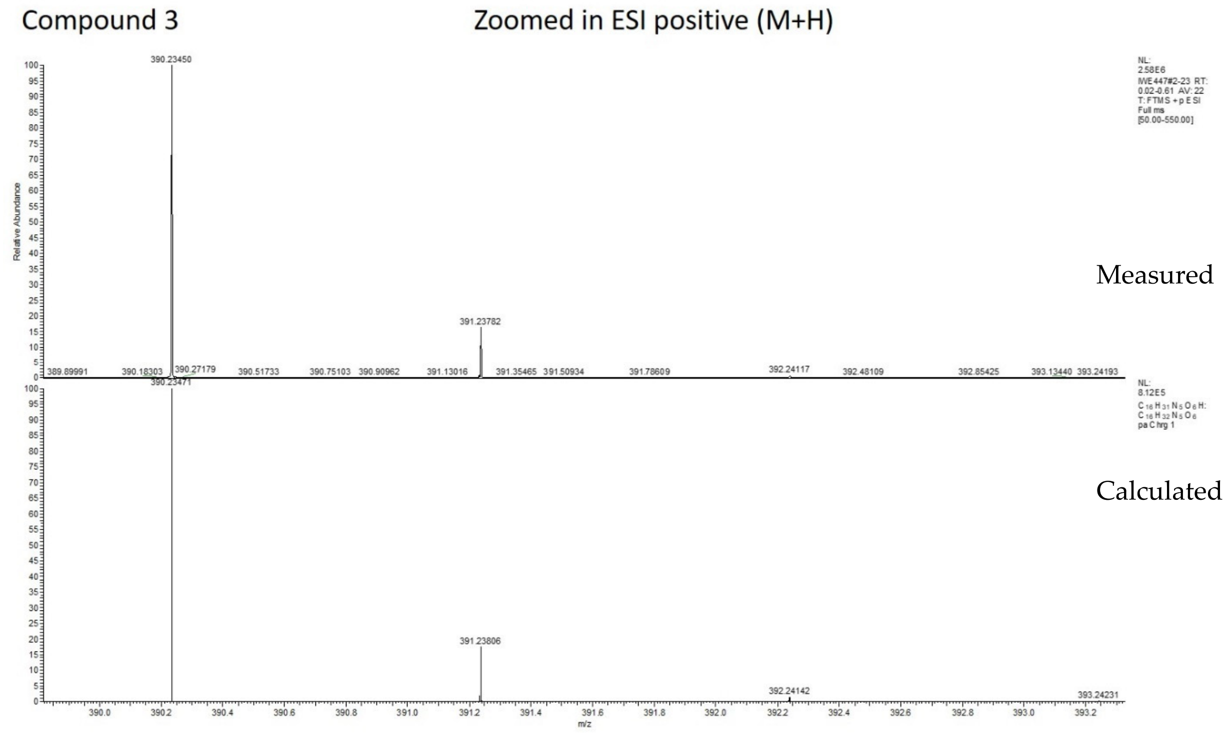

Figure S36. HRSM of **3**, HRMS (ESI+) calc. for [M+H]+ ($C_{16}H_{32}N_5O_6^+$): 390.2347, found: 390.2345.

Compound 3-Yb

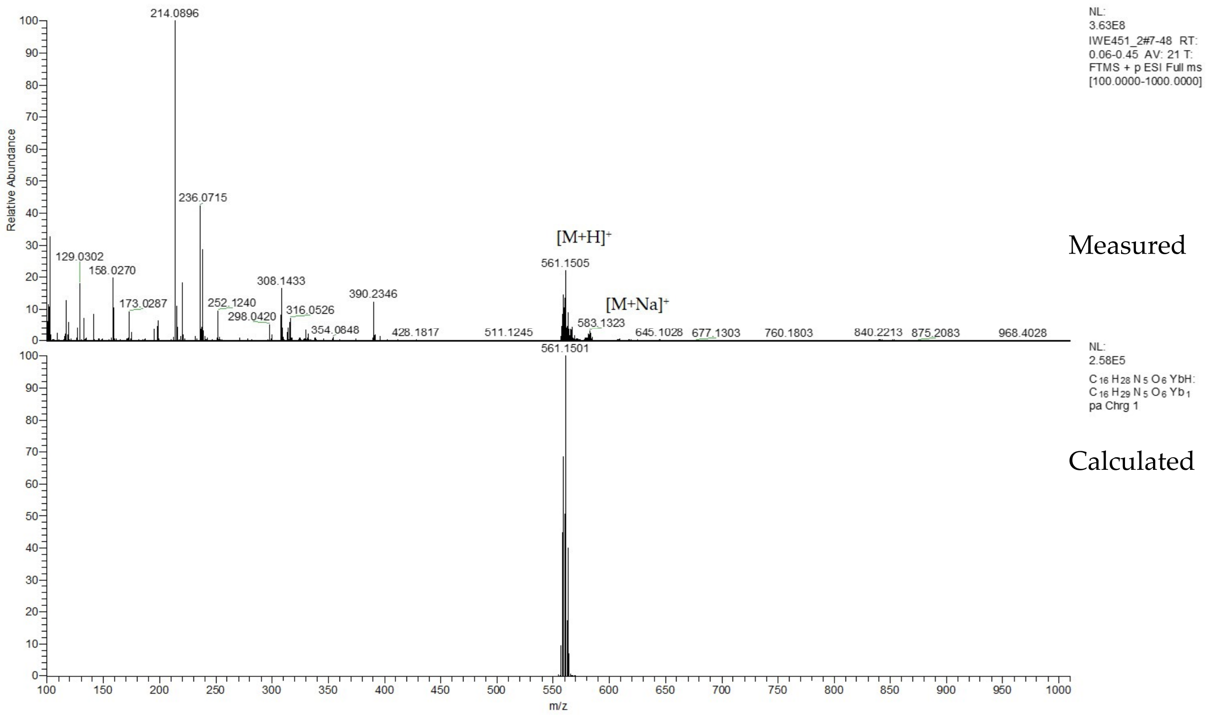

Compound: 3-Yb

Zoomed in ESI positive (M+H)

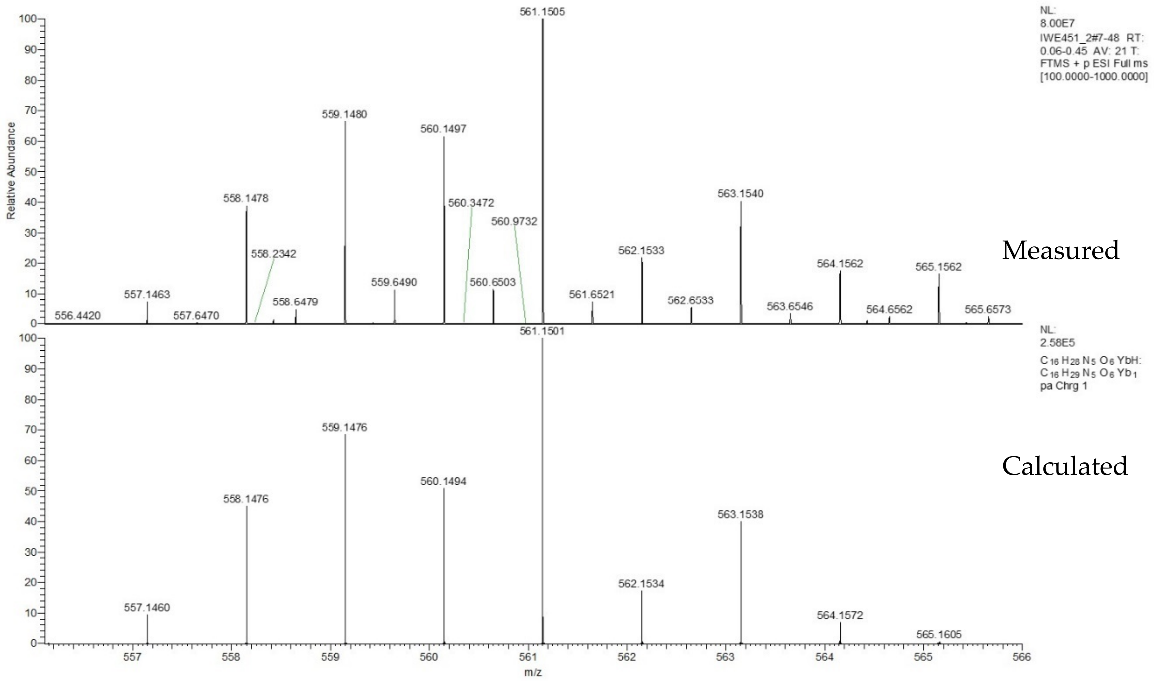

Figure S37. HRSM of **3-Yb**, HRMS (ESI+) calc. for [M+H]+ ($C_{16}H_{29}N_5O_6Yb^+$): 561.1500, found:561.1505.

Compound 3-Gd

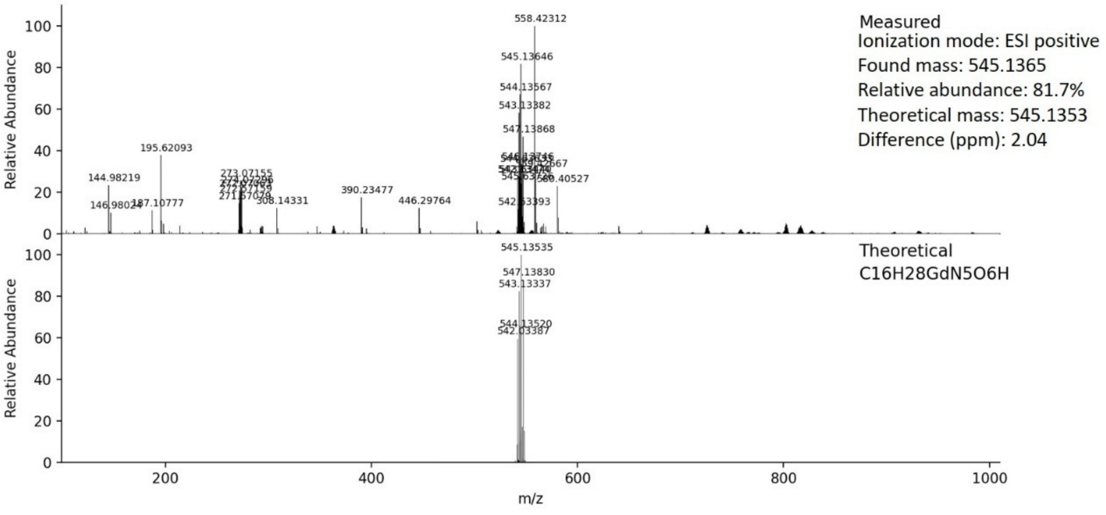

Compound: 3-Gd Zoomed in ESI positive (M+H)

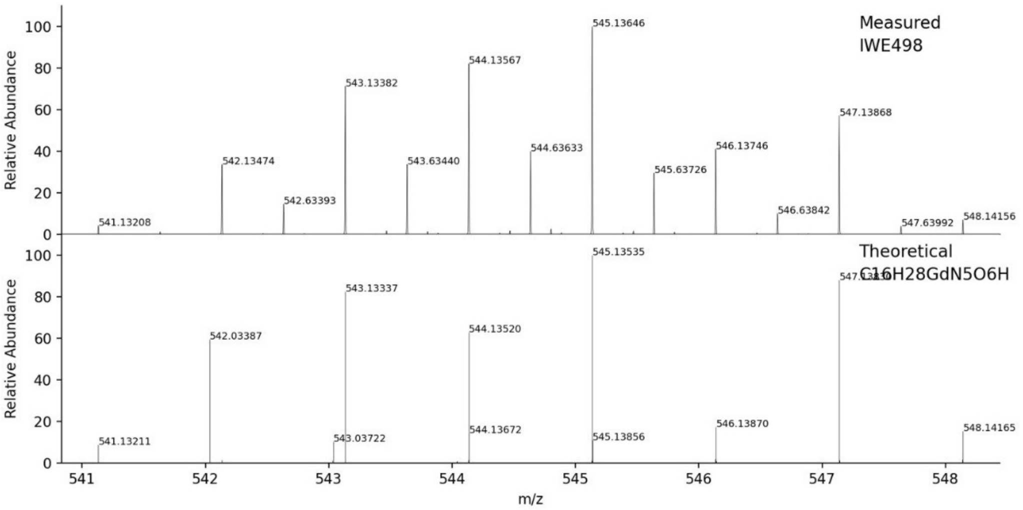

Figure S38. HRSM of **3-Gd**,. HRMS (ESI+) calc. for $[M+H]^+$ ($C_{16}H_{29}N_5O_6Gd^+$): 545.1353, found:545.1365.

10. LCMS

Below are the UPLC results of compounds: **9, 10, 6, 7, 8, 1, 1-Yb, 1-Gd, 3, 3-Gd, 3-Yb**

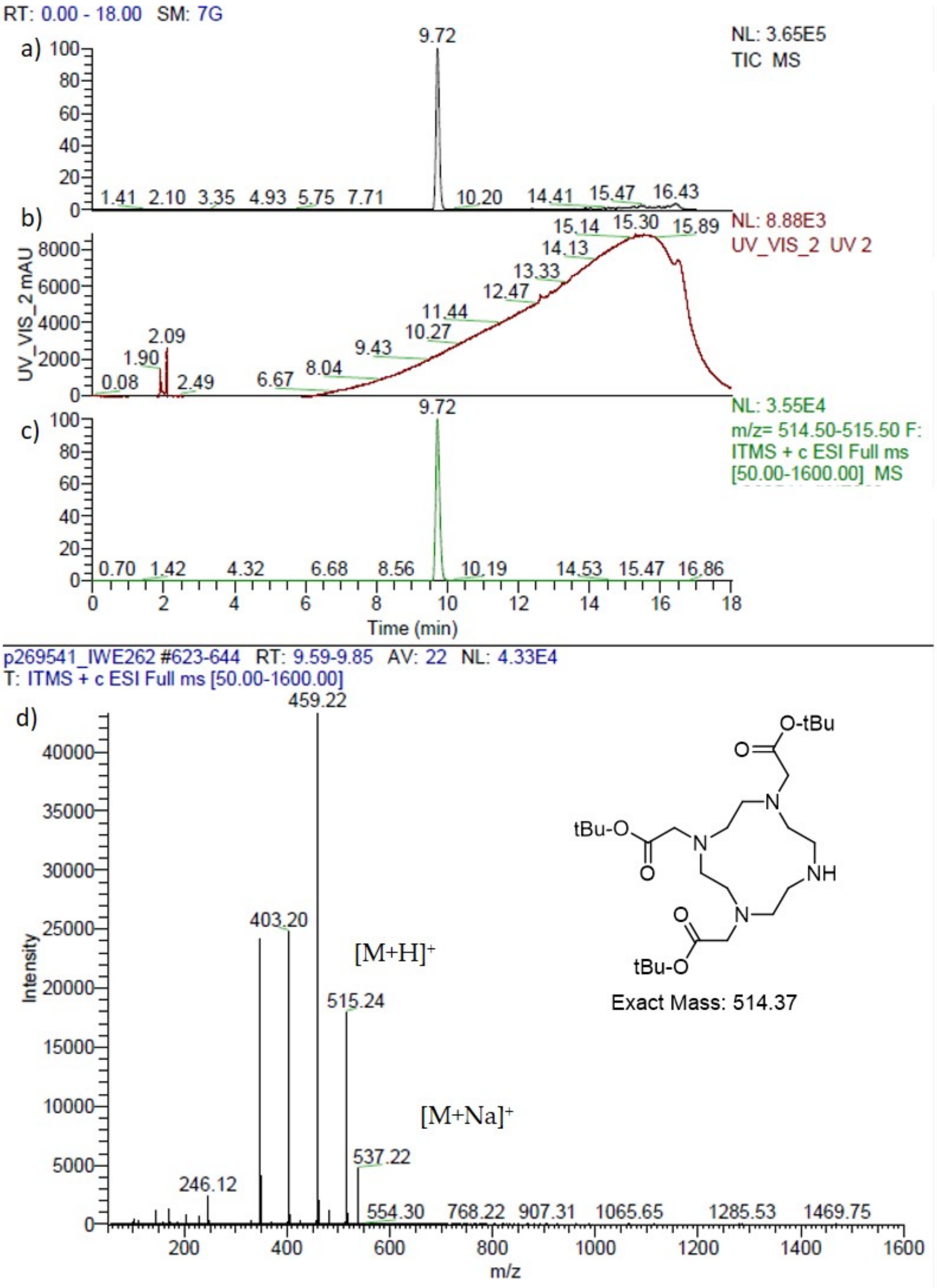

Figure S39. LCMS analysis of compound **9** using UPLC program 1 (see section 1), (a) total ion current chromatogram, (b) UV-vis at 254 nm chromatogram), (c) 514-515 mass trace, (d) mass spectrum of the peak at R_t = 9.7 min.

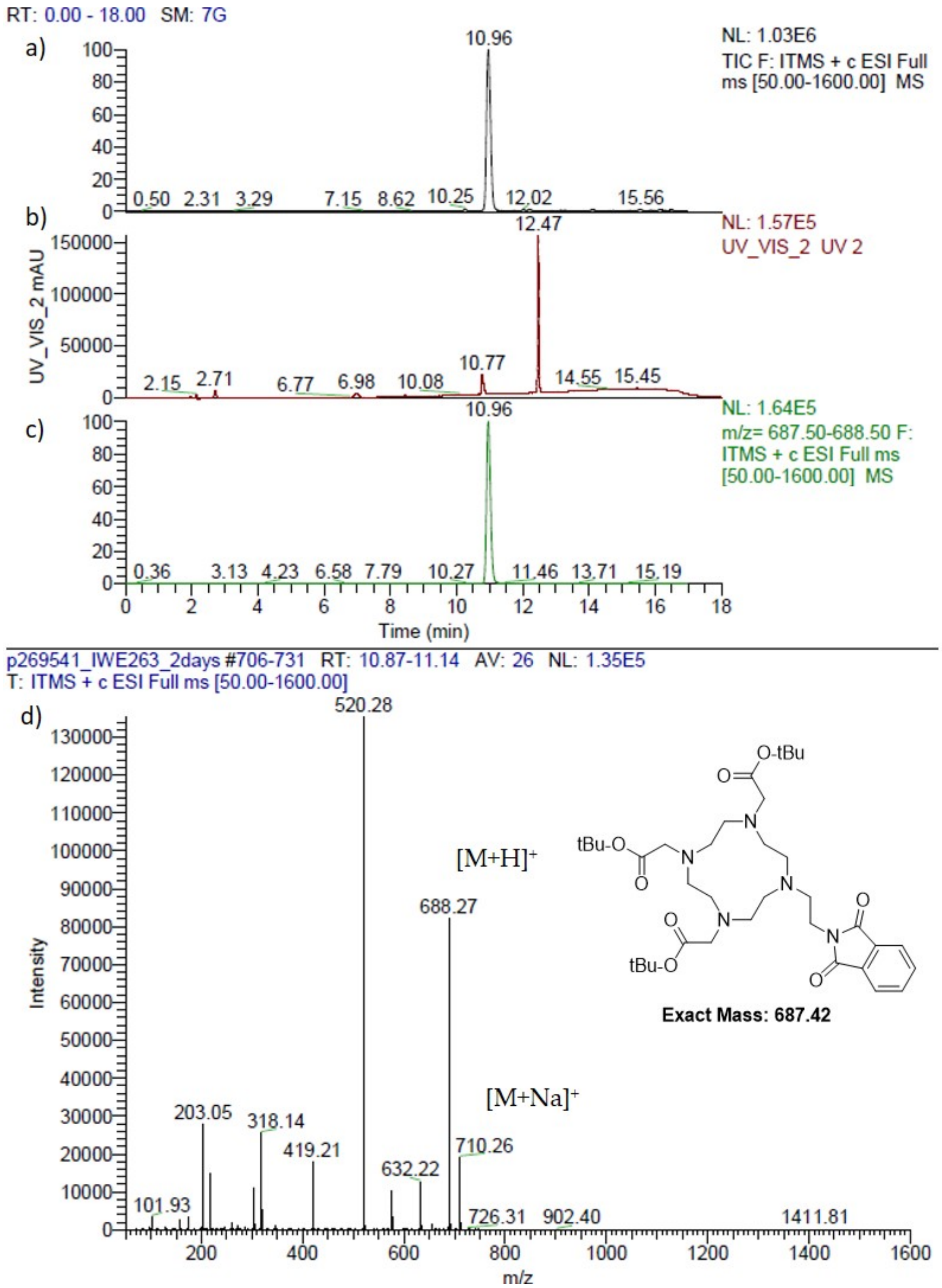

Figure S40. LCMS analysis of compound **10** using UPLC program 1 (see section 1), (a) total ion current chromatogram, (b) UV-vis at 254 nm chromatogram), (c) 687-688 mass trace, (d) mass spectrum of the peak at R_t = 10.96 min.

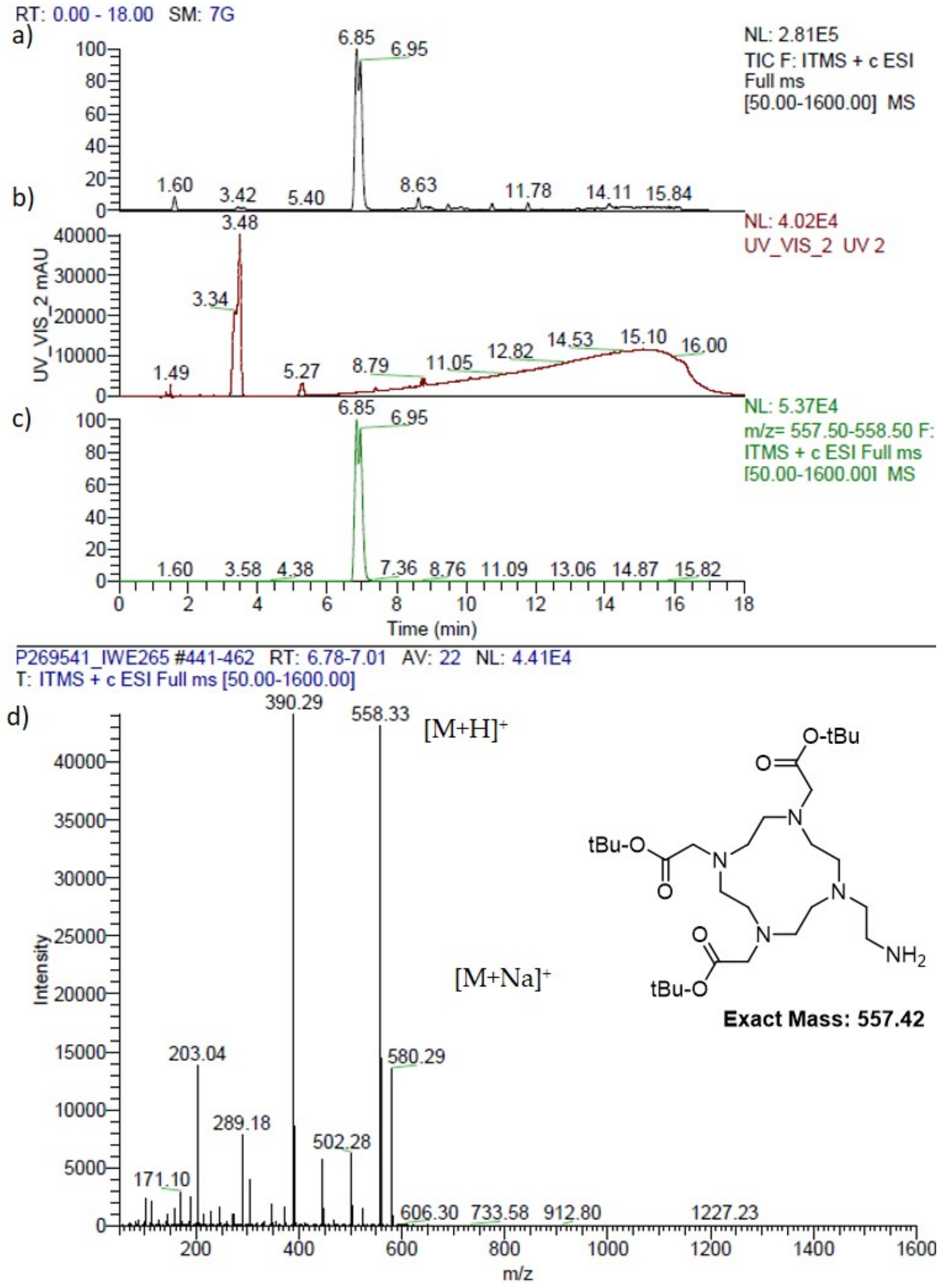

Figure S41. LCMS analysis of compound **6** using UPLC program 1 (see section 1), (a) total ion current chromatogram, (b) UV-vis at 254 nm chromatogram), (c) 557-558 mass trace, (d) mass spectrum of the peak at R_t = 6.85 min.

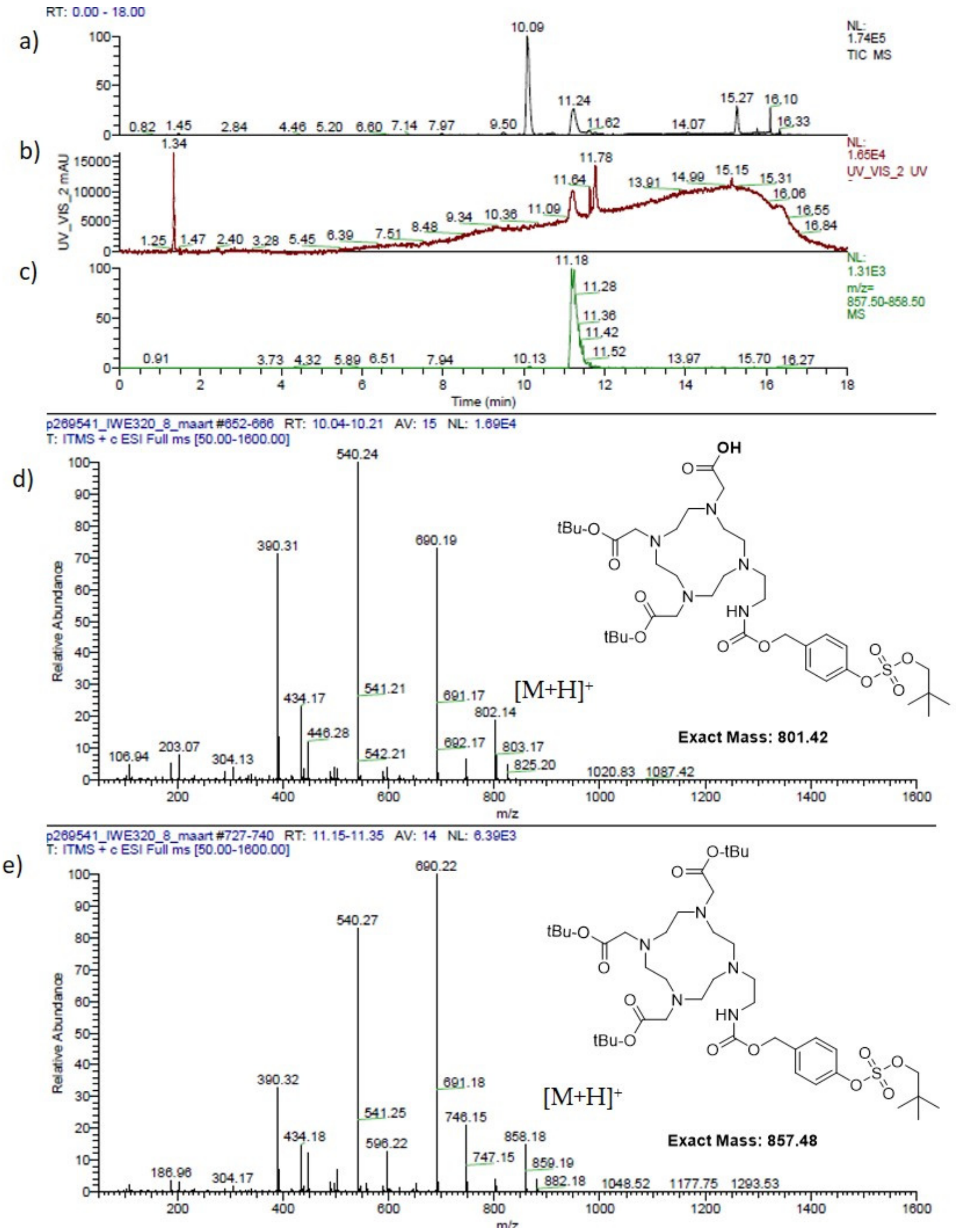

Figure S42. LCMS analysis of compound **7** using UPLC program 1 (see section 1), a) total ion current chromatogram, (b) UV-vis at 254 nm chromatogram), (c) 857-858 mass trace, (d) mass spectrum of the peak at rt = 10 min, (e) mass spectrum of the peak at R_t = 11.24 min.

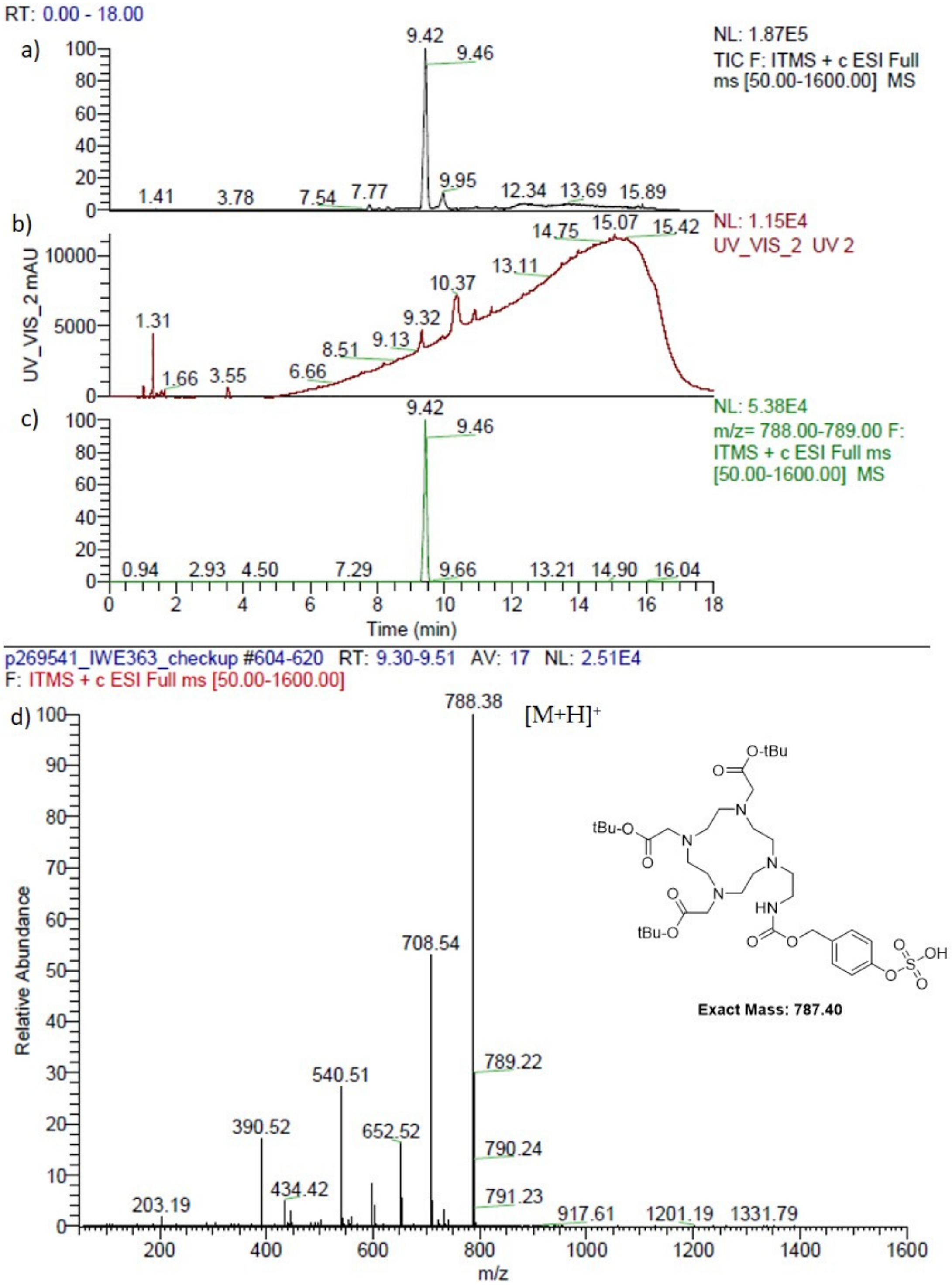

Figure S43. LCMS analysis of compound **8** using UPLC program 1 (see section 1), (a) total ion current chromatogram, (b) UV-vis at 254 nm chromatogram), (c) 788-789 mass trace, (d) mass spectrum of the peak at R_t = 9.52 min.

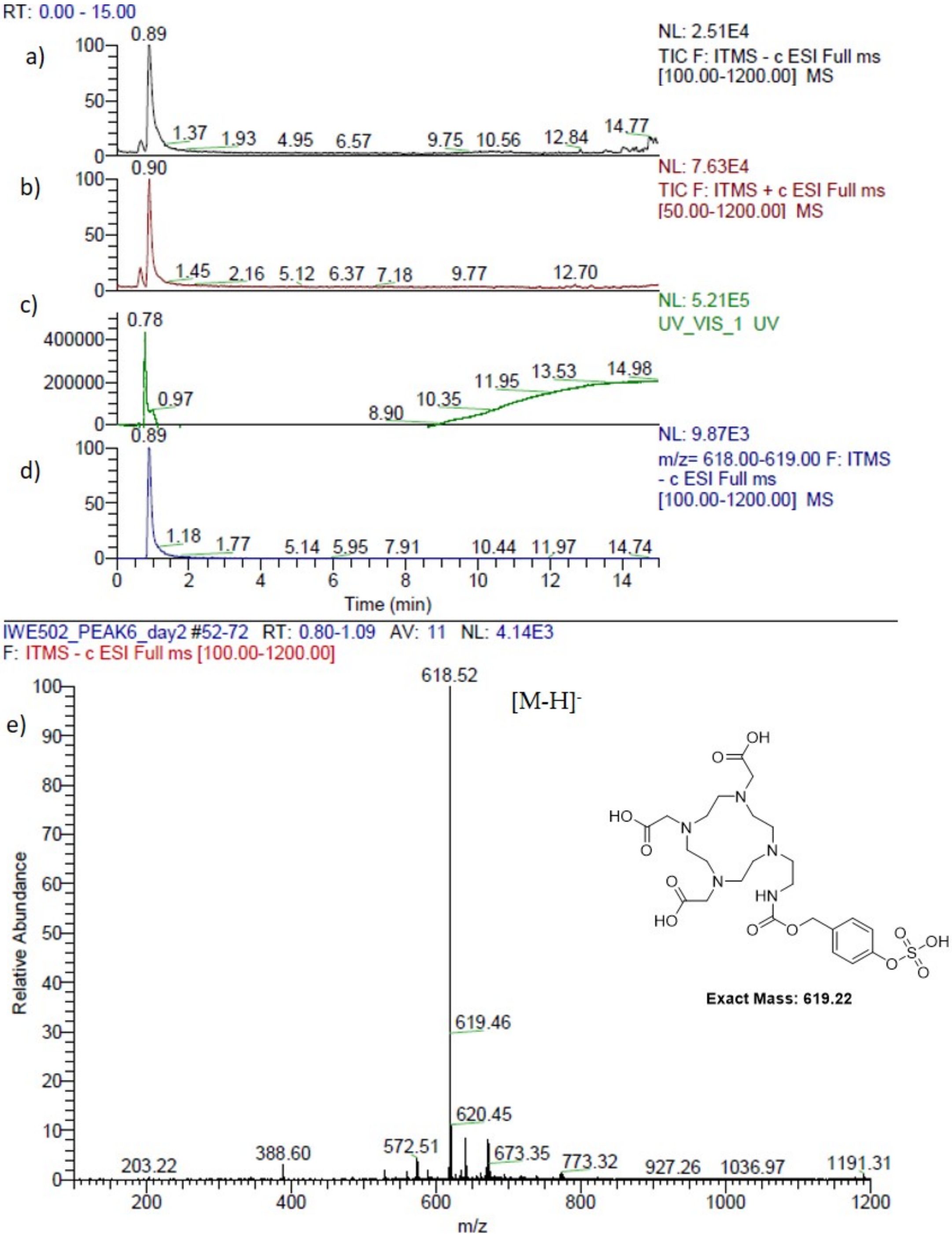

Figure S44. LCMS analysis of compound **1** using UPLC program 1 (see section 1), (a) total ion current chromatogram (negative mode), (b) UV-vis at 254 nm chromatogram), (c) 618-619 mass trace, (d) mass spectrum of the peak at R_t = 0.89 min.

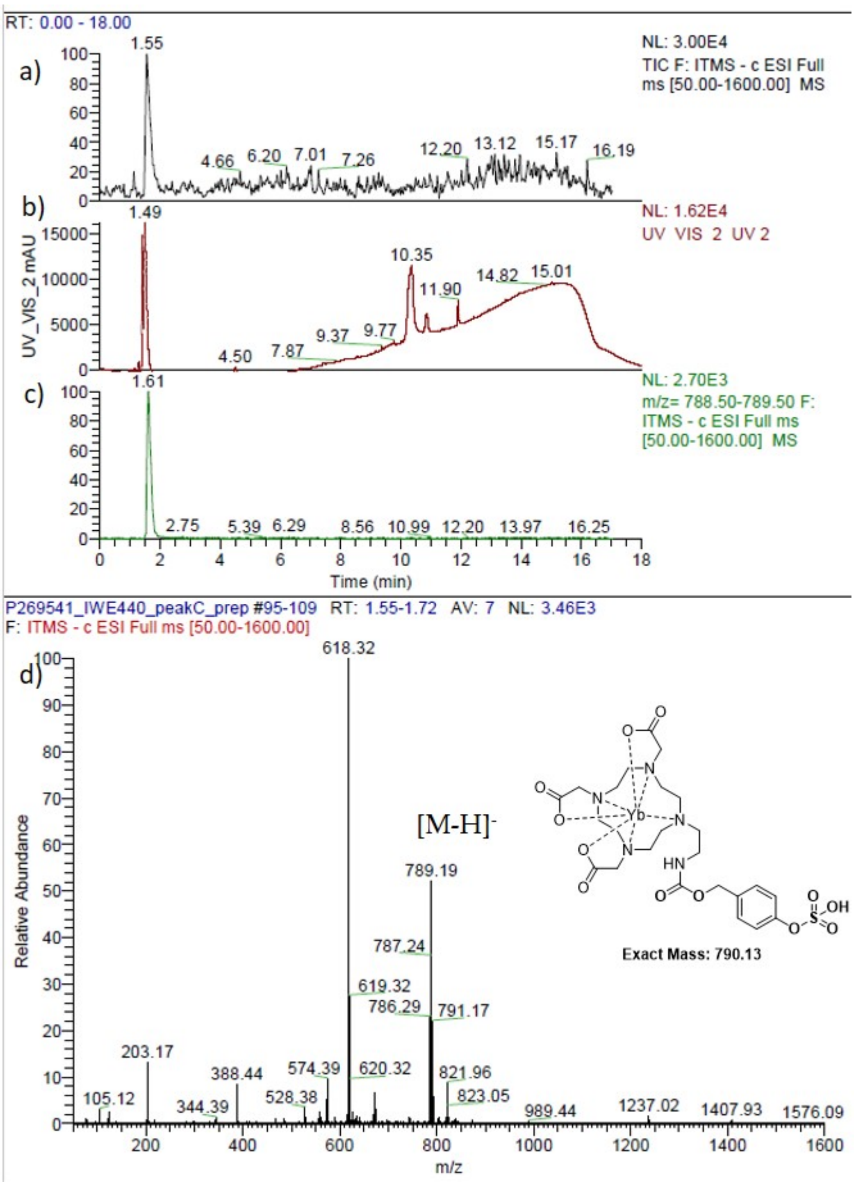

Figure S45. LCMS analysis of compound **1-Yb** using UPLC program 1 (see section 1), (a) total ion current chromatogram (negative mode), (b) UV-vis at 254 nm chromatogram), (c) 788-789 mass trace, (d) mass spectrum of the peak at R_t = 1.55 min.

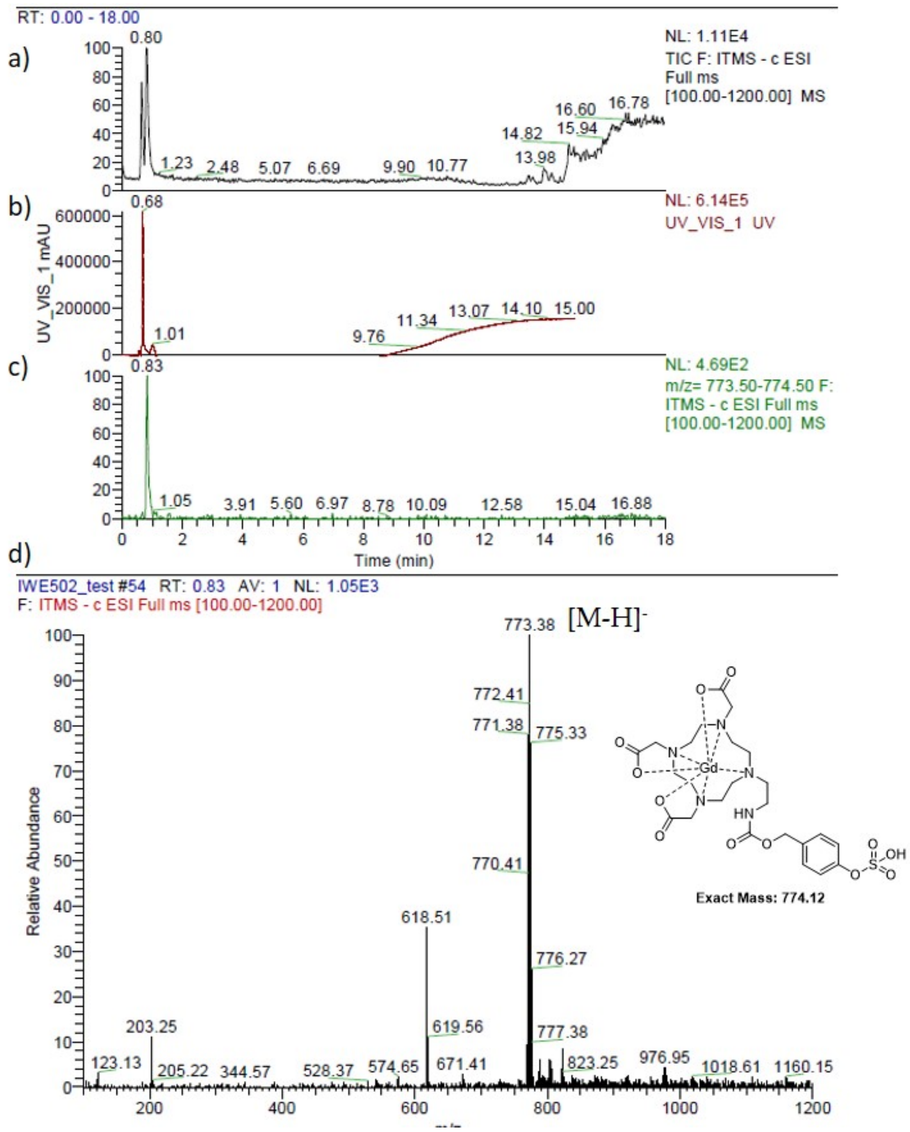

Figure S46. LCMS analysis of compound **1-Gd** using UPLC program 1 (see section 1), (a) total ion current chromatogram (negative mode), (b) UV-vis at 254 nm chromatogram), (c) 773 mass trace, (d) mass spectrum of the peak at R_t = 0.80 min.

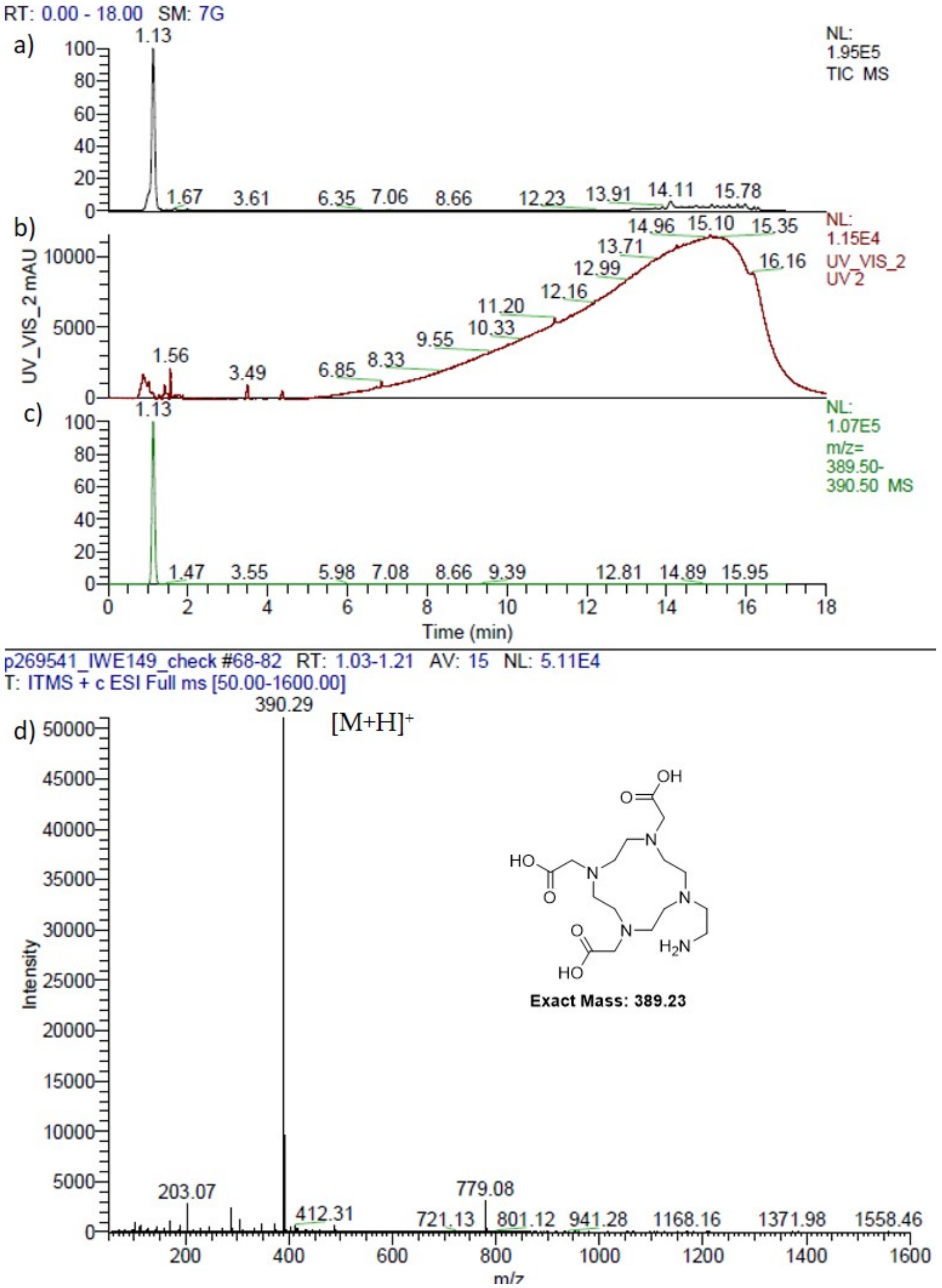

Figure S47. LCMS analysis of compound **3** using UPLC program 1 (see section 1), (a) total ion current chromatogram, (b) UV-vis at 254 nm chromatogram), (c) 389-390 mass trace, (d) mass spectrum of the peak at R_t = 1.13 min.

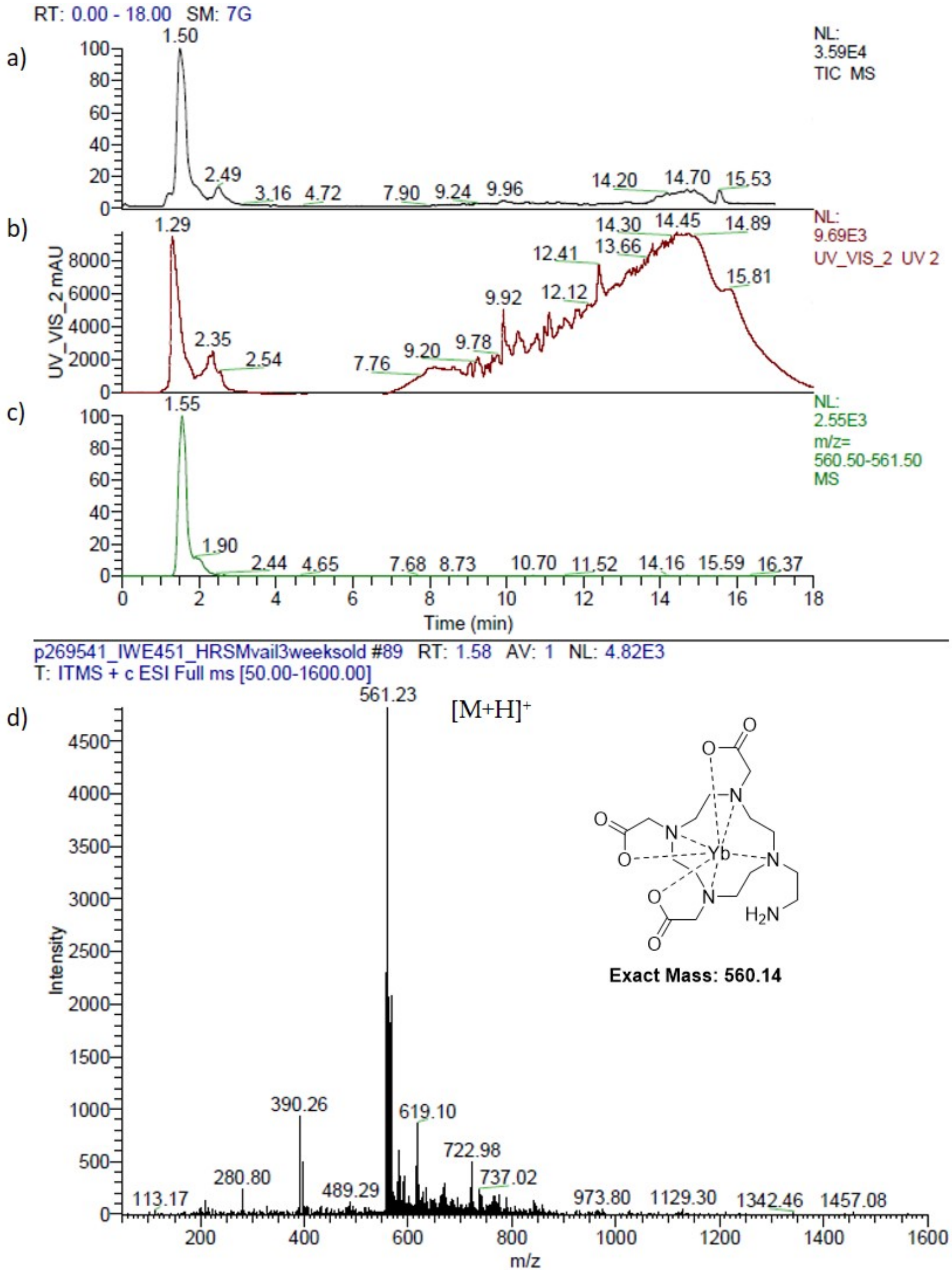

Figure S48. LCMS analysis of compound **3-Yb** using UPLC program 1 (see section 1), (a) total ion current chromatogram, (b) UV-vis at 254 nm chromatogram), (c) 560-561 mass trace, (d) mass spectrum of the peak at R_t = 1.50 min.

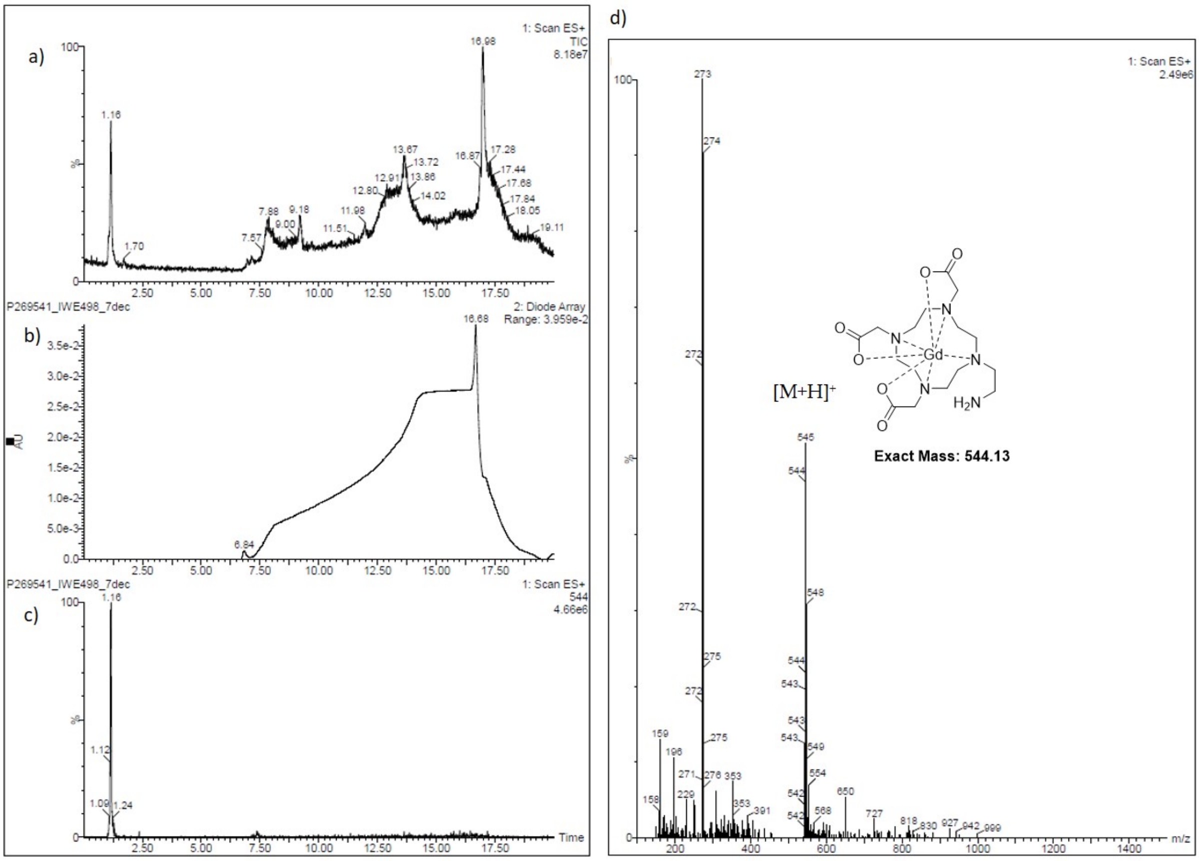

Figure S49. LCMS analysis of compound **3-Gd** using UPLC program 2 (see section 1), (a) total ion current chromatogram, (b) UV-vis at 254 nm chromatogram), (c) 544 mass trace, (d) mass spectrum of the peak at R_t = 1.16 min.

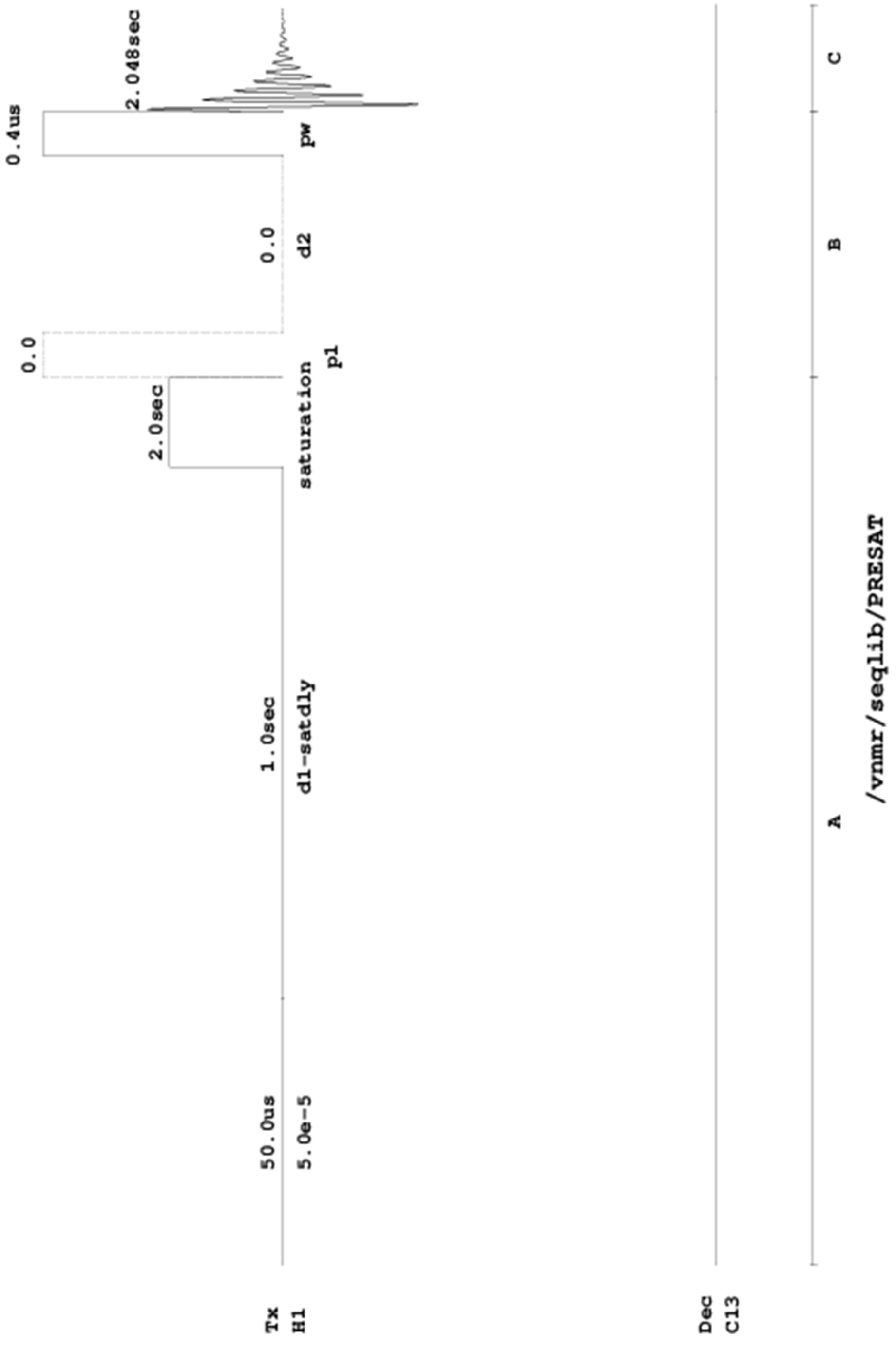

Figure S50. Pulse sequence used to measure the Z-spectra.

10. References

1. Simpson, L.S.; Widlanski, T.S. A comprehensive approach to the synthesis of sulfate esters. *J. Am. Chem. Soc.* **2006**, *128*, 1605–1610.
2. Wan, F.; Liu, M; Zhang, J.; Li, Y. and Jiang, L. Synthesis and characterization of DOTA-mono-adamantan-1-ylamide, *Res. Chem. Intermed*., **2015**, *41*, 5109-5119.
3. De La Reberdière, A.; Lachaud, F.; Chuburu, F.; Cadiou, C.; Lemercier, G. Synthesis of a new family of protected 1,4,7,10-tetraazacyclododecane-1,4, 7-triacetic acid derivatives with thioctic acid pending arms. *Tetrahedron Lett.* **2012**, *53*, 6115–6118.
4. Krchová, T.; Kotek, J.; Jirák, D.; Havlíčková, J.; Císařová, I.; Hermann, P. Lanthanide(iii) complexes of aminoethyl-DO3A as PARACEST contrast agents based on decoordination of the weakly bound amino group. *Dalt. Trans.* **2013**, *42*, 15735–15747.
5. Barge, A.;Cravotto, G; Gianolio, E and Fedeli, F. How to determine free Gd and free ligand in solution of Gd chelates. A technical note. *Contrast Media Mol. Imaging*, **2006**, *1*, 184-188.
